# Investigations into mechanism and origin of regioselectivity in the metallaphotoredox-catalyzed α-arylation of *N*-alkylbenzamides†

**DOI:** 10.1039/d2sc01962k

**Published:** 2022-08-19

**Authors:** Alexander W. Rand, Mo Chen, John Montgomery

**Affiliations:** Department of Chemistry, University of Michigan 930 N. University Ave. Ann Arbor MI 48109-1055 USA jmontg@umich.edu

## Abstract

A mechanistic study on the α-arylation of *N*-alkylbenzamides catalyzed by a dual nickel/photoredox system using aryl bromides is reported herein. This study elucidates the origins of site-selectivity of the transformation, which is controlled by the generation of a hydrogen atom transfer (HAT) agent by a photocatalyst and bromide ions in solution. Tetrabutylammonium bromide was identified as a crucial additive and source of a potent HAT agent, which led to increases in yields and a lowering of the stoichiometries of the aryl bromide coupling partner. NMR titration experiments and Stern–Volmer quenching studies provide evidence for complexation to and oxidation of bromide by the photocatalyst, while elementary steps involving deprotonation of the *N*-alkylbenzamide or 1,5-HAT were ruled out through mechanistic probes and kinetic isotope effect analysis. This study serves as a valuable tool to better understand the α-arylation of *N*-alkylbenzamides, and has broader implications in halide-mediated C–H functionalization reactions.

## Abstract 2

A mechanistic study of the α-arylation of *N*-alkylbenzamides catalyzed by a dual nickel/photoredox system using aryl bromides elucidates the origins of site-selectivity of the transformation and identifies the hydrogen atom transfer agent.

## Graphical abstract

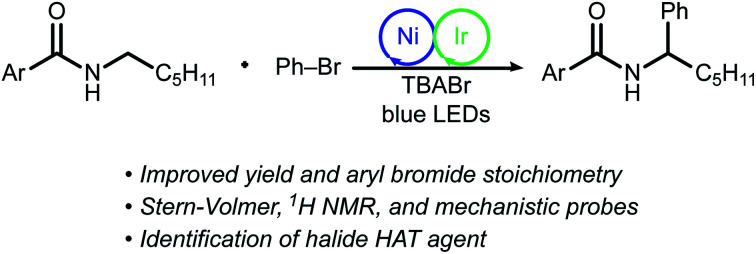

## Introduction

Site-selective C–H functionalization reactions allow streamlined access to valuable products in an efficient manner from commonly available starting materials.^1^ The ability to differentiate C–H bonds that are in similar steric and electronic environments is a challenge that requires both the development of new reactions and a better understanding of existing mechanistic underpinnings.^2^ The exploration of strategies in selective functionalization targeted towards specific C–H bonds, however, often rely on directing groups,^3^ which can sometimes be tedious to install and remove in cases where the directing functionality is not desired in the final structure.

Complementary to transition metal-catalyzed approaches, processes involving proton-coupled electron transfer (PCET) and hydrogen atom transfer (HAT) initiated by a photocatalyst have allowed for the selective functionalization of sp^3^ C–H bonds.^4^ Notably, independent studies by Rovis^5^ and Knowles^6^ showed that PCET could be used to alkylate distal C–H bonds using Michael acceptors by leveraging an amidyl radical followed by a 1,5-HAT. Several other groups, including Rovis,^7^ Alexanian,^8^ Tambar,^9^ Martin,^10^ Nagib,^11^ and Roizen^12^ showed that similar strategies could generate either distal or proximal alkylation/allylation under a variety of conditions. Despite these important developments in the functionalization of unactivated C–H bonds, the origins of site-selectivity have often been elusive.

**Scheme 1:**
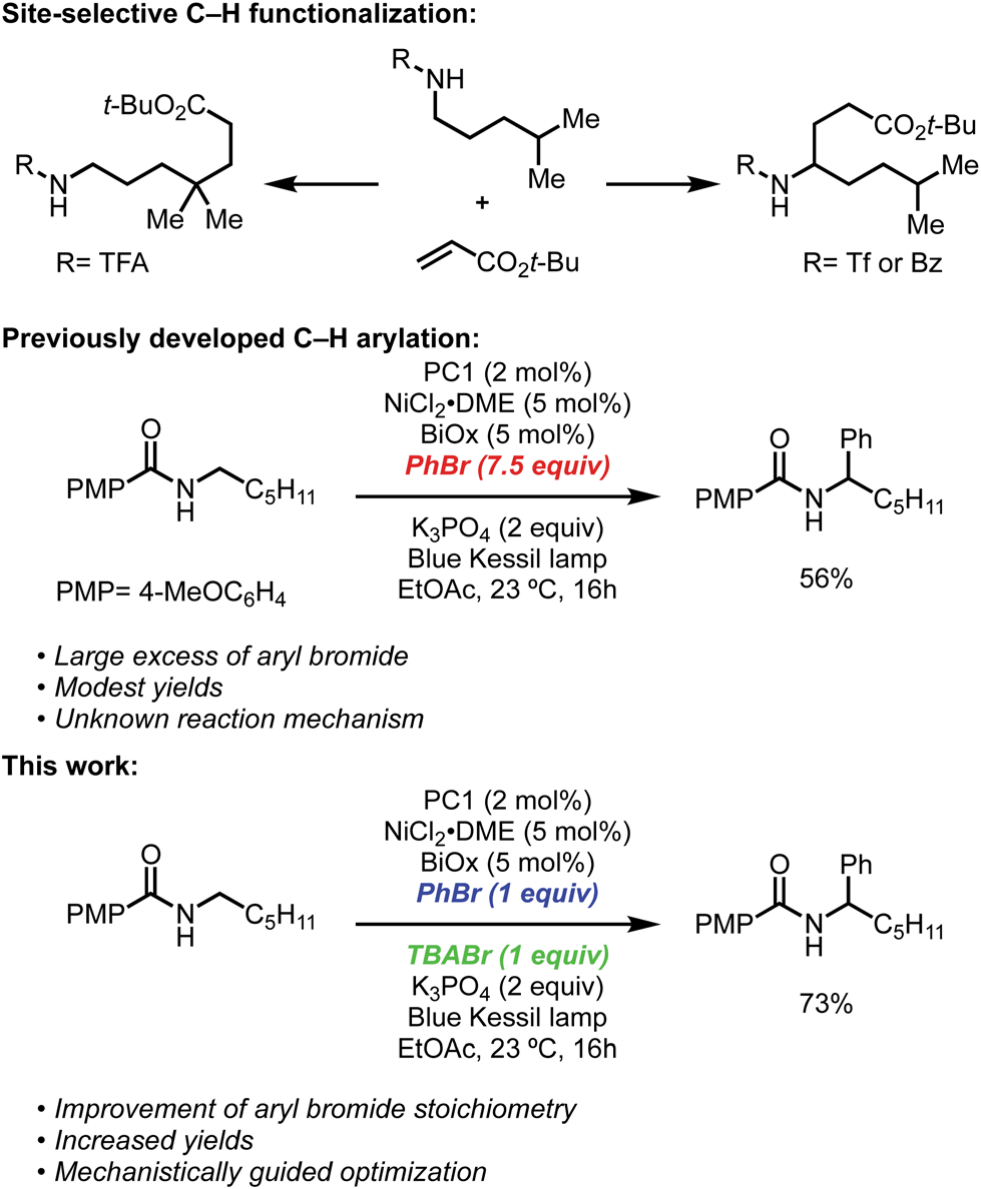
Site-selective C–H functionalization of protected amines.

Recently, our group and the Martin lab collaboratively reported a metallaphotoredox-catalyzed α-arylation and alkylation of *N*-alkylbenzamides using aryl or alkyl bromides (Scheme 1).^13^ This methodology was found to preferentially activate aryl bromides in the presence of aryl or alkyl chlorides and could tolerate a number of sensitive functional groups including boronic esters, alkyl amides, and challenging heterocyclic units including pyridines and thiophenes. Furthermore, the unique site-selectivity was highlighted through the orthogonal α- and δ-functionalization of a common starting material using either our method or conditions developed by Knowles.^6^ Lastly, this method was rendered asymmetric through the use of a chiral bioxazoline (BiOx) ligand, which allowed for high enantioinduction when conducted at low temperatures.

Despite the broad scope for both the *N*-alkylbenzamide and bromide coupling partner, these reactions, in some cases, suffered from low conversions and often required a large excess of either component to attain synthetically useful yields. During the optimization, it was noted that the reactions exhibited unique profiles that led us to believe a different mechanism than that reported by Rovis^7*a*^ and Tambar^9^ was responsible for the observed regio- and chemoselectivity. Among these observations, nickel(ii) salts vastly outperformed nickel(0) sources such as Ni(COD)_2_, strong inorganic and amine bases completely inhibited these reactions, and the insolubility of the base (K_3_PO_4_) did not have a negative effect on the reaction. Furthermore, the use of solvents that contained weak C–H bonds, such as DMF and THF, consumed the aryl bromide through deleterious solvent functionalization, which led us to use more robust solvents such as EtOAc.

In recent years, there has been a renewed interest in understanding the underpinnings of metallaphotoredox reactions.^14^ Among these studies, small deviations in procedures have resulted in substantial changes in regio- and chemoselectivity, as well as new modes of reactivity. In our pursuit to improve upon this reaction through a fundamental understanding of its mechanism, we describe a reaction pathway that better explains the exquisite site-selectivity, the requirements for specific nickel pre-catalyst, and the requirement for relatively large excesses of one of the coupling partners. Based on investigations into the reaction mechanism, we describe here that tetrabutylammonium bromide (TBABr) serves as an integral additive, leading to yield improvements while simultaneously allowing each coupling partner to be used in near-stoichiometric ratios. A series of mechanistic experiments, quenching, and titration studies described herein provide a greater understanding of the role of key additives, the nature of the C–H abstraction agent, and the origin of site-selectivity. We anticipate these insights will provide a foundation for other metallaphotoredox systems and will enable the reactivity trends to be applied in new classes of reactions.

## Results and discussion

### PCET/1,5-HAT

**Scheme 2:**
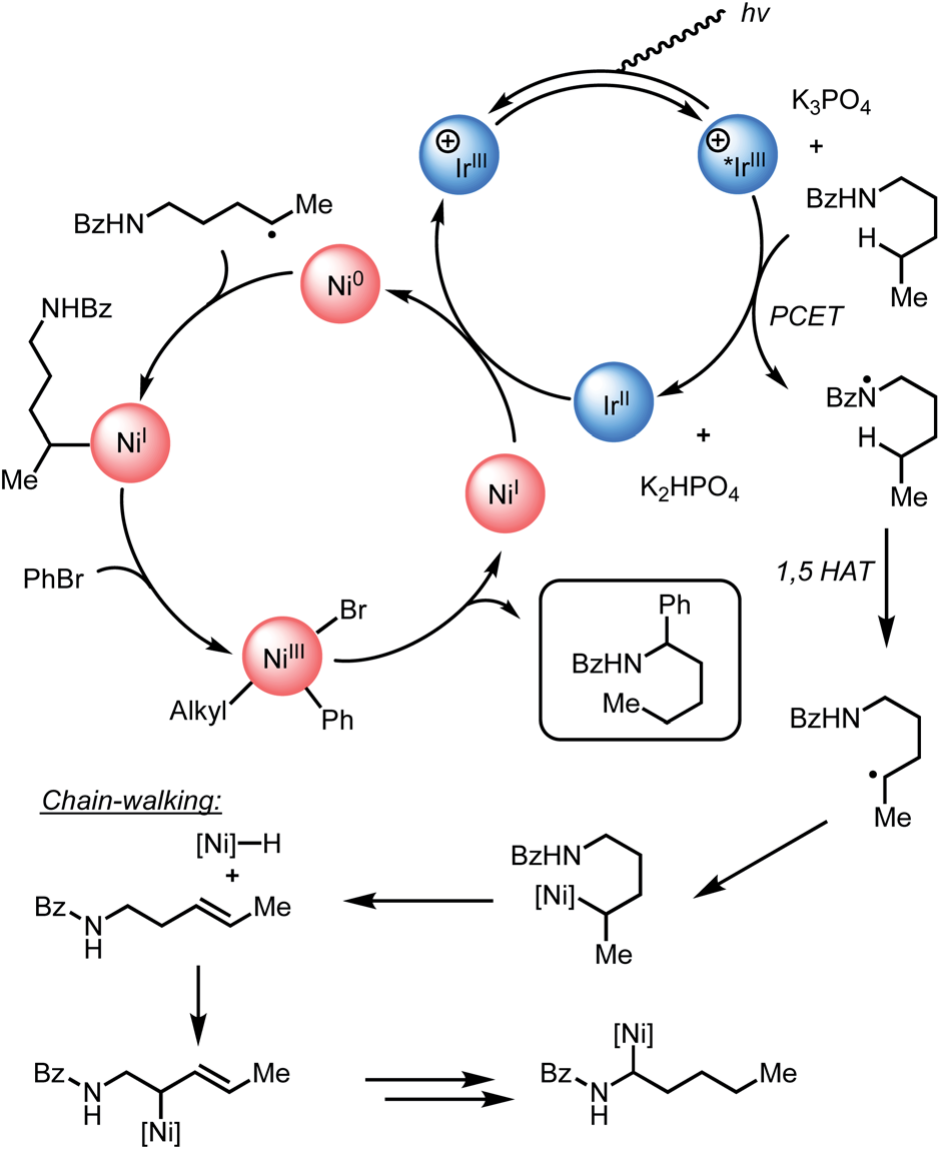
Initial mechanistic hypothesis.

**Scheme 3:**
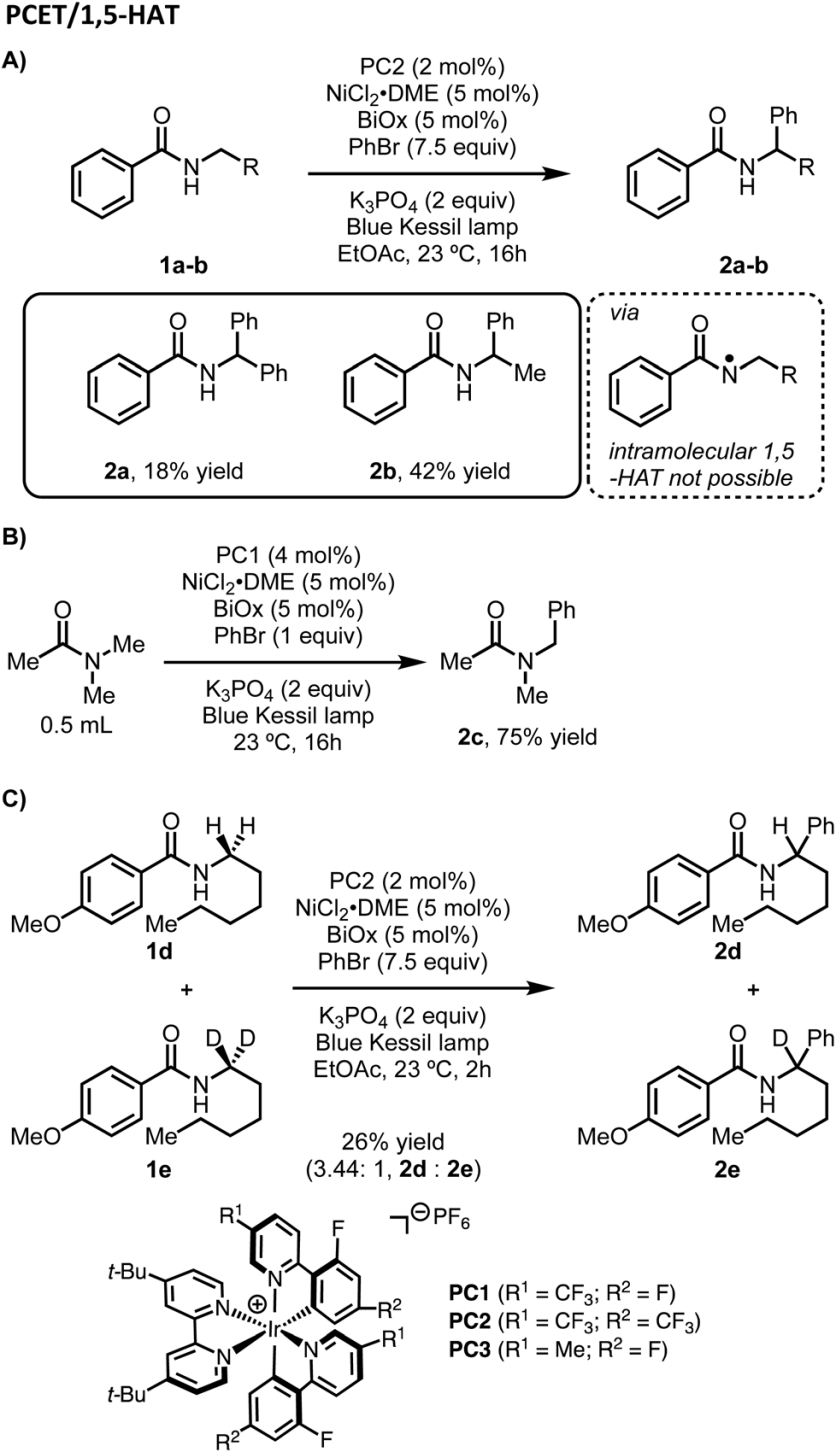
Observed regioselectivity and kinetic isotope studies for the α-arylation of *N*-alkylbenzamides.

Considering our initial results and evidence in the literature on amide C–H bond functionalization using photoredox catalysis, we set out to develop a working hypothesis to explain the observed regioselectivity for our metallaphotoredox-catalyzed α-arylation of *N*-alkylbenzamides using aryl bromides. We initially considered the possibility that the reaction was initiated by a PCET between the photocatalyst, *N*-alkylbenzamides, and K_3_PO_4_ to afford an *N*-centered radical, followed by 1,5-HAT to yield a distal carbon-centered radical (Scheme 2). From this distal radical, capture by a nickel(ii)ArBr species, followed by a series of β-hydride eliminations and reinsertions to chain-walk to the proximal α-position, could allow for reductive elimination to afford the observed products. The catalytic cycle could then be closed through electron transfer between the nickel and iridium photocatalyst. While exploring the substrate scope of this reaction, several inconsistencies were noticed. Namely, *N*-alkylbenzamides that lack distal C–H bond were suitable substrates in this reaction (Scheme 3A). Furthermore, tertiary amides did not provide appreciable yields of the desired product, though when used in solvent quantities, α-arylation could be observed, suggesting that a free N–H was not necessary for reactivity (Scheme 3B). To probe for a PCET/1,5-HAT as observed by Rovis and Knowles, a substrate containing deuterium at the α-position was subjected to the reaction conditions (Scheme 3C). In the initial report, using α,α-dideutero and α,α-diproteo *N*-alkylbenzamides, a primary kinetic isotope effect (KIE) was observed suggesting that C–H bond cleavage is involved at or before the turnover-limiting step of the catalytic cycle.^13^ Furthermore, deuterium scrambling along the alkyl chain of this substrate was not observed. If the reaction proceeded through the formation of a distal radical followed by a chain-walking sequence to arrive at an α-nickel species, a KIE would not be expected, and complete retention of deuterium at the α-position would not be observed. Taken together, these results suggest that PCET followed by 1,5-HAT is not operative under these reaction conditions.

### Deprotonation

**Scheme 4:**
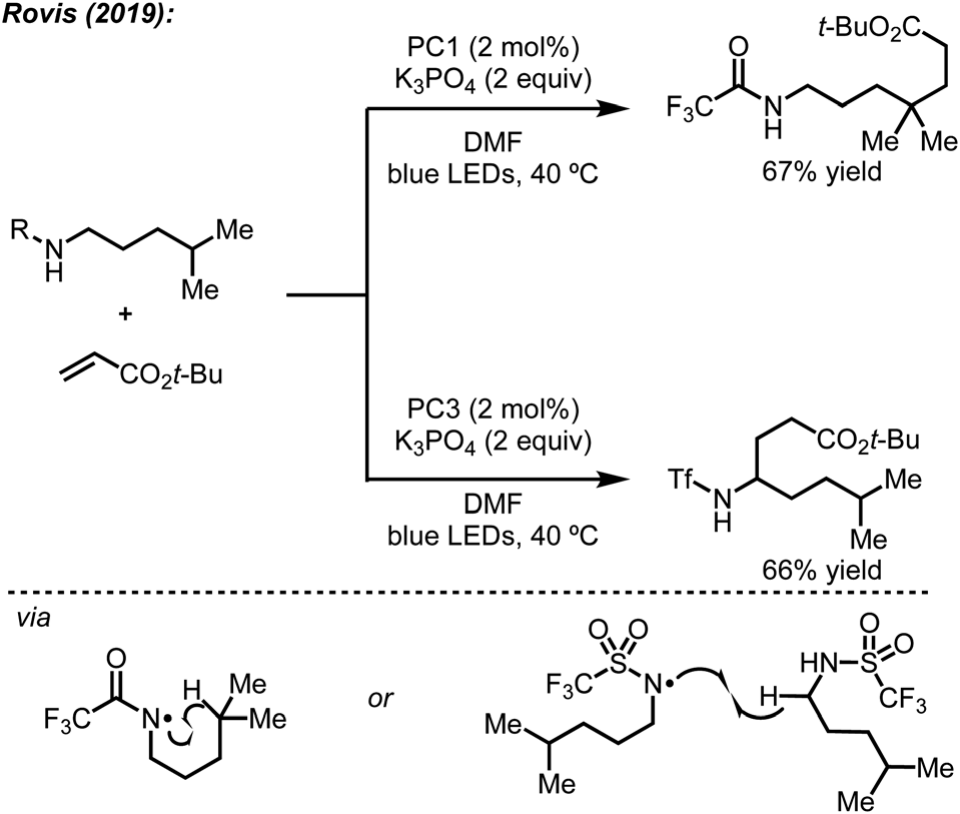
Effects on regioselectivity using different nitrogen protecting groups observed by Rovis.

In addition to their original report of δ-alkylation of trifluoroacetamides with electron deficient olefins,^5^ the Rovis group, among others, have developed several protocols for the site-selective functionalization of amide derivatives.^7*c*^ Specifically, by switching from a trifluoroacetamide to a triflamide, α-alkylation became possible under otherwise identical conditions (Scheme 4).^7*b*^ This change in regioselectivity was attributed to the lower p*K*_a_ of the triflamide, which allows for deprotonation by K_3_PO_4_ followed by oxidation by the photocatalyst to form a triflamidyl radical. The change in site-selectivity was attributed to the stability of the triflamidyl radical, which is persistent enough to undergo intermolecular HAT with a second equivalent of triflamide.

**Scheme 5:**
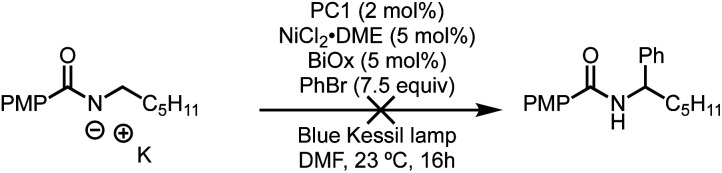
Unsuccessful α-arylation with deprotonated benzamides.

Based on these past findings, we considered that deprotonation of an *N*-alkylbenzamide under the reaction conditions might be responsible for the observed regioselectivity. To evaluate this pathway, the potassium salt of *N*-hexyl-4-methoxybenzamide was synthesized and tested under our reaction conditions for α-arylation. Surprisingly, this substrate did not provide the desired product, and the unreacted *N*-alkylbenzamide was recovered quantitatively (Scheme 5). This suggests that deprotonation of the *N*-alkylbenzamides in this reaction is not a productive pathway on the catalytic cycle, and in fact inhibits the reaction. These results could also explain why using K_3_PO_4_, which is sparingly soluble in the reaction mixture, still provides the desired product. Having established that the observed regioselectivity is not controlled through deprotonation of the *N*-alkylbenzamide, we next turned to Stern–Volmer quenching studies to elucidate the origins of radical formation and reaction initiation.

### Stern–Volmer studies

**Scheme 6:**
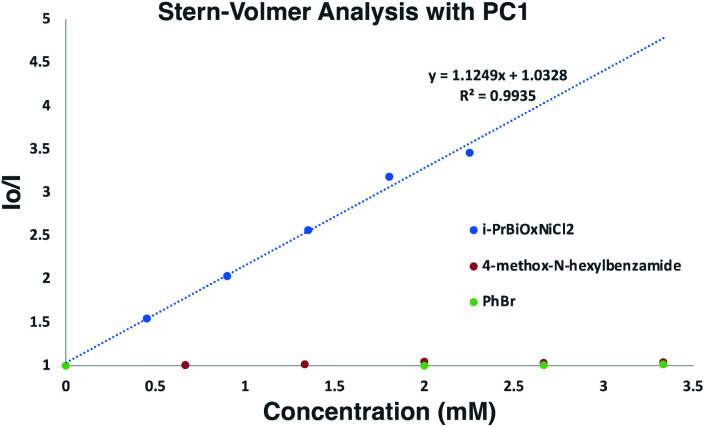
Stern–Volmer analysis of α-arylation reaction components. Linear quenching of PC1 observed with *i*-PrBiOxNi(ii)Cl_2_.

To gain insights into which reaction components were interacting with the photocatalyst for the α-arylation of *N*-alkylbenzamides, Stern–Volmer quenching studies were next conducted (Scheme 6). Beginning with *N*-hexyl-4-methoxybenzamide (*E*_p/2_ = +1.78 V *vs.* SCE), no quenching event was observed, which was expected since its redox potential lies outside the range of PC1 (*E*_1/2_^red^ Ir(iii*/ii) = +1.21 V *vs.* SCE). Similar results were observed when using PhBr (*E*_p/2_ = −1.76 V *vs.* SCE) with which reduction is also endergonic using PC1 (*E*_1/2_^ox^ Ir(iii*/iv) = −0.89 V *vs.* SCE). While K_3_PO_4_ could not be tested in these experiments due to its insolubility in EtOAc and DMF, it was found that *i*-PrBiOxNiCl_2_ strongly quenched the photocatalyst. Analysis of the CV of *i*-PrBiOxNiCl_2_ revealed a reduction event, *E*_p/2_ Ni(ii/i) = −0.83 V *vs.* SCE, which is thermodynamically feasible with PC1 and also with the reduced form of PC1 (*E*_1/2_^ox^ Ir(iii/ii) = −1.37 V *vs.* SCE).

Given the strong interaction between *i*-PrBiOxNiCl_2_ and PC1, we posit that electron transfer from Ir(iii*) to Ni(ii) would generate Ir(iv), Ni(i), and a halide anion. As halide anions have been shown to undergo oxidation by iridium polypyridyl photocatalysts,^15^ we envisioned that this newly generated halide anion could be oxidized by Ir(iv) to form a halide radical capable of abstracting weak C–H bonds (TBACl: *E*_p/2_^ox^ = +1.01 *vs.* SCE in CH_3_CN; *E*_1/2_^red^ Ir(iv/iii) = +1.69 V *vs.* SCE), such as those found in *N*-hexyl-4-methoxybenzamide.^16^ We suspected that the lower concentration of halide HAT agents at the beginning of the reaction might lead to sluggish and deleterious off-cycle reactions that necessitate the use of superstoichiometric coupling partners. Since only aryl bromides provided appreciable amounts of product, we envisioned that after initiation of the reaction, Br^−^ derived from PhBr was a likely HAT agent. Further supporting this hypothesis, the Doyle group demonstrated the effectiveness of TBABr as an HAT agent in a nickel/photoredox-catalyzed coupling through C–H abstraction of an acetal.^17^

Based on our Stern–Volmer quenching studies, we believe that nickel serves to not only activate the aryl bromide for coupling, but also functions as a source of HAT agent for C–H functionalization through the generations of halide anions.^15*a*,16*a*,*b*,*d*,18,19,20,21^ Under this premise, we began exploring the effect of adding exogenous halide salts to our model reaction in order to more efficiently generate reactive HAT agents (Table 1). As shown previously, aryl bromides were the only aryl substrates that provided the desired product (Table 1, entries 1–3). However, when adding 1 equivalent of TBABr to a reaction containing PhCl, we were delighted to see 30% yield of the desired product (entry 4).^19*a*,22^ While nickel has been shown to activate PhCl, PhBr, and PhI at room temperature, we believe that the addition of TBABr facilitates more facile access to halide radicals through SET with PC1 (TBABr *E*_p/2_^ox^ = +0.71 *vs.* SCE in CH_3_CN and TBACl *E*_p/2_^ox^ = +1.01 *vs.* SCE in CH_3_CN).^23^ The addition of TBACl did not have a large effect on the reaction when using PhBr (entry 5). TBAI can also be oxidized by PC1 (TBAI *E*_p/2_^ox^ = +0.26 *vs.* SCE in CH_3_CN), but the lower BDE of H–I compared to H–Br (71 kcal mol^−1^*vs.* 87 kcal mol^−1^) might explain why iodide is not an effective HAT agent in this reaction (entry 6). Similarly, examining the thermodynamics for HAT from Br˙ (BDE H–Br = 87 kcal mol^−1^) could explain the why only α-arylation is observed (H_3_C(O)NHC–H(CH_3_)_2_ BDE = 92 kcal mol^−1^*vs.* (H_3_C)_2_CH–H BDE = 99 kcal mol^−1^).^24^

**Table 1:** Effects of aryl halides and additives on α-arylation

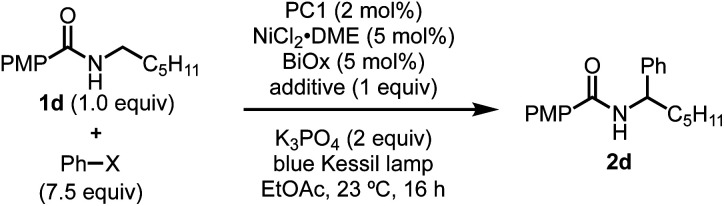			
Entry	X	Additive	Yield^a^
1	Cl	None	0%
2	Br	None	49%
3	I	None	0%
4	Cl	TBABr	30%
5	Br	TBACl	46%
6	I	TBABr	0%

a Yield determined using GCFID with tridecane as an internal standard.

**Scheme 7:**
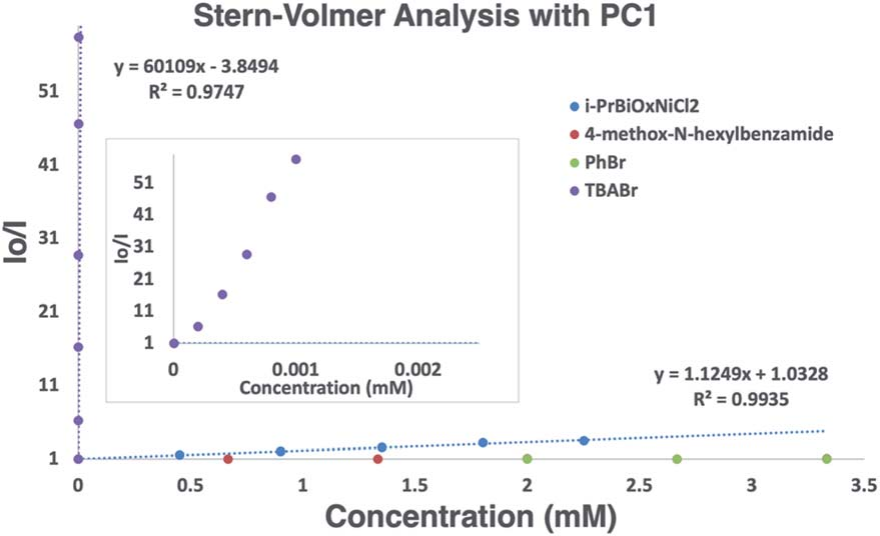
Stern–Volmer plot of reaction components with an expansion for TBABr. Strong, non-linear quenching of PC1 observed with TBABr suggesting static and dynamic quenching. Quenching was also observed with *i*-PrBiOxNi(ii)Cl_2_, while PhBr and 4-methoxy-*N*-hexylbenzamide did not quench PC1.

**Scheme 8:**
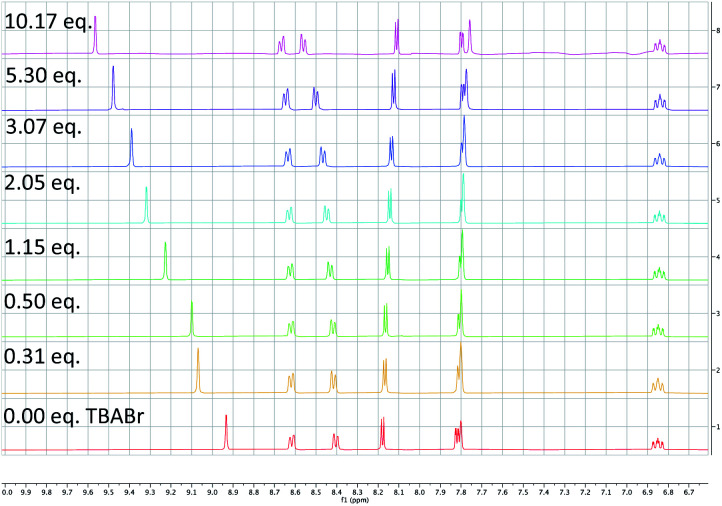
^1^H NMR of PC1 upon titration with TBABr. A large downfield shift of the C_3_–H of the bipyridyl ligand was observed at increasing concentrations of TBABr suggesting a complexation of Br^−^ to PC1.

To probe the feasibility of halide radical generation through the oxidation of a halide anion by a photocatalyst, we conducted Stern–Volmer quenching studies using an exogenous halide salt (Scheme 7). When conducting Stern–Volmer quenching studies using TBABr, we observed strong, static and dynamic quenching of PC1 (*K*_s_ = 1.8 × 10^3^ M^−1^ S^−1^ and *K*_d_ = 2.9 × 10^4^ M^−1^ S^−1^), which is indicative of association of Br^−^ to PC1 prior to electron transfer as well as intermolecular electron transfer from PC1 to Br^−^. Similar observations and magnitudes have been seen in metallaphotoredox systems that employ halide salts as HAT agents.^16*b*^ Corroborating the observed dynamic quenching, it was also observed through NMR titration experiments that, similar to Knowles and Alexanian,^8^ a new iridium species was formed when mixing PC1 and TBABr (*K*_eq_ = 6.3 × 10^2^) (Scheme 8). We believe that counterion exchange between cationic PC1 and TBABr could form an iridium–bromide complex that would serve to bring together these two species in solution for more efficient electron transfer.^18,19*b*,*f*,25^ Taken together, these results support our hypothesis that Br^−^ formed under our reaction conditions could be oxidized to Br˙ by the photocatalyst (TBABr: *E*_p/2_^ox^ = +0.71 *vs.* SCE), which in turn serves as an HAT agent for the abstraction of C–H bonds from the substrate. This could also explain why neutral photocatalysts with similar excited-state redox potentials, such as 4CzIPN (*E*_1/2_^red^ (PC*/PC˙^−^) = +1.43 *vs.* SCE, *E*_1/2_^ox^ (PC˙^+^/PC*) = −1.18 *vs.* SCE),^26^ provided only trace product in this reaction, as electron transfer would require the photocatalyst and Br^−^ to come into proximity for electron transfer to occur.

### HAT agents

Having identified TBABr as an HAT agent capable of undergoing SET with PC1 to form Br˙, we began reevaluating the parameters of the reaction (Table 2). During our initial optimization, we determined that 7.5 equivalents of PhBr were necessary to achieve high yield of the desired α-arylation product (Table 2, entry 1), and when using an equal ratio of *N*-alkylbenzamide to PhBr only 23% of the desired product could be attained (entry 2). Through the inclusion of 1 equivalent of TBABr, it was found that using a 1 : 1 ratio of *N*-hexyl-4-methoxybenzamide and PhBr provided 52% yield of the desired product (entry 3). Lastly, when using PhBr as the limiting reagent, 1 equivalent of TBABr significantly improved the yield of α-arylation product from 27% to 73% yield (entries 4 and 5).

**Table 2:** Improved reaction efficiency using TBABr additive

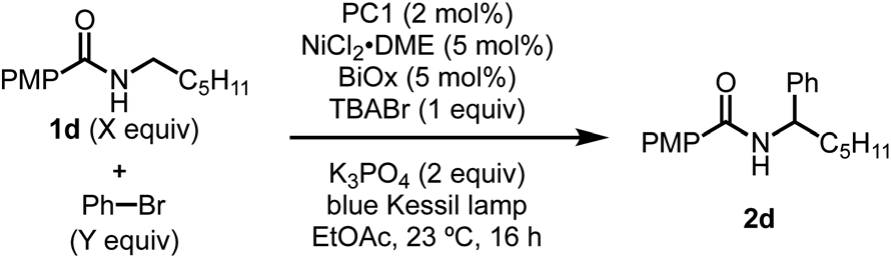			
Entry	X equiv. 1d	Y equiv. PhBr	Yield
1^a^	1	7.5	56%^b^
2^a^	1	1	23%^b^
3	1	1	52%
4^a^	2	1	27%^b^
5	2	1	73%^c^

a Run without TBABr.

b Yield determined by GCFID using tridecane as an internal standard.

c 9% of *N*-arylation observed.

Having observed higher yields when including of TBABr, this method was applied to several previously low-yielding reactions. Aryl bromides with ortho substituents typically lead to lower yields, but when including TBABr, the yield was increased from 33% using 7.5 equivalents of 2-bromotoluene to 41% when using the aryl bromide as the limiting reagent (entry 1, Table 3). Similary, yields when using electron-deficient aryl bromides such as 4-bromobenzotrifluoride and methyl-4-bromobenzoate increased from 53% to 64% and 49% to 67% yield (entries 2 and 3). Lastly, benzamides that previously gave low yields benefitted from the inclusion of TBABr, which resulted in an increase from 15% to 46% yield. These results show that including a HAT agent, such as TBABr, have a beneficial effect by improving reaction efficiency, which allows higher yields of the desired product with much lower excess of any reagent. When L_*n*_NiBr_2_ precatalysts are used, small yield improvements are seen by the addition of TBABr, in contrast to the more substantial yield and stoichiometry improvements seen with L_*n*_NiCl_2_ precatalysts (see ESI†).

**Table 3:** Improved yields using TBABr additive

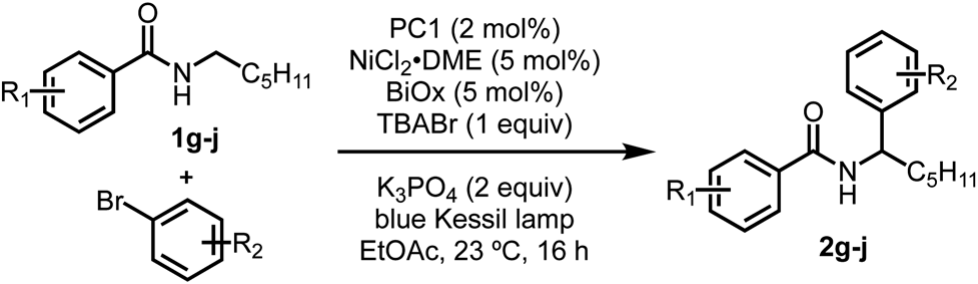			
Entry	R_1_	R_2_	% Yield^a^
1	4-OMe	2-Me	54 (41)
2^b^	4-OMe	4-CF_3_	71 (64)
3^b^	4-OMe	4-CO_2_Me	75 (67)
4	3-CO_2_Me	H	58 (46)

a Experiment was carried out with 0.2 mmol ArBr and 0.4 mmol benzamide. Yields were determined by ^1^H NMR using dibromomethane as an internal standard, yields in parentheses were isolated.

b Experiment was carried out at 0.1 mmol scale.

Based on spectroscopic and experimental evidence, we believe the addition of exogenous Br^−^ promotes facile C–H abstraction through the oxidation of Br^−^ by PC1 to generate Br˙, which is capable of abstracting the α-C–H bonds of *N*-alkylbenzamides^27^ and acetals.^17^ When TBABr is included in this reaction, the formation of higher concentrations of an iridium–bromide complex increases the efficiency of electron transfer between PC1 and Br^−^, which leads to more productive catalysis despite the lower concentration of PhBr. In the case where Br^−^ is not present at the beginning of the reaction, halide anions must be generated through reduction of *i*-PrBiOxNiX_2_ by PC1. When conducting this reaction without TBABr, only small quantities of Br^−^ are available for oxidation by PC1, which results in less productive catalysis.

### Proposed mechanism

Guided by the studies presented herein and building upon recent investigations on related systems,^13,16*a*,*b*,18*a*,19*b*,21,27,28^ we propose that the α-arylation of *N*-alkylbenzamides is initiated through the irradiation of PC1 to generate an excited-state Ir(iii*) species. Reduction of BiOxNi(ii)Cl_2_ (*i*-PrBiOxNi(ii)Cl_2_*E*_p/2_ (Ni(ii/i)) = −0.83 V *vs.* SCE) by PC1 (*E*_1/2_^ox^ Ir(iv/iii*) = −0.89 V *vs.* SCE) would form Ir(iv), BiOxNi(i)Cl and Cl^−^. Oxidation of Cl^−^ to Cl˙ or Br^−^ to Br˙ (TBACl *E*_p/2_^ox^ = +1.01 *vs.* SCE and TBABr: *E*_p/2_^ox^ = +0.71 *vs.* SCE in CH_3_CN) by Ir(iv) (*E*_1/2_^red^ Ir(iv/iii) = +1.69 V *vs.* SCE) are both thermodynamically favorable, and it has previously been proposed that the binding of halides to the 3,3′-position of bipyridine ligands of the photocatalyst provides a kinetic driving force for this redox event to occur.^8,18*a*,29^ Furthermore, we posit a halide radical would then perform an HAT on *N*-alkylbenzamide (I) to generate radical (II) and HX, which could be sequestered by K_3_PO_4_ in the reaction.

**Scheme 9:**
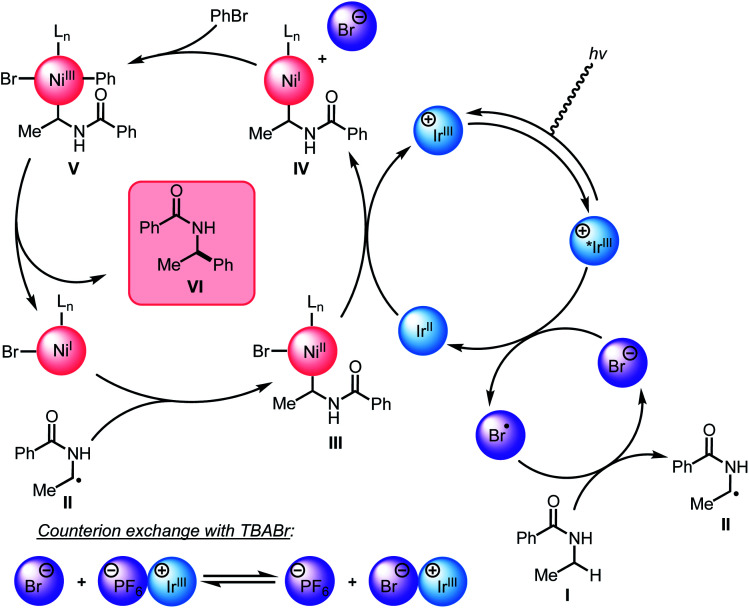
Revised mechanistic hypothesis.

After reduction of the Ni(ii) species by PC1, and HAT by a halide radical, II could then be captured by *i*-PrBiOxNi(i)Br to form *i*-PrBiOxNi(ii)alkylBr (III) (Scheme 9).^28^ At this point, reduction of *i*-PrBiOxNi(ii)alkylBr (III) by Ir(ii) (*E*_1/2_^red^ Ir(iii/ii) = −1.37 V *vs.* SCE) could generate a *i*-PrBiOxNi(i)alkyl species (IV) and Ir(iii). Ir(iii*) could then go on to oxidize another equivalent of Br^−^ to generate Ir(ii) and Br˙, which would then perform an HAT to continue the catalytic cycle. At this point, oxidative addition of PhBr to *i*-PrBiOxNi(i)alkyl (IV) would form *i*-PrBiOxNi(i)alkylPhBr (V), which would be poised for reductive elimination to generate *i*-PrBiOxNi(i)Br and the desired product.^30^ While the reaction is proposed to be initiated from *i*-PrBiOxNi(ii)Cl_2_, we envision that *i*-PrBiOxNi(i)Br generated under after the first catalytic cycle would serve as a suitable catalyst for this transformation.

Through these studies, we observed that the inclusion of TBABr leads to higher yields despite using lower concentrations of PhBr. Since exogenous Br^−^ is not necessary to produce the desired product, and the most likely HAT agent in the reaction after the initial catalytic cycle is Br˙, we believe that the primary benefit of TBABr is to more efficiently generate Br˙ through oxidation of Br^−^ by PC1, which serves to abstract the α-C–H bonds of *N*-alkylbenzamides, during the initiation of the reaction when substrate-derived bromide concentration is low. As evident from NMR titrations with PC1 and TBABr, when including TBABr in this reaction, the exogenous Br^−^ serves to form a higher concentration of Ir(iii)–Br complex in solution, which more efficiently promotes oxidation of Br^−^ to Br˙ by Ir(III*). Because the oxidation of Br^−^ to Br˙ is more favorable than the oxidation of the Cl^−^ to Cl˙ (TBABr: *E*_p/2_^ox^ = +0.71 *vs.* SCE and TBACl *E*_p/2_^ox^ = +1.01 *vs.* SCE in CH_3_CN),^15*a*,16*b*,31^ Br˙ is expected the be the predominate HAT agent throughout the initial and subsequent catalytic cycles when TBABr is present at the beginning of the reaction.

## Conclusions

This mechanistic study provides evidence that the α-arylation of *N*-alkylbenzamides using aryl bromides operates through an HAT mechanism that is mediated through the direct generation of a carbon-centered radical by Br˙ and a photocatalyst. The regio- and chemoselectivity observed in the reaction is governed by the BDEs of C–H bonds in the substrate and the ability of radical formation/HAT strength of different halide radicals. Evidence was gathered that refutes a PCET/1,5-HAT/chainwalking sequence or deprotonation/substrate oxidation sequence, and supports the notion that the reaction involves C–H abstraction by a halide radical. Evidence for electron transfer between PC1 and *i*-PrBiOxNi(ii)Cl_2_ and between PC1 and TBABr were supported by Stern–Volmer quenching studies. NMR titration studies highlight the formation of an iridium bromide species, which may facilitate more efficient oxidation of Br^−^ to Br˙. Based on these studies, improvements in reaction conditions were found using bromide additives that allowed for near equimolar stoichiometries of the *N*-alkylbenzamides and aryl bromides as well as increases in yield of α-arylation. Overall, this study improves access to valuable α-aryl-*N*-alkylbenzamides and expands the understanding of the role of additives and the origin of site-selectivity in metallaphotoredox-based C–H functionalization processes.

## Conflicts of interest

There are no conflicts to declare.

## Data availability

Supporting data has been provided as ESI† associated with this article.

## Author contributions

AWR and JM conceived the project and conducted analysis of data. AWR conducted the mechanistic experiments and wrote the first draft of the paper. MC conducted the experimental illustrations of the TBABr additive along with the accompanying characterization. MC and JM revised the manuscript through additions and edits.

## Supplementary Material

SC-013-D2SC01962K-s001

## References

[cit1] Trost B. M. (1983). Science.

[cit2] Davies H. M., Morton D. (2011). Chem. Soc. Rev..

[cit3] Engle K. M., Mei T.-S., Wasa M., Yu J.-Q. (2012). Acc. Chem. Res..

[cit4] Miller D. C., Tarantino K. T., Knowles R. R. (2016). Top. Curr. Chem..

[cit5] Chu J. C., Rovis T. (2016). Nature.

[cit6] Choi G. J., Zhu Q., Miller D. C., Gu C. J., Knowles R. R. (2016). Nature.

[cit7] Thullen S. M., Treacy S. M., Rovis T. (2019). J. Am. Chem. Soc..

[cit8] Morton C. M., Zhu Q., Ripberger H., Troian-Gautier L., Toa Z. S. D., Knowles R. R., Alexanian E. J. (2019). J. Am. Chem. Soc..

[cit9] Xu B., Tambar U. K. (2019). ACS Catal..

[cit10] Yue W. J., Day C. S., Martin R. (2021). J. Am. Chem. Soc..

[cit11] Zhang Z., Zhang X., Nagib D. A. (2019). Chem.

[cit12] Short M. A., Blackburn J. M., Roizen J. L. (2018). Angew. Chem., Int. Ed. Engl..

[cit13] Rand A. W., Yin H., Xu L., Giacoboni J., Martin-Montero R., Romano C., Montgomery J., Martin R. (2020). ACS Catal..

[cit14] Twilton J., Le C., Zhang P., Shaw M. H., Evans R. W., MacMillan D. W. C. (2017). Nat. Rev. Chem..

[cit15] Li G., Ward W. M., Meyer G. J. (2015). J. Am. Chem. Soc..

[cit16] Heitz D. R., Tellis J. C., Molander G. A. (2016). J. Am. Chem. Soc..

[cit17] Kariofillis S. K., Jiang S., Zuranski A. M., Gandhi S. S., Martinez Alvarado J. I., Doyle A. G. (2022). J. Am. Chem. Soc..

[cit18] Barriault L., McCallum T., Rohe S., Morris A. (2018). Angew. Chem., Int. Ed. Engl..

[cit19] Huang L., Rueping M. (2018). Angew. Chem., Int. Ed. Engl..

[cit20] Shields B. J., Kudisch B., Scholes G. D., Doyle A. G. (2018). J. Am. Chem. Soc..

[cit21] Nielsen M. K., Shields B. J., Liu J., Williams M. J., Zacuto M. J., Doyle A. G. (2017). Angew. Chem., Int. Ed. Engl..

[cit22] Tsou T. T., Kochi J. K. (1980). J. Org. Chem..

[cit23] Nicewicz D., Roth H., Romero N. (2015). Synlett.

[cit24] Quinn R. K., Konst Z. A., Michalak S. E., Schmidt Y., Szklarski A. R., Flores A. R., Nam S., Horne D. A., Vanderwal C. D., Alexanian E. J. (2016). J. Am. Chem. Soc..

[cit25] Zhang P., Le C. C., MacMillan D. W. (2016). J. Am. Chem. Soc..

[cit26] Speckmeier E., Fischer T. G., Zeitler K. (2018). J. Am. Chem. Soc..

[cit27] Shu X., Huan L., Huang Q., Huo H. (2020). J. Am. Chem. Soc..

[cit28] Gutierrez O., Tellis J. C., Primer D. N., Molander G. A., Kozlowski M. C. (2015). J. Am. Chem. Soc..

[cit29] Hsieh S. Y., Bode J. W. (2017). ACS Cent. Sci..

[cit30] Yuan M., Song Z., Badir S. O., Molander G. A., Gutierrez O. (2020). J. Am. Chem. Soc..

[cit31] Saveant J. M. (1994). J. Phys. Chem. B.

