# Supplementary material for: Investigations into mechanism and origin of regioselectivity in the metallaphotoredox-catalyzed α-arylation of N-alkylbenzamides
Source: Chem Sci. 2022 Aug 19;13(35):10566–73. doi: 10.1039/d2sc01962k (PMC9473500; doi:10.1039/d2sc01962k)
Supplement: SC-013-D2SC01962K-s001 [file SC-013-D2SC01962K-s001.pdf]

Electronic Supplementary Material (ESI) for Chemical Science.
This journal is © The Royal Society of Chemistry 2022

Supporting Information

Investigations into mechanism and origin of regioselectivity in the metallaphotoredox-catalyzed α-arylation of *N*-alkylbenzamides

Alexander W. Rand, Mo Chen, and John Montgomery**

Department of Chemistry, University of Michigan, Ann Arbor, MI 48109-1055

*jmontg@umich.edu
*alexrand@umich.edu

Table of Contents

I)	General Experimental Details	S2
II)	Starting Material Synthesis	S3
III)	General Procedures for the α-Arylation of Benzamides	S11
IV)	Controls, Additive Screening, and Mechanistic Insights	S17
V)	CV Data	S20
VI)	Stern-Volmer Analysis	S25
VII)	NMR Titration Studies	S28
VIII)	UV-Vis Titration Studies	S29
IX)	NMR Spectra of Starting Material	S32
X)	NMR Spectra of Products	S45
XI)	References	S53

I) General Experimental Details

All reagents were used as received unless otherwise noted. Solvents were purified under nitrogen using a solvent purification system (Innovative Technology, Inc. Model # SPS400-3 and PS-400-3). Ethyl acetate (Sigma-Aldrich, 99.8% anhydrous) was distilled over CaH_2 (Sigma-Aldrich) and then freeze, pumped, thawed three times before storing under N_2. Aryl bromides (Oakwood) were dried on high vacuum or distilled over CaH_2 prior to use. K_3PO_4 (Strem, anhydrous) was finely ground and then heated under vacuum at 100 ºC overnight before transferring to a glove box. Phenylpyridines and bipyridines were synthesized through known procedures.[1] Iridium photocatalysts were synthesized by modified literature procedures for [Ir(dCF$_3$(CF$_3$)ppy)$_2$(4,4'-di-*t*-buBpy)]PF$_6$, [Ir(FCF$_3$(CF$_3$)ppy)$_2$(4,4'-di-*t*-buBpy)]PF$_6$, and [Ir(dF(CF$_3$)ppy)$_2$(4,4'-di-*t*-buBpy)]PF$_6$,[2] [Ir(dF(CF$_3$)ppy)$_2$(5,5'-di-CF$_3$Bpy)]PF$_6$.[1a] Bisoxazoline (BiOx) ligands were synthesized through known procedures.[3]

Analytical thin layer chromatography (TLC) was performed on Kieselgel 60 F254 (250 μm silica gel) glass plates and compounds were visualized with UV light and potassium permanganate or ceric ammonium molybdate stains. Flash column chromatography was performed using Kieselgel 60 (230-400 mesh) silica gel. Eluent mixtures are reported as v:v percentages of the minor constituent in the major constituent. All compounds purified by column chromatography were sufficiently pure for use in further experiments unless otherwise indicated. ^{1}H NMR spectra were collected at 400 MHz on a Varian MR400, at 500 MHz on a Varian Inova 500 or Varian vnmrs 500, or at 700 MHz on a Varian vnmrs 700 instrument. The proton signal of the residual, non-deuterated solvent (δ 7.26 for $CHCl_3$) was used as the internal reference for ^{1}H NMR spectra. ^{13}C NMR spectra were completely heterodecoupled and measured at 100 MHz, 126 MHz or 176 MHz. Chloroform-d (δ 77.00) was used as an internal reference. High resolution mass spectra and LCMS traces were recorded on an Agilent 6545 Q-TOF LS/MS at the University of Michigan Life Sciences Institute. GCMS and GCFID was conducted on an Agilent 7890B GC system with HP-5MS column (30 m x 0.250 mm x 0.25 μm). Infrared spectra were recorded using a Nicolet iS10 FT-IR spectrometer as a neat solid.

II) Synthesis of Starting Materials:

Synthesis of amines:

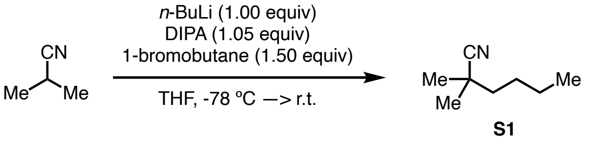

2,2-dimethylhexanenitrile (**S1**)

To a 25 mL oven-dried round-bottom flask equipped with a Teflon-coated magnetic stir bar was added diisopropylamine (1.40 mL, 10.00 mmol, 1.05 equiv) and THF (40 mL, 0.25 M). The reaction was cooled to -78 ºC and *n*BuLi (2.5 M in hexanes) (3.81 mL, 9.52 mmol, 1.00 equiv) was added and then the reaction was stirred for 1 h before adding isobutynitrile (0.85 mL, 9.52 mmol, 1.00 equiv). The reaction was stirred for 1 h before adding 1-bromobutane (1.53 mL, 14.28 mmol, 1.50 equiv). The reaction was then warmed to rt and was stirred overnight. The reaction was cooled to 0 ºC and carefully quenched with sat. NH_4Cl and extracted 3 x 50 mL DCM. The organic layers were dried with 100 mL brine, then Na_2SO_4 and the solvent was removed by rotary evaporation to give the title compound as a yellow oil (1.05 g, 8.00 mmol, 84% yield), which was used without further purification in subsequent steps.[4]

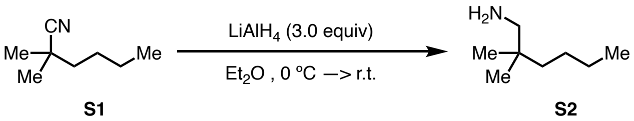

2,2-dimethylhexan-1-amine (**S2**)

To a 100 mL oven-dried round-bottom flask equipped with a Teflon-coated magnetic stir bar was added LAH (0.95 g, 25.10 mmol, 3.00 equiv) and Et_2O (40 mL, 0.21 M). The suspension was cooled to 0 ºC and nitrile (1.05 g, 8.39 mmol, 1.00 equiv) was added slowly as a solution in 10 mL Et_2O. The reaction was then warmed to rt and stirred for 3 h. The reaction was then cooled to 0 ºC and carefully quenched with 1 M NaOH until a white precipitate formed. The slurry was then filtered through celite and the precipitate washed with Et_2O. The organics were then concentrated by rotary evaporation to give the title compound as a clear oil (867 mg, 6.71 mmol, 80% yield), which was used without further purification in subsequent steps.[4]

General procedures for the synthesis of benzamides:

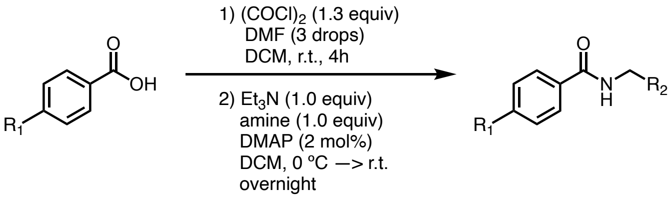

To a 100 mL oven-dried round-bottom flask equipped with a Teflon-coated magnetic stir bar was added benzoic acid (5.00 mmol, 1.00 equiv). The flask was backfilled with N_2 three times before adding DCM (25 mL, 0.2 M) and cooling to 0 ºC in an ice bath. Oxalyl chloride (0.55 mL, 6.50 mmol, 1.30 equiv) was then added followed by DMF (3-5 drops). The reaction was then allowed

to warm to rt and stirred for 4 h. The reaction was then concentrated under reduced pressure using rotary evaporation. The residue was then taken up in fresh DCM (25 ml, 0.2 M) and cooled to 0 ºC. Et_3N (0.70 mL, 5.00 mmol, 1.00 equiv) was added dropwise followed by amine (5.00 mmol, 1.00 equiv) and DMAP (12 mg, 0.1 mmol, 0.02 equiv). The reaction was then allowed to warm to rt and stirred for 16 h. The reaction was quenched with quenched with 10 mL H_2O and 25 mL DCM and the organics were washed 2 x 25 mL 1M HCl, 2 x 25 mL 1M NaOH, dried with 25 mL brine, and then over Na_2SO_4. The solvent was removed by rotary evaporation and the crude reaction mixture was purified by silica gel chromatography.[5]

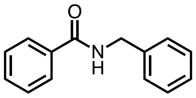

1a

N-benzylbenzamide (**1a**)

To a 100 mL oven-dried round-bottom flask equipped with a Teflon-coated magnetic stir bar under N_2 was added benzoylchloride (0.5 mL, 4.30 mmol, 1.00 equiv) and DCM (20 ml, 0.2 M). The flask was cooled to 0 ºC and Et_3N (0.60 mL, 4.30 mmol, 1.00 equiv) was added dropwise followed by benzylamine (0.47 mL, 4.30 mmol, 1.00 equiv) and DMAP (12 mg, 0.10 mmol, 0.02 equiv). The reaction was then allowed to warm to rt and stirred for 16 h. The reaction was quenched with quenched with 10 mL H_2O and 25 mL DCM and the organics were washed 2 x 25 mL 1M HCl, 2 x 25 mL 1M NaOH, dried with 25 mL brine, and then over Na_2SO_4. The solvent was removed by rotary evaporation to give the title compound as a white powder (845 mg, 4.00 mmol, 93% yield).

Rf: 0.15 (90:10 hexanes/EtOAc)

^{1}H NMR (401 MHz, Chloroform-*d*) δ 7.83 – 7.77 (m, 2H), 7.55 – 7.48 (m, 1H), 7.43 (dd, *J* = 8.2, 6.9 Hz, 2H), 7.36 (d, *J* = 4.3 Hz, 4H), 7.31 (p, *J* = 4.6 Hz, 1H), 6.40 (s, 1H), 4.66 (d, *J* = 5.6 Hz, 2H).

^{13}C NMR (126 MHz, Chloroform-*d*) δ 167.51, 138.36, 134.45, 131.56, 128.79, 128.60, 127.91, 127.58, 127.10, 44.11.

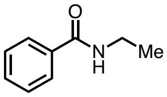

1b

N-ethylbenzamide (**1b**)

To an oven-dried 100-mL round-bottom flask containing a Teflon-coated magnetic stir bar and equipped to a reflux condenser was added benzamide (500 mg, 4.10 mmol, 1.00 equiv). The flask was backfilled with N_2 three times before adding cyclopentyl ether (20 mL, 0.2 M) and triethylphosphate (2.10 mL, 12.40 mmol, 3.00 equiv). *n*BuLi (2.5 M in hexanes) (3.0 mL, 7.40 mmol, 1.80 equiv) was added dropwise and the reaction was heated to 115 ºC for 24 h. The reaction was carefully quenched with 20 mL brine and then extracted 3 x 30 mL EtOAc. The organics were then dried over Na_2SO_4 and the solvent was removed by rotary evaporation. Purification by silica gel chromatography (70:30 hexanes/EtOAc to 1:1 hexanes/EtOAc) gave the title compound as a white sticky solid (274 mg, 1.84 mmol, 45% yield). The spectral data matched that previously reported in the literature.[6]

Rf: 0.10 (1:1 hexanes/EtOAc)

^{1}H NMR (401 MHz, Chloroform-*d*) δ 7.76 (d, *J* = 7.1 Hz, 2H), 7.54 – 7.35 (m, 3H), 6.11 (s, 1H), 3.51 (qd, *J* = 7.3, 5.6 Hz, 2H), 1.26 (t, *J* = 7.3 Hz, 2H).

^{13}C NMR (126 MHz, Chloroform-*d*) δ 167.56, 134.62, 128.14, 126.90, 34.74, 14.62.

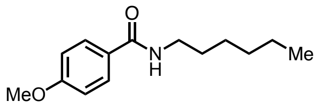

1d

N-hexyl-4-methoxybenzamide (**1d**)

The general procedure for benzamide synthesis coupling was followed using 4-methoxybenzoic acid (600 mg, 3.94 mmol, 1.00 equiv), oxalyl chloride (0.43 mL, 5.13 mmol, 1.30 equiv), DCM (20 mL, 0.2 M), DMF (3 drops), Et_3N (0.55 mL, 3.94 mmol, 1.00 equiv), *n*-hexylamine (0.52 mL, 3.94 mmol, 1.00 equiv) and DMAP (10 mg, 0.10 mmol, 0.02 equiv). Purification by aqueous work-up gave the title compound as a white solid (870 mg, 3.70 mmol, 94% yield over two steps). The spectral data matches that previously reported in the literature.[7]

Rf: 0.20 (70:30 hexanes/EtOAc)

^{1}H NMR (400 MHz, Chloroform-*d*) δ 7.72 (d, *J* = 8.7 Hz, 2H), 6.92 (d, *J* = 8.5 Hz, 2H), 6.02 (s, 1H), 3.84 (s, 3H), 3.43 (q, *J* = 6.7 Hz, 2H), 1.60 (p, *J* = 7.2 Hz, 2H), 1.53 – 1.19 (m, 6H), 1.06 – 0.79 (t, *J* = 6.5 Hz, 3H).

^{13}C NMR (126 MHz, Chloroform-*d*) δ 167.12, 162.04, 128.73, 127.26, 113.70, 55.43, 40.15, 31.62, 29.79, 26.78, 22.65, 14.10.

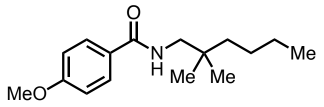

1f

N-(2,2-dimethylhexyl)-4-methoxybenzamide (**1f**)

The general procedure for benzamide synthesis coupling was followed using 4-methoxybenzoic acid (1.40 g, 9.23 mmol, 1.10 equiv), oxalyl chloride (1.01 mL, 12.00 mmol, 1.43 equiv), DCM (37 mL, 0.2 M), DMF (3 drops), Et_3N (1.17 mL, 8.39 mmol, 1.0 equiv), **23** (8.39 mmol, 1.00 equiv) and DMAP (15 mg, 0.15 mmol, 0.02 equiv). Purification by aqueous work-up followed by silica gel chromatography (80:20 to 70:30 hexanes/EtOAc) gave the title compound as a white crystalline solid (1.954 mg, 6.71 mmol, 80% yield over 4 steps) that was one spot by TLC and GCMS. NMR showed an equilibrium of rotamers.

Rf: 0.30 (70:30 hexanes/EtOAc)

^{1}H NMR (401 MHz, Chloroform-*d*) δ 8.10 (d, *J* = 8.5 Hz, 1H), 7.73 (d, *J* = 8.4 Hz, 2H), 6.98 (d, *J* = 8.7 Hz, 1H), 6.93 (d, *J* = 8.5 Hz, 2H), 6.01 (s, 1H), 3.90 (s, 1H), 3.85 (s, 3H), 3.28 (d, *J* = 6.2 Hz, 2H), 1.39 – 1.10 (m, 6H), 0.92 (d, *J* = 11.7 Hz, 9H).

^{13}**C NMR** (126 MHz, Chloroform-*d*) δ 167.21, 162.05, 132.85, 128.65, 127.44, 114.19, 113.75, 55.64, 55.42, 49.59, 39.91, 34.54, 26.20, 25.11, 23.62, 14.18.

HRMS: (ESI) (m/z): [M+H] calculated for $C_{16}H_{25}NO_2$, 264.1963, found 264.1988.

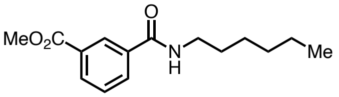

1g

methyl 3-(hexylcarbamoyl)benzoate (**1g**)

The general procedure for benzamide synthesis coupling was followed using 3-(methoxycarbonyl)benzoic acid (900 mg, 5.00 mmol, 1.00 equiv), oxalyl chloride (0.55 mL, 6.50 mmol, 1.3 equiv), DCM (25 mL, 0.2 M), DMF (3 drops), Et_3N (0.70 mL, 5.00 mmol, 1.0 equiv), *n*-hexylamine (0.66 mL, 5.00 mmol, 1.0 equiv) and DMAP (12 mg, 0.10 mmol, 0.02 equiv). Purification by silica gel chromatography (70:30 hexanes/EtOAc to 1:1 hexanes/EtOAc) gave the title compound as a white solid (1.119 g, 4.25 mmol, 85% yield over two steps).

Rf: 0.10 (70:30 hexanes/EtOAc)

^{1}**H NMR** (700 MHz, Chloroform-*d*) δ 8.34 (s, 1H), 8.10 (d, *J* = 7.8 Hz, 1H), 8.00 (d, *J* = 7.8 Hz, 1H), 7.46 (t, *J* = 7.7 Hz, 1H), 6.57 – 6.34 (m, 1H), 3.89 (s, 3H), 3.42 (d, *J* = 6.9 Hz, 2H), 1.58 (q, *J* = 7.5 Hz, 2H), 1.40 – 1.15 (m, 6H), 0.85 (t, *J* = 6.5 Hz, 3H).

^{13}**C NMR** (176 MHz, Chloroform-*d*) δ 166.56, 166.47, 135.24, 132.25, 131.89, 130.41, 128.87, 127.60, 52.42, 40.35, 31.58, 29.67, 26.76, 22.64, 14.11.

HRMS: (ESI) (m/z): [M+H] calculated for $C_{15}H_{21}NO_3$, 264.1600, found 264.1609.

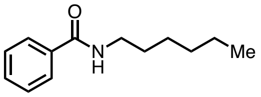

1h

N-hexylbenzamide (**1h**)

To a 100 mL oven-dried round-bottom flask equipped with a Teflon-coated magnetic stir bar under N_2 was added benzoylchloride (1 mL, 8.60 mmol, 1.0 equiv) and DCM (25 ml, 0.2 M). The flask was cooled to 0 ºC and Et_3N (1.20 mL, 8.60 mmol, 1.0 equiv) was added dropwise followed by *n*-hexylamine (1.14 mL, 8.60 mmol, 1.0 equiv) and DMAP (20 mg, 0.17 mmol, 0.02 equiv). The reaction was then allowed to warm to room temperature and stirred overnight. The reaction was quenched with quenched with 10 ml H_2O and 25 mL DCM and the organics were washed 2 x 25 mL 1M HCl, 2 x 25 mL 1M NaOH, dried with 25 mL brine, and then over Na_2SO_4. The solvent was removed by rotary evaporation and the crude reaction mixture was purified by silica gel chromatography (100% hexanes to 80:20 hexanes/EtOAc) to give the title compound as a white solid (1.628 g, 7.91 mmol, 92% yield). The spectral data matches that previously reported in the literature.[7]

Rf: 0.15 (80:20 hexanes/EtOAc)

^{1}H NMR (400 MHz, Chloroform-*d*) δ 7.76 (d, *J* = 7.3 Hz, 2H), 7.61 – 7.38 (m, 3H), 6.09 (s, 1H), 3.46 (q, *J* = 6.7 Hz, 2H), 1.61 (h, *J* = 7.2 Hz, 2H), 1.48 – 1.16 (m, 6H), 0.95 – 0.84 (t, *J* = 6.5 Hz, 3H).

^{13}C NMR (126 MHz, Chloroform-*d*) δ 167.64, 134.89, 131.17, 128.40, 126.97, 40.17, 31.54, 29.64, 26.71, 22.58, 14.03.

Synthesis of *i*-PrBiOxNiCl$_2$:

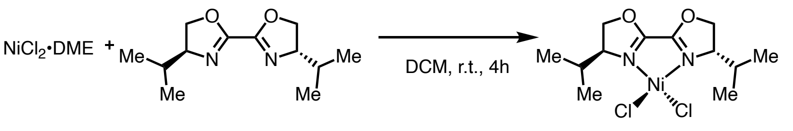

To a 50 mL oven-dried round-bottom flask with a Teflon-coated magnetic stir bar in a glovebox was added $NiCl_2$•DME (91.6 mg, 0.42 mmol, 1.0 equiv) and *i*-PrBiOx (112 mg, 0.5 mmol, 1.2 equiv) and DCM (15 mL, 0.03 M). The mixture turned homogenous and reaction was stirred for 4 hours before stripping the solvent by high vacuum. The brownish residue was then taken up in 3 mL DCM and Et_2O was added to precipitate a white solid, which was filtered off. Pentanes were then added to the mother liquor resulting in further precipitation. This precipitate was then washed with pentanes and Et_2O and dried under high vacuum for 1 hour to give an off-white powder (94.8 mg, 0.267 mmol, 64%). The powder was dissolved in C_6D_6 and was observed to be paramagnetic by NMR.[8]

^{1}H NMR (401 MHz, C_6D_6) δ 23.03 ppm (br, 2H) 19.02 ppm, (br, 2H) 2.63 ppm (br, 3H), 2.00 (br, 4H), 1.70ppm (br, 6H), and 0.87 ppm (br, 1H).

UV-Vis (EtOAc, ambient temperature): λ_{max} = 272 nm (4,100 $M^{-1}cm^{-1}$) and 228 nm (3,600 $M^{-1}cm^{-1}$)

IR (neat, ambient temperature): 1651 cm^{-1} (C=N)

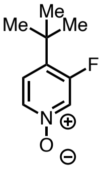

S3

4-(tert-butyl)-3-fluoropyridine 1-oxide (S3)

To an oven-dried round bottom flask under an N_2 atmosphere with a Teflon stir bar was added 3-fluoropyridine (0.26 mL, 3.0 mmol, 1.0 equiv) and DCM (18 mL). TIPSOTf (0.87 mL, 3.2 mmol, 1.05 equiv) was added dropwise and the reaction was stirred for 15 minutes at rt. Cool solution to -78 ºC and 1,4-dioxane (1.14 mL) was added. Then, *t*-BuMgCl (1.7 M in THF) (7 mL, 12.0 mmol, 4.0 equiv) was added and the reaction was warmed slowly to rt and stirred 16 h. Reaction was quenched with H_2O and sat. $NaHCO_3$ and then extracted 3x25 mL DCM. The organic layers were dried with 50 mL brine, then over Na_2SO_4. Organics were concentrated and the crude oil was carried on without further purification.

The crude oil was then added to a 25 mL round bottom flask under an N_2 atmosphere with a Teflon stir bar and reflux condenser and dissolved in decaline (6 mL) and S_8 (110 mg, 3.3 mmol, 1.1 equiv) was added, and the reaction was placed under an N_2 atmosphere. The solution was stirred at 190 ºC for 2 h. Cool reaction and load directly on to silica gel chromatography (2:1 pentane/Et_2O, Rf: 0.15). Fractions were carefully concentrated via rotary evaporation (product is volatile!) and the clear oil was carried on directly to the next step.

Crude oil from the previous step was added to a 25 mL round bottom flask under an N_2 atmosphere with a Teflon stir bar and DCM (7.5 mL) was added. *m*-CPBA (1.00g, 3.0 mmol, 1.0 equiv) was added in a single portion and the reaction was stirred for 16 h. The reaction was partially concentrated (avoid concentrating to dryness since *m*-CPBA should not be completely dry) and then loaded on silica gel chromatography (100% EtOAc, then 100% DCM to 90:10 DCM/MeOH) to give 396 mg (78% yield over 3 steps) of an off-white powder.

^{1}H NMR (401 MHz, Methanol-d_4) δ 8.34 (dd, *J* = 7.0, 2.0 Hz, 1H), 8.17 – 8.08 (m, 1H), 7.50 (dd, *J* = 9.5, 6.8 Hz, 1H), 1.40 (s, 9H).

^{13}C NMR (126 MHz, Methanol-d_4) δ 136.68 (d, *J* = 3.0 Hz), 130.80, 130.66 – 129.77 (m), 125.71 (d, *J* = 7.2 Hz), 35.59, 29.47 (d, *J* = 3.1 Hz).

^{19}F NMR (377 MHz, Methanol-d_4) δ -118.66.

HRMS: (ESI) (m/z): [M+H] calculated for $C_9H_{12}FNO$, 170.0981, found 170.0976.

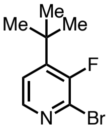

S4

2-bromo-4-(tert-butyl)-3-fluoropyridine (S4)

To an oven-dried 1-dram vial under an N_2 atmosphere with a Teflon stir bar was added **S3** (194 mg, 1.15 mmol, 1.0 equiv), triethylamine (0.32 mL, 2.3 mmol, 2.0 equiv), and dibromomethane (2 mL). The reaction was then cooled to -50 ºC and oxalyl bromide (0.22 mL, 2.3 mmol, 2.0 equiv) was added dropwise and the reaction was stirred for 30 minutes. The reaction was then quenched with MeOH (0.5 mL) and warmed to rt. The organics were washed with sat. NH_4Cl (3 mL), H_2O (3 mL), then dried over $MgSO_4$ and the organics were concentrated. Purification by silica gel chromatography (1:1 hexanes/DCM) gave the title compound as a clear oil (238 mg, 89% yield).

Rf: 0.20 (1:1 hexanes/DCM)

^{1}H NMR (401 MHz, Chloroform-*d*) δ 8.09 (d, J = 5.0 Hz, 1H), 7.18 (t, J = 5.4 Hz, 1H), 1.39 (s, 9H).

^{19}F NMR (377 MHz, Chloroform-*d*) δ -109.27.

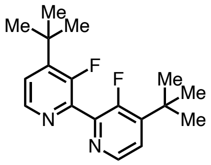

S5

4,4'-di-tert-butyl-3,3'-difluoro-2,2'-bipyridine (S5)

To a round-bottom with equipped with a Teflon-coated magnetic stir bar and equipped to a reflux condenser was added $Pd(OAc)_2$ (5.6 mg, 0.025 mmol, 2.5 mol%), indium powder (57 mg, 0.6 mmol, 0.5 equiv), **S4** (232 mg, 1.0 mmol, 1.0 equiv), and LiCl (64 mg, 1.5 mmol, 1.5 equiv). The flask was backfilled with N_2 three times before adding DMF (2.3 mL, 0.44 M). The reaction was heated to 100 ºC for 40 minutes. The flask was cooled to rt and the mixture diluted with 25 mL H_2O and extracted 3 x 30 mL Et_2O, the organics were washed 2 x 50 mL H_2O, then dried with 50 mL brine and then $MgSO_4$ before removing the solvent by rotary evaporation. The crude reaction mixture was purified by silica gel chromatography (90:10 hexanes/EtOAc to 1:1 hexanes/EtOAc) to give a tan solid (53 mg, 35% yield).

^{1}H NMR (400 MHz, Chloroform-*d*) δ 8.46 (d, *J* = 5.0 Hz, 1H), 7.50 – 7.28 (m, 1H), 1.42 (s, 9H).

^{13}C NMR (126 MHz, Chloroform-*d*) δ 158.35, 146.62, 145.73 (t, *J* = 2.9 Hz), 122.63, 34.80, 29.45.

^{19}F NMR (376 MHz, Chloroform-*d*) δ -121.46.

HRMS: (ESI) (m/z): [M+H] calculated for $C_{18}H_{22}F_2N_2$, 305.1829, found 305.1827.

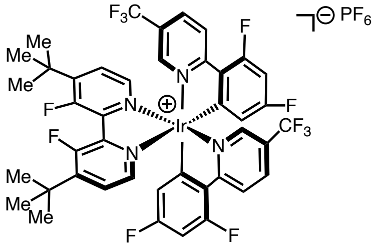

S6

[Ir(dF(CF$_3$)ppy)2(4,4'-di-tert-butyl-3,3'-difluoro-2,2'-bipyridine)]PF$_6$ (S6)

Using the general procedure for iridium photocatalyst synthesis, cationic iridium acetonitrile adduct (168 mg, 0.18 mmol, 1.0 equiv) and **S5** (56 mg, 0.189 mmol, 1.05 equiv) were added to a Schlenk flask with a Teflon stir bar. DCM (1.8 mL) and EtOH (0.6 mL) were added and the reaction was stirred at 50 ºC for 36 h. After the reaction was complete, the solution was filtered through Celite and concentrated. Purification by silica gel chromatography (100% DCM to 95:5 DCM/acetone) gave the title compound as a yellow powder (193 mg, 93% yield).

^{1}H NMR (401 MHz, Acetone-*d*$_6$) δ 8.61 (dd, *J* = 8.9, 2.6 Hz, 1H), 8.42 (dd, *J* = 8.8, 2.1 Hz, 1H), 8.15 (dd, *J* = 5.6, 1.2 Hz, 1H), 7.87 (dt, *J* = 6.2, 3.3 Hz, 1H), 7.72 (d, *J* = 2.0 Hz, 1H), 6.86 (ddd, *J* = 12.9, 9.3, 2.3 Hz, 1H), 5.94 (dd, *J* = 8.5, 2.3 Hz, 1H), 1.49 (s, 9H).

^{13}**C NMR** (176 MHz, Acetone-d_6) δ 168.40, 165.98, 164.51, 163.93 (d, J = 13.3 Hz), 162.48, 161.91 – 160.37 (m), 159.11, 153.99, 152.79, 149.12, 147.22, 144.48, 138.31, 129.23, 127.81, 126.35, 126.15, 124.77 (d, J = 20.9 Hz), 123.81, 122.27, 115.54 (d, J = 18.0 Hz), 102.07 – 98.38 (m), 36.46, 29.14.

^{19}**F NMR** (377 MHz, Acetone-d_6) δ -63.48 (d, J = 2.4 Hz), -71.72, -73.60, -104.46, -104.68 (d, J = 12.0 Hz), -106.68 – -108.71 (m).

HRMS: (ESI) (m/z): [M+H] calculated for $C_{42}H_{32}F_{12}IrN_4$, 1013.2065, found 1013.2069.

III) General Procedures for the α-Arylation of Benzamides:

To an oven-dried 1 dram vial equipped with a Teflon-coated magnetic stir bar in a N_2 filled glovebox was added [Ir(dF(CF_3)ppy)$_2$(4,4'-di-*t*-buBpy)]PF_6 (PC1) (4.4 mg, 0.004 mmol, 0.02 equiv), $NiCl_2$•DME (2.2 mg, 0.01 mmol, 0.05 equiv), bisoxazoline (BiOx) (1.4 mg, 0.01 mmol, 0.05 equiv), K_3PO_4 (85 mg, 0.4 mmol, 2.0 equiv), and benzamide (0.2 mmol, 1.00 equiv) were combined and suspended in 1.0 mL of dry, degassed EtOAc at rt. The mixture was stirred for 10 minutes before adding aryl bromide (1.5 mmol, 7.50 equiv). The vial was sealed with a Teflon cap before removing from the glovebox. The reaction was stirred at 900 rpm for 16 h in a recrystallization dish filled with water (for cooling) and irradiated with a 34 W blue LED lamp placed 1 cm away. Upon completion, the reaction mixture was quenched with 1 mL EtOAc and run through a silica gel plug with 5 mL EtOAc. The solvent was removed by rotary evaporation and the crude reaction mixture was purified by silica gel chromatography.

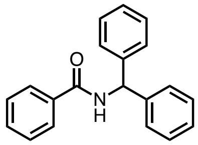

2a

N-benzhydrylbenzamide (**2a**)

The general procedure for the α-arylation of benzamides was followed using **1a** (42 mg, 0.200 mmol, 1.00 equiv), PC1 (2.2 mg, 0.002 mmol, 0.01 equiv), $NiCl_2$•DME (4.4 mg, 0.020 mmol, 0.10 equiv), BiOx (2.8 mg, 0.020 mmol, 0.10 equiv), K_3PO_4 (85 mg, 0.400 mmol, 2.00 equiv), and bromobenzene (156 uL, 1.500 mmol, 7.50 equiv) in 1.0 mL DMAc. Purification by silica gel chromatography (100% hexanes to 90:10 hexanes/EtOAc) gave the title compound as a white solid (24.1 mg, 0.068 mmol, 18% yield). The spectral data matches that previously reported in the literature.[9]

Rf: 0.25 (80:20 hexanes/EtOAc)

^{1}H NMR (700 MHz, Chloroform-*d*) δ 7.88 – 7.79 (m, 2H), 7.53 – 7.48 (m, 1H), 7.44 (td, *J* = 7.8, 1.9 Hz, 2H), 7.39 – 7.34 (m, 5H), 7.34 – 7.27 (m, 5H), 6.73 (d, *J* = 7.9 Hz, 1H), 6.46 (d, *J* = 7.8 Hz, 1H).

^{13}C NMR (176 MHz, Chloroform-*d*) δ 166.61, 141.56, 134.34, 131.82, 128.87, 128.75, 127.70, 127.63, 127.18, 57.57.

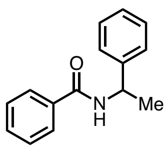

2b

N-(1-phenylethyl)benzamide (**2b**)

The general procedure for the α-arylation of benzamides was followed using **1b** (32 mg, 0.200 mmol, 1.00 equiv), [Ir(FCF$_3$(CF$_3$)ppy)$_2$(4,4'-di-*t*-buBpy)]PF_6 (5.1 mg, 0.004 mmol, 0.02 equiv), $NiCl_2$•DME (2.2 mg, 0.010 mmol, 0.05 equiv), BiOx (1.4 mg, 0.010 mmol, 0.05 equiv), K_3PO_4 (85 mg, 0.400 mmol, 2.00 equiv), and bromobenzene (156 uL, 1.500 mmol, 7.50 equiv) in 1 mL

EtOAc/DMAc (9:1). Purification by silica gel chromatography (80:20 hexanes/EtOAc) gave the title compound as a white solid (18.9 mg, 0.084 mmol, 42% yield). The spectral data matches that previously reported in the literature.[10]

Rf: 0.40 (70:30 hexanes/EtOAc)

^{1}H NMR (700 MHz, Chloroform-*d*) δ 7.79 – 7.75 (m, 2H), 7.48 (t, *J* = 7.5 Hz, 1H), 7.40 (dd, *J* = 14.2, 7.5 Hz, 3H), 7.35 (t, *J* = 7.6 Hz, 3H), 7.28 (d, *J* = 7.5 Hz, 1H), 6.48 (d, *J* = 11.1 Hz, 1H), 5.34 (p, *J* = 7.1 Hz, 1H), 1.60 (d, *J* = 6.9 Hz, 3H).

^{13}C NMR (176 MHz, Chloroform-*d*) δ 166.69, 143.25, 134.67, 131.55, 128.83, 128.63, 127.53, 127.05, 126.36, 49.31, 21.84.

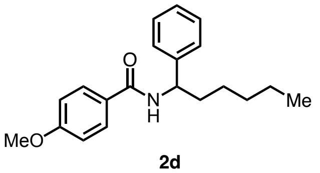

2d

4-methoxy-*N*-(1-phenylhexyl)benzamide (**2d**)

The general procedure for the α-arylation of benzamides was followed using **1d** (96 mg, 0.400 mmol, 2.00 equiv), PC1 (4.4 mg, 0.004 mmol, 0.02 equiv), $NiCl_2$•DME (2.2 mg, 0.010 mmol, 0.05 equiv), BiOx (1.4 mg, 0.010 mmol, 0.05 equiv), K_3PO_4 (85 mg, 0.400 mmol, 2.00 equiv), TBABr (64 mg, 0.200 mmol, 1.00 equiv), and bromobenzene (20.8 uL, 0.200 mmol, 1 equiv) in 1.0 mL EtOAc. Analysis by GCFID using tridecane as an internal standard showed 73% yield of the desired product. Purification by silica gel chromatography (70:30 hexanes/EtOAc) gave the title compound as a white solid (39.6 mg, 0.127 mmol, 64% yield). The spectral data matches that previously reported in the literature.[11]

Rf: 0.35 (70:30 hexanes/EtOAc)

^{1}H NMR (500 MHz, Chloroform-*d*) δ 7.73 (d, *J* = 8.5 Hz, 2H), 7.49 – 7.14 (m, 5H), 6.91 (d, *J* = 8.6 Hz, 2H), 6.21 (d, *J* = 8.1 Hz, 1H), 5.15 (q, *J* = 7.6 Hz, 1H), 3.84 (s, 3H), 2.01 – 1.76 (m, 2H), 1.50 – 1.20 (m, 6H), 0.86 (t, *J* = 6.8 Hz, 3H).

^{13}C NMR (126 MHz, Chloroform-*d*) δ 166.27, 162.28, 142.74, 128.83 (two overlapping peaks), 127.46, 127.12, 126.78, 113.87, 55.58, 53.96, 36.45, 31.75, 26.13, 22.65, 14.16.

HRMS: (ESI) (m/z): [M+H] calculated for $C_{20}H_{25}NO_2$, 312.1963, found 312.1994.

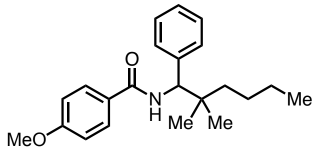

2f

N-(2,2-dimethyl-1-phenylhexyl)-4-methoxybenzamide (**2f**)

The general procedure for the α-arylation of benzamides was followed **1f** (50 mg, 0.200 mmol, 1.00 equiv), PC1 (4.5 mg, 0.004 mmol, 0.02 equiv), $NiCl_2$•DME (2.2 mg, 0.010 mmol, 0.05 equiv), BiOx (1.4 mg, 0.010 mmol, 0.05 equiv), K_3PO_4 (85 mg, 0.400 mmol, 2.00 equiv), TBABr (64 mg, 0.200 mmol, 1.00 equiv), and bromobenzene (156 uL, 1.500 mmol, 7.50 equiv) in 1 mL EtOAc. Purification by silica gel chromatography (90:10 to 70:30 hexanes/EtOAc) gave the title compound as a white solid (3.8 mg, 0.011 mmol, 6% yield) and recovered **1f** (38.6 mg, 0.154 mmol, 77% recovery).

Rf: 0.25 (70:30 hexanes/EtOAc)

^{1}H NMR (700 MHz, Chloroform-*d*) δ 7.75 – 7.68 (m, 2H), 7.42 (d, *J* = 8.3 Hz, 1H), 7.32 – 7.18 (m, 5H), 6.93 (d, *J* = 8.5 Hz, 2H), 6.63 (d, *J* = 8.4 Hz, 1H), 6.59 (d, *J* = 9.1 Hz, 1H), 5.05 (d, *J* = 9.1 Hz, 1H), 3.84 (s, 3H), 3.72 (s, 1H), 1.37 – 1.22 (m, 4H), 1.00 – 0.85 (m, 9H).

^{13}C NMR (176 MHz, Chloroform-*d*) δ 166.21, 162.23, 140.30, 131.14, 128.73, 128.40, 127.94, 127.47, 127.12, 113.93, 113.56, 60.85, 55.56, 55.45, 41.14, 39.62, 37.55, 29.85, 26.23, 26.20, 24.22, 23.89, 23.68, 14.28.

HRMS: (ESI) (m/z): [M+H] calculated for $C_{22}H_{29}NO_2$, 340.2276, found 340.2329.

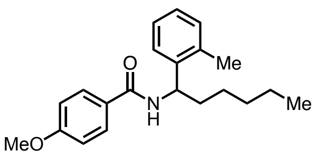

2g

4-methoxy-*N*-(1-(*o*-tolyl)hexyl)benzamide (**2g**)

The general procedure for the α-arylation of benzamides was followed using **1d** (94 mg, 0.400 mmol, 2.00 equiv), PC1 (4.4 mg, 0.004 mmol, 0.02 equiv), $NiCl_2$•DME (2.2 mg, 0.010 mmol, 0.05 equiv), BiOx (1.4 mg, 0.010 mmol, 0.05 equiv), K_3PO_4 (85 mg, 0.400 mmol, 2.00 equiv), TBABr (64 mg, 0.200 mmol, 1.00 equiv), and 2-methylbromobenzene (24 uL, 0.200 mmol, 1.00 equiv) in 1 mL EtOAc. Analysis by ^{1}H NMR using dibromomethane as an internal standard showed 54% yield of the desired product. Purification by silica gel chromatography (90:10 to 80:20 hexanes/EtOAc) gave the title compound as a white solid (27.0 mg, 0.083 mmol, 41% yield).

Rf: 0.40 (70:30 hexanes/EtOAc)

^{1}H NMR (400 MHz, Chloroform-*d*) δ 7.70 (d, *J* = 9.0 Hz, 2H), 7.28 (d, *J* = 7.4 Hz, 2H), 7.23 – 7.10 (m, 3H), 6.88 (d, *J* = 8.8 Hz, 2H), 6.17 (d, *J* = 8.1 Hz, 1H), 5.36 (q, *J* = 7.6 Hz, 1H), 3.81 (s, 3H), 2.43 (s, 3H), 1.84 (tdd, *J* = 15.3, 11.3, 5.7 Hz, 1H), 1.28 (p, *J* = 11.8 Hz, 6H), 0.84 (t, 6.6 Hz, 3H).

^{13}C NMR (100 MHz, Chloroform-*d*) δ 166.12, 162.24, 140.89, 136.44, 130.89, 128.79, 127.26, 127.03, 126.40, 125.04, 113.84, 55.54, 50.03, 36.20, 31.84, 26.20, 22.67, 19.63, 14.18.

HRMS: (ESI) (m/z): [M+H] calculated for $C_{21}H_{27}NO_2$, 326.2120, found 326.2125.

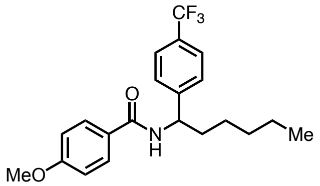

2h

4-methoxy-*N*-(1-(4-(trifluoromethyl)phenyl)hexyl)benzamide (**2h**)

The general procedure for the α-arylation of benzamides was followed using **1d** (47 mg, 0.200 mmol, 2.00 equiv), PC1 (2.2mg, 0.002 mmol, 0.02 equiv), $NiCl_2$•DME (1.1 mg, 0.005 mmol, 0.05 equiv), BiOx (0.7 mg, 0.005 mmol, 0.05 equiv), K_3PO_4 (47 mg, 0.200 mmol, 2.00 equiv), TBABr (32 mg, 0.100 mmol, 1.00 equiv), and 4-bromobenzotrifluoride (14 uL, 0.100 mmol, 1.00 equiv) in 0.5 mL EtOAc. Analysis by ^1H NMR using dibromomethane as an internal standard showed 71% yield of the desired product. Purification by silica gel chromatography (90:10 to 80:20 hexanes/EtOAc) gave the title compound as a white solid (24.1 mg, 0.064 mmol, 64% yield). The spectral data matches that previously reported in the literature.[11]

Rf: 0.40 (70:30 hexanes/EtOAc)

^{1}H NMR (500 MHz, Chloroform-*d*) δ 7.85 (d, J = 8.0 Hz, 2H), 7.77 – 7.57 (m, 2H), 7.28 (q, J = 4.5 Hz, 1H), 6.45 (d, J = 7.9 Hz, 1H), 5.15 (q, J = 7.7 Hz, 1H), 2.04 – 1.82 (m, 2H), 1.44 – 1.14 (m, 8H), 0.86 (t, J = 6.7 Hz, 3H).

^{13}C NMR (126 MHz, Chloroform-*d*) δ 165.58, 142.14, 138.09, 133.41, 133.15, 128.93, 128.23, 127.71, 127.54, 126.76, 125.71 (q, J = 3.6 Hz), 123.78 (q, J = 272.3 Hz), 54.40, 36.26, 31.68, 26.13, 22.63, 14.13.

^{19}F NMR (377 MHz, Chloroform-*d*) δ -63.03.

HRMS: (ESI) (m/z): [M+H] calculated for $C_{21}H_{24}F3NO_2$, 380.1837, found 380.1893.

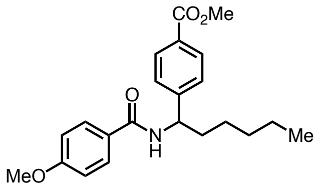

2i

methyl 4-(1-(4-methoxybenzamido)hexyl)benzoate (**2i**)

The general procedure for the α-arylation of benzamides was followed using 1d (47 mg, 0.200 mmol, 2.00 equiv), PC1 (2.2 mg, 0.004 mmol, 0.02 equiv), $NiCl_2$ •DME (1.1 mg, 0.005 mmol, 0.05 equiv), BiOx (0.7 mg, 0.005 mmol, 0.05 equiv), K_3PO_4 (44 mg, 0.200 mmol, 2.00 equiv), TBABr (32 mg, 0.100 mmol, 1.00 equiv), and methyl-3-bromobenzoate (22 mg, 0.1 mmol, 1.00 equiv) in 0.5 mL EtOAc. Analysis by ^1H NMR using dibromomethane as an internal standard showed 75% yield of the desired product. Purification by silica gel chromatography (100% hexanes to 90:10 hexanes/Acetone) gave the title compound as a white solid (24.7 mg, 0.067 mmol, 67% yield).

Rf: 0.10 (10:90 Hexane/Acetone)

^{1}H NMR (600 MHz, $CDCl_3$-*d*) δ 7.99 (d, *J* = 8.4 Hz, 2H), 7.73 (d, *J* = 8.8 Hz, 2H), 7.40 (d, *J* = 8.4 Hz, 2H), 6.90 (d, *J* = 8.8 Hz, 2H), 6.35 (d, *J* = 7.9 Hz, 1H), 5.16 (q, *J* = 7.5 Hz, 1H), 3.89 (s, 3H), 3.83 (s, 3H), 1.92 – 1.81 (m, 2H), 1.43 – 1.22 (m, 6H), 0.85 (t, *J* = 6.8 Hz, 3H).

^{13}C NMR (151 MHz, $CDCl_3$-*d*) δ 166.86, 166.30, 162.26, 148.00, 129.99, 129.09, 128.73, 126.60, 126.55, 113.76, 55.41, 53.68, 52.07, 36.24, 31.50, 25.90, 22.46, 13.97.

HRMS: (ESI) (m/z): [M+H] calculated for $C_{22}H_{27}NO_4$, 370.2018, found 370.1994.

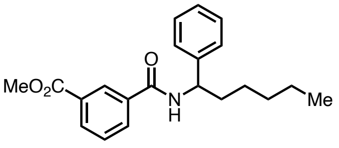

2j

methyl 3-((1-phenylhexyl)carbamoyl)benzoate (**2j**)

The general procedure for the α-arylation of benzamides was followed using **1g** (106 mg, 0.400 mmol, 2.00 equiv), PC1 (4.4 mg, 0.004 mmol, 0.02 equiv), $NiCl_2$•DME (2.2 mg, 0.010 mmol, 0.05 equiv), BiOx (1.4 mg, 0.010 mmol, 0.05 equiv), K_3PO_4 (85 mg, 0.400 mmol, 2.00 equiv), TBABr (64 mg, 0.200 mmol, 1.00 equiv), and bromobenzene (21 uL, 0.200 mmol, 1.00 equiv) in 1 mL EtOAc. Analysis by ^{1}H NMR using dibromomethane as an internal standard showed 58% yield of the desired product. Purification by silica gel chromatography (70:30 hexanes/EtOAc) gave the title compound as a white solid (37.6 mg, 0.092 mmol, 46% yield).

Rf: 0.35 (80:20 hexanes/EtOAc)

^{1}H NMR (400 MHz, Chloroform-*d*) δ 8.35 (s, 1H), 8.14 (d, *J* = 7.6 Hz, 1H), 8.03 (d, *J* = 7.8 Hz, 1H), 7.51 (t, *J* = 7.8 Hz, 1H), 7.40 – 7.30 (m, 5H), 7.28 (d, *J* = 3.1 Hz, 1H), 6.44 (d, *J* = 8.2 Hz, 2H), 5.17 (q, *J* = 7.6 Hz, 2H), 3.93 (s, 3H), 1.93 (dq, *J* = 16.4, 9.4, 7.1 Hz, 2H), 1.33 (m, 6H), 0.86 (t, *J* = 6.7 Hz, 3H).

^{13}C NMR (176 MHz, Chloroform-*d*) δ 166.55, 165.69, 142.26, 135.08, 132.52, 132.14, 130.54, 129.08, 128.91, 127.66, 127.48, 126.84, 54.30, 52.57, 36.26, 31.71, 26.16, 22.65, 14.17.

HRMS: (ESI) (m/z): [M+H] calculated for $C_{21}H_{25}NO_3$, 340.1912, found 340.1913.

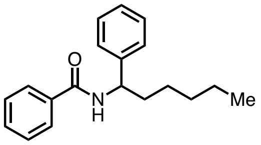

2k

N-(1-phenylhexyl)benzamide (**2k**)

The general procedure for the α-arylation of benzamides was followed using **1h** (86 mg, 0.400 mmol, 2.00 equiv), PC1 (4.4 mg, 0.002 mmol, 0.01 equiv), $NiBr_2$•DME (6.2 mg, 0.020 mmol, 0.10

equiv), 5,5’-diMeBpy (5.5 mg, 0.030 mmol, 0.15 equiv), K_3PO_4 (85 mg, 0.400 mmol, 2.00 equiv), TBABr (64 mg, 0.200 mmol, 1.00 equiv), and bromobenzene (21 uL, 0.200 mmol, 1.00 equiv) in 1 mL 1,4-dioxane. Purification by silica gel chromatography (100% hexanes to 85:15 hexanes/EtOAc) gave the title compound as a white solid (37.1 mg, 0.132 mmol, 66% yield). The spectral data matches that previously reported in the literature.[11]

Rf: 0.60 (70:30 hexanes/EtOAc)

^{1}H NMR (500 MHz, Chloroform-*d*) δ 7.76 (d, *J* = 7.6 Hz, 2H), 7.48 (t, *J* = 7.5 Hz, 1H), 7.41 (t, *J* = 7.5 Hz, 2H), 7.38 – 7.31 (m, 5H), 7.27 (d, *J* = 8.0 Hz, 1H), 6.40 (d, *J* = 9.2 Hz, 1H), 5.16 (q, *J* = 7.7 Hz, 1H), 1.90 (ddt, *J* = 23.5, 16.9, 8.3 Hz, 2H), 1.33 (q, *J* = 19.6, 11.8 Hz, 6H), 0.86 (t, *J* = 6.3 Hz, 3H).

^{13}C NMR (126 MHz, Chloroform-*d*) δ 166.79, 142.56, 134.82, 131.53, 128.81, 128.65, 127.48, 127.03, 126.75, 54.06, 36.38, 31.71, 26.12, 22.63, 14.15.

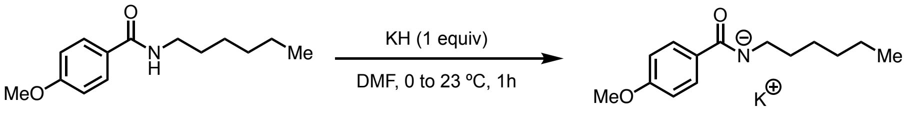

1d S7

To a suspension of KH (50% w/w in paraffin) (80.2 mg, 1.0 mmol, 1.0 equiv) in 5 mL DMF at 0 ºC was added dropwise a solution of **1d** (235 mg, 1.0 mmol, 1.00 equiv) in 5 mL DMF. The reaction was stirred at 0 ºC for 30 min, then warmed to 23 ºC for 30 min. The solution was used without purification.[10]

IV) Controls, Additive Screening, and Mechanistic Insights:

We did not observe any regioisomers or olefin byproducts in our reaction that would suggest capture of a distal radical or β-hydride elimination/ reinsertion of a metal hydride.

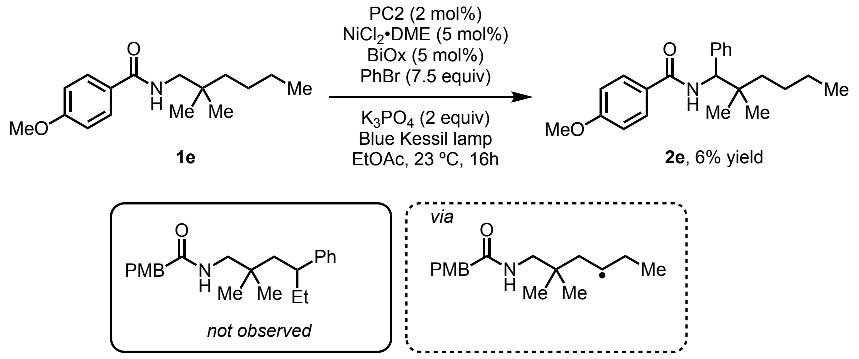

Discussion: α-arylation is still observed in substrates where a 1,5-HAT and/or chain walking is not possible such as *N*-benzylbenzamide or substrates containing a gem-dimethyl group in the alkyl chain. We believe the yield for the latter case is low due to sterics.

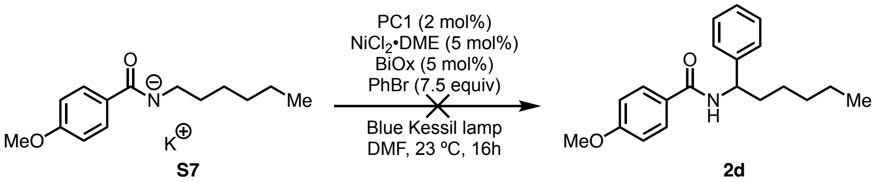

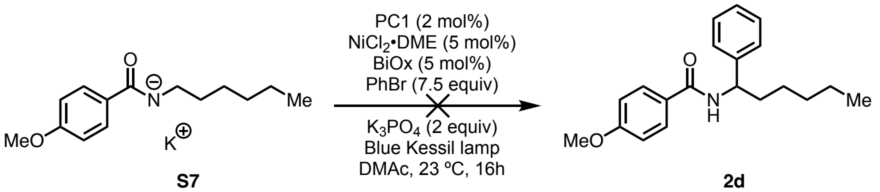

Discussion: The Rovis group found that potassium salts of triflamides are viable substrate for α-alkylation, and CV studies suggest direct oxidation of their substrate by the photocatalyst was a viable pathway. However, when testing the potassium salt of our benzamide, no product is observed suggesting that deprotonation of benzamide is not a viable pathway for α-amidyl radical formation.[12]

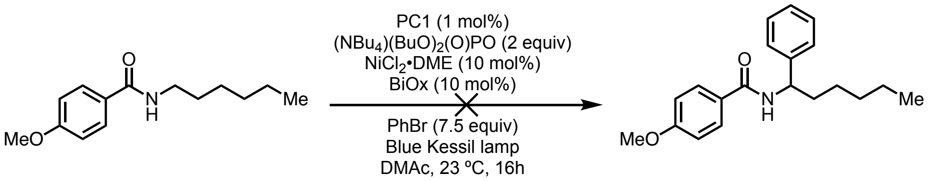

Discussion: Use of a more soluble phosphate base did not lead to any product formation. This could suggest that the role of the base is to sequester acid generated during reaction. The lack of reactivity might be due to competitive association of phosphate with the photocatalyst, as observed by Knowles and Alexanian with related iridium photocatalysts.[11]

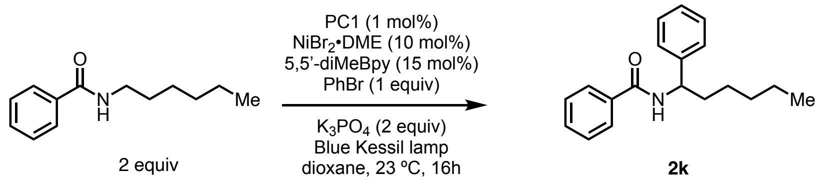

1h

With 1 equiv TBABr: 66% yield
Previously reported: 60% yield

Discussion: Using conditions previously developed for the arylation of benzamides using a $NiBr_2$•DME/ 5,5'-diMeByp catalytic system, modest increases in yield were observed.

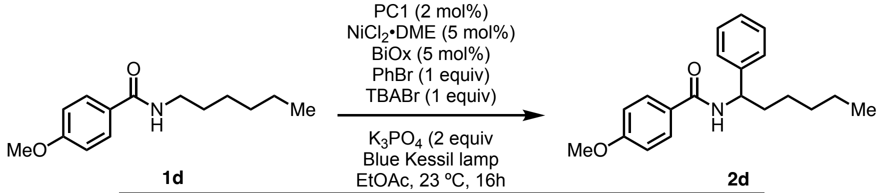

entry	Deviation from above	% yield [a]
1	none	70
2	10% TBAB	62
3	$NiBr_2$•dme, no TBAB	50
4	$NiBr_2$•dme, 1 eq TBAB	75

[a] Reaction was carried out with 0.20 mmol PhBr, 0.40 mmol benzamide. Yield determined by ^1H NMR using dibromoethane as an internal standard.

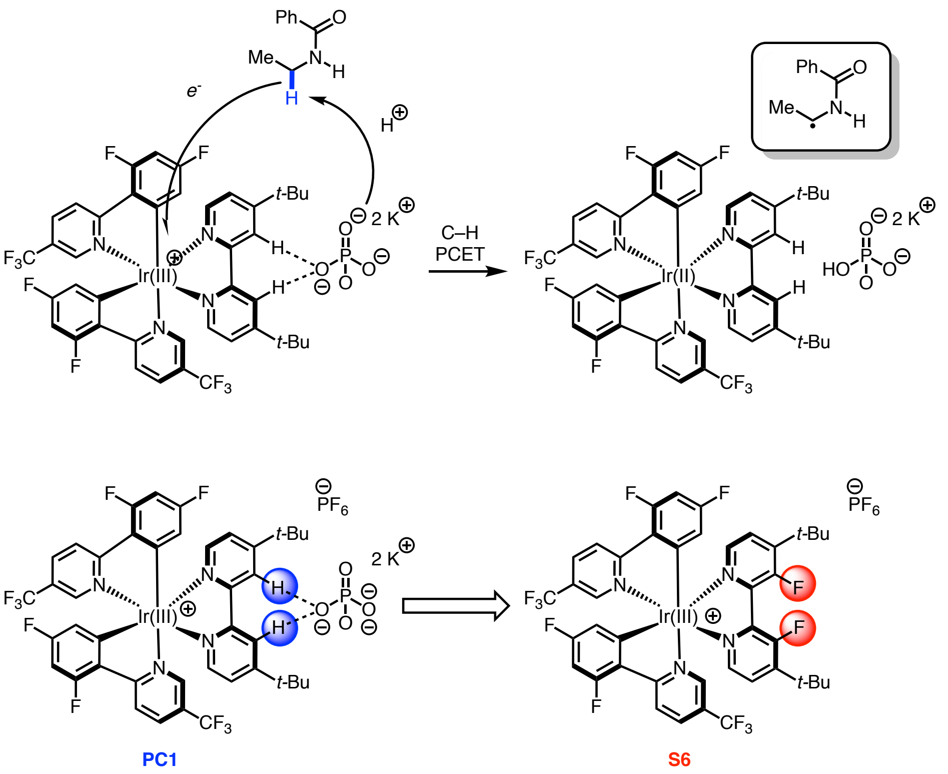

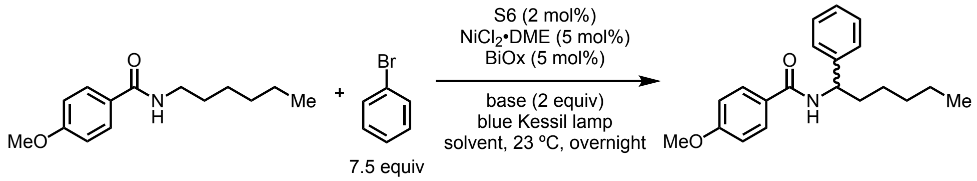

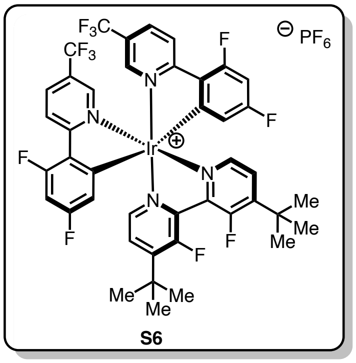

Conditions:
1) EtOAc, K_3PO_4 6% (91% RSM)
2) DMF, Cs_2CO_3 14% (86% RSM)

Discussion: The Knowles and Alexanian groups found that hydrogen-bonding of an HAT agent to the photocatalyst was necessary for C–H PCET to occur in specific systems. Blocking the 3,3'-positions of a bipyridyl ligand on their photocatalyst resulted in no formation of product. When testing this hypothesis in our system, we observed some product formation when using a photocatalyst (**S6**) that was not able to engage in hydrogen bonding at the 3,3'-positions of a bipyridyl ligand. Based on these results, we hypothesis that hydrogen bonding is not necessary for productive catalysis to occur, though it may serve to more effectively generate an HAT agent through favorable complexation and electron transfer. Furthermore, the exceedingly low solubility of K_3PO_4 in EtOAc makes phosphate an unlike HAT agent in this reaction.[13]

CV data:

Cyclic voltammetry was performed in a nitrogen-filled glovebox with a Biologic VSP multichannel potentiostat/galvanostat using a three electrode electrochemical cell, consisting of a glassy carbon disk working electrode (0.07 cm^2, BASi), a Ag/Ag+ quasi-reference electrode (BASi) with 0.01 M $AgBF_4$ (Sigma) in acetonitrile, and a platinum wire counter electrode (ALS). The glassy carbon disk electrode was polished in a nitrogen-filled glovebox using aluminum oxide polishing paper (9 micron and 0.3 micron, Fiber Instrument) and anhydrous acetonitrile. All experiments were run in the 0.5 M $TBAPF_6$ stock electrolyte with a scan rate of 100mV/s.

Figure S1: CV of **1d**

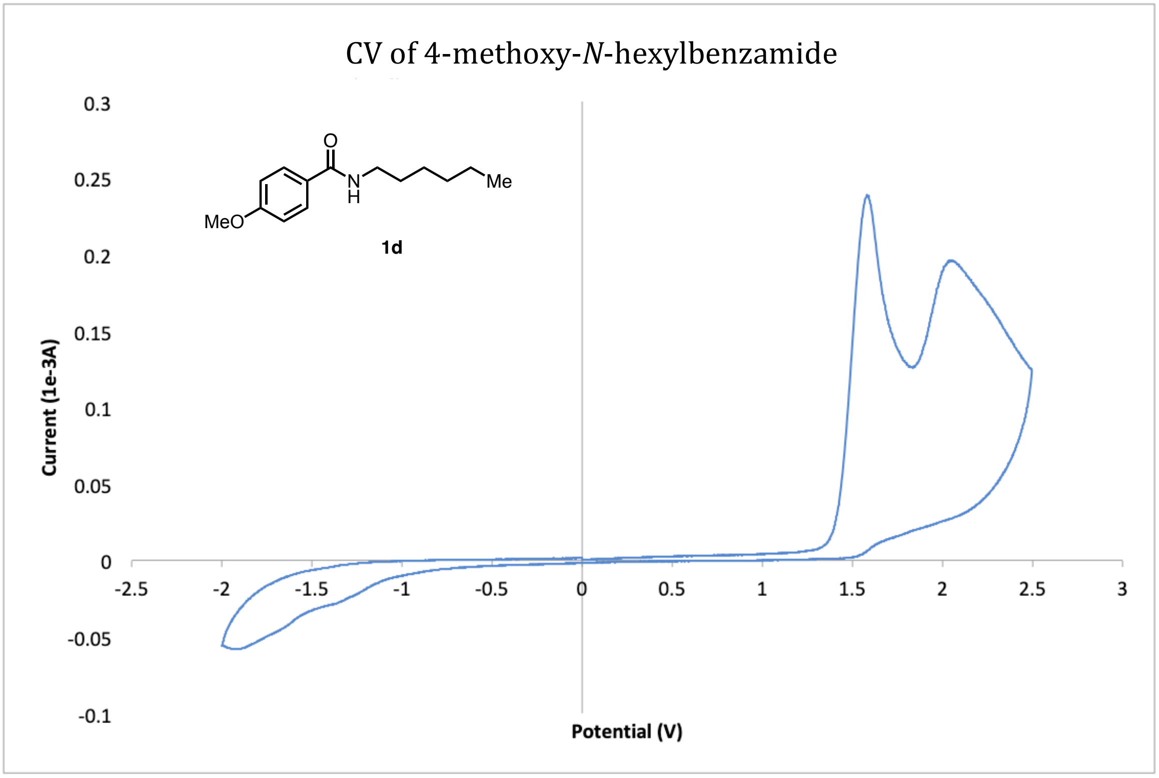

Figure S2: CV of **2d**

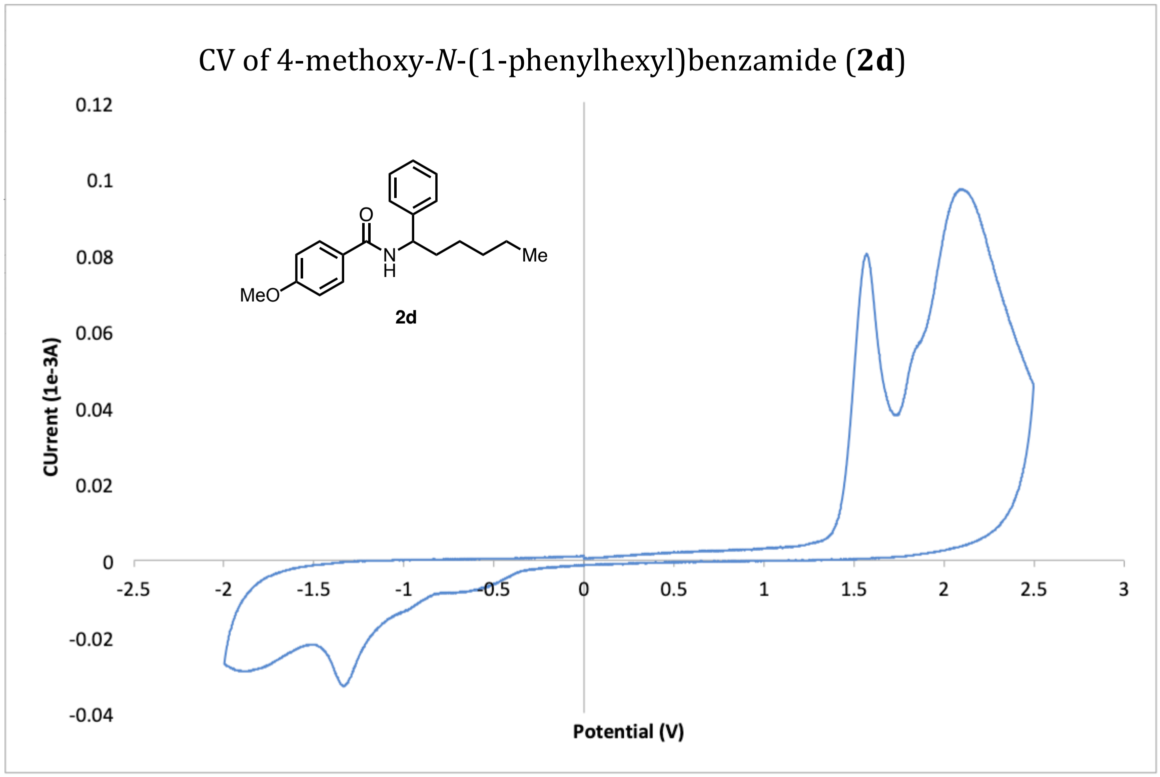

Figure S3: CV of *i*-PrBiOxNiCl$_2$

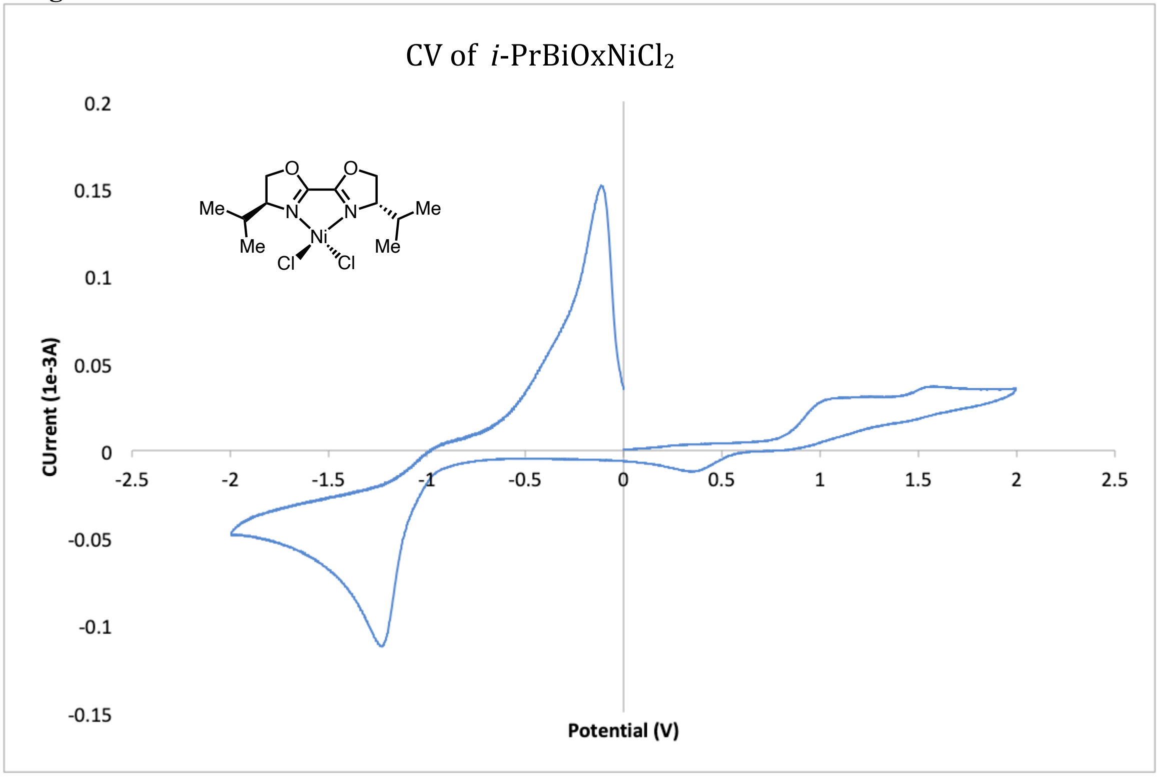

Figure S4: CV of **1d** and *i*-PrBiOxNiCl$_2$

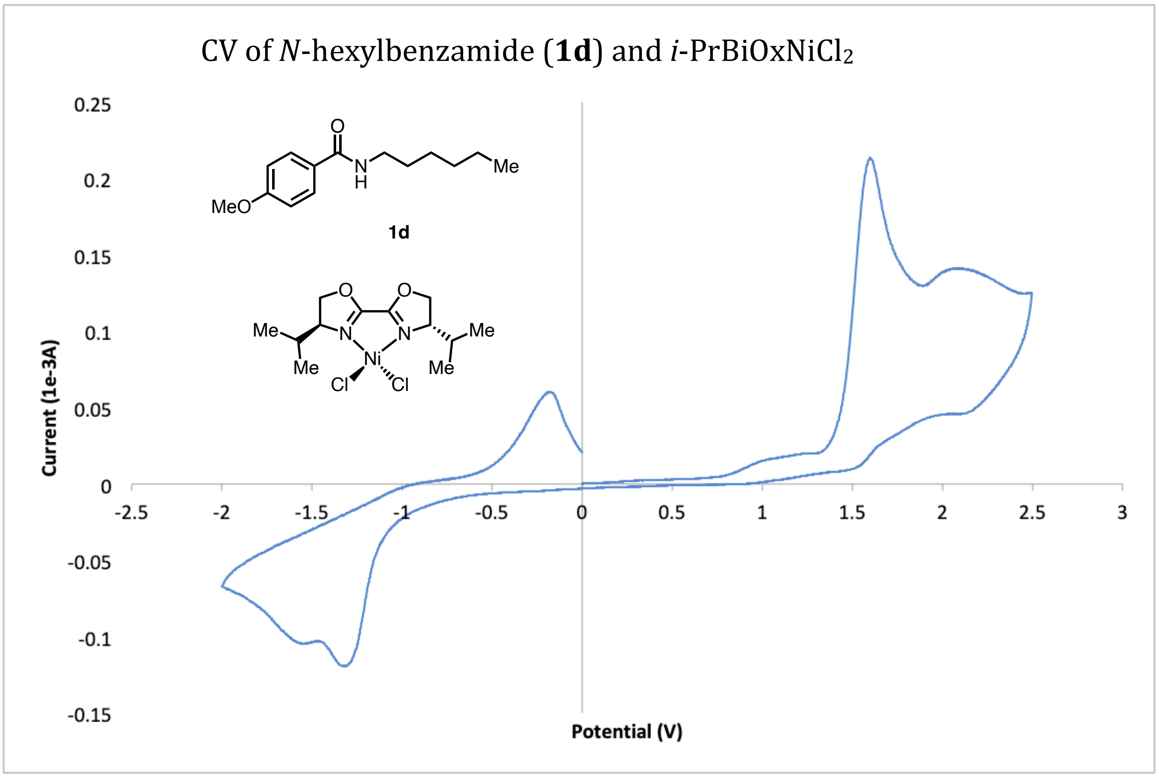

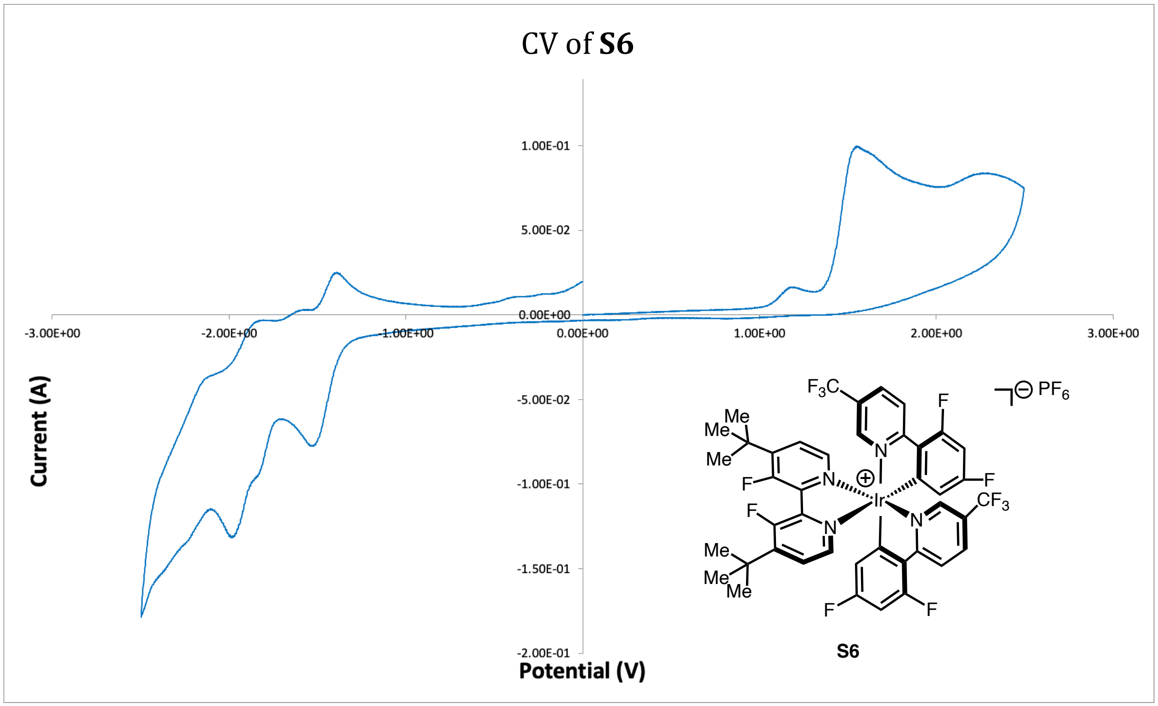

Ir(III/II)= -1.42V vs Ag/Ag+ (-1.12V vs SCE)
Ir(III/IV)= 1.15V vs Ag/Ag+ (1.45V vs SCE)
Ir(III*/II)= 1.16V vs Ag/Ag+ (1.47V vs SCE)
Ir(III*/IV)= -1.43 vs Ag/Ag+ (-1.13V vs SCE)

VI) Stern-Volmer Quenching Experiment

Stern-Volmer studies were carried out with a Horiba PTI QuantaMaster 8000. The standard solutions were prepared in EtOAc in a nitrogen-filled glovebox and sealed with a Teflon-lined cap prior to removing. The solutions (0.01 mM PC1 photocatalyst and varying concentrations of benzamide, PhBr, *i*PrBiOxNiCl$_2$, and TBABr) were irradiated at 380 nm and luminescence was measured at 470 nm. I_0/I values were determined from the average of three runs per quencher concentration. Potassium phosphate was not sufficiently soluble in EtOAc in order to run this analysis.

Figure S5: Quenching experiments with constant **PC1** and varied **1d**

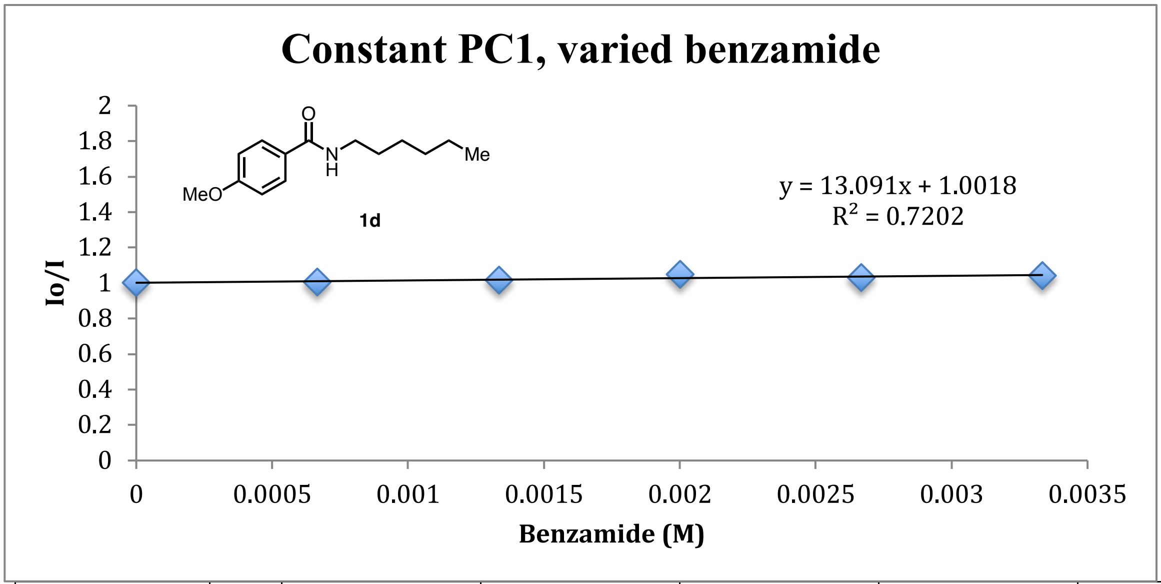

amide conc.	0	0.6667mM	1.3333mM	2.0000mM	2.6667mM	3.3333mM
Io/I	1	1.006289051	1.016982421	1.048302737	1.028892087	1.041263284

Figure S6: Quenching experiments with constant **PC1** and varied bromobenzene

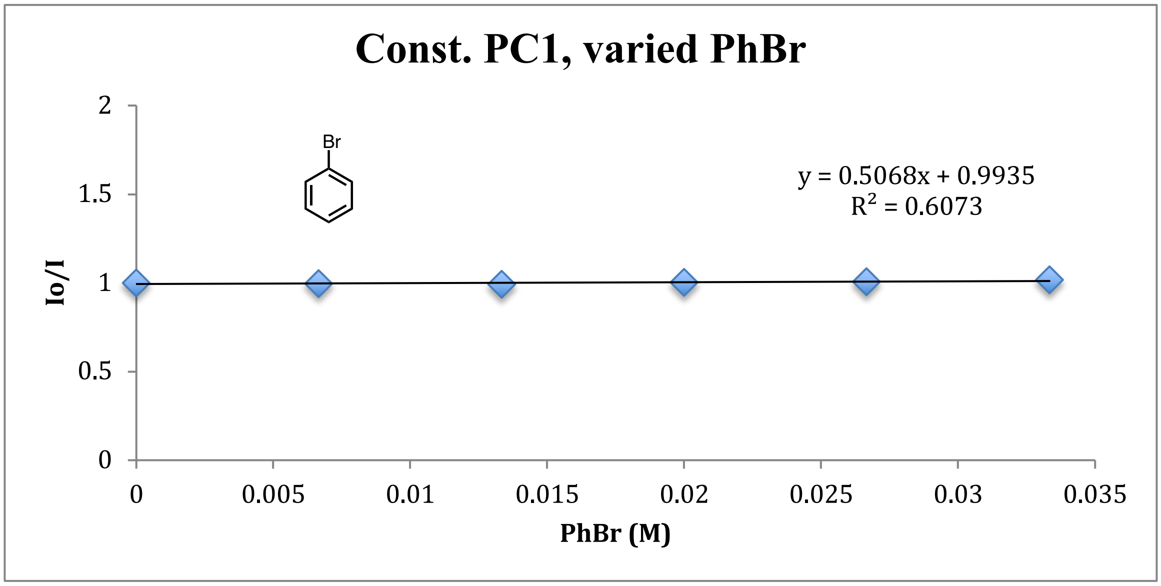

PhBr conc.	0	6.6667mM	13.3333mM	20.0000mM	26.6667mM	33.3333mM
Io/I	1	0.995529451	0.993125325	1.001485081	1.005943129	1.015730284

Figure S7: Quenching experiments with constant iridium and varied *i*PrBiOxNiCl$_2$

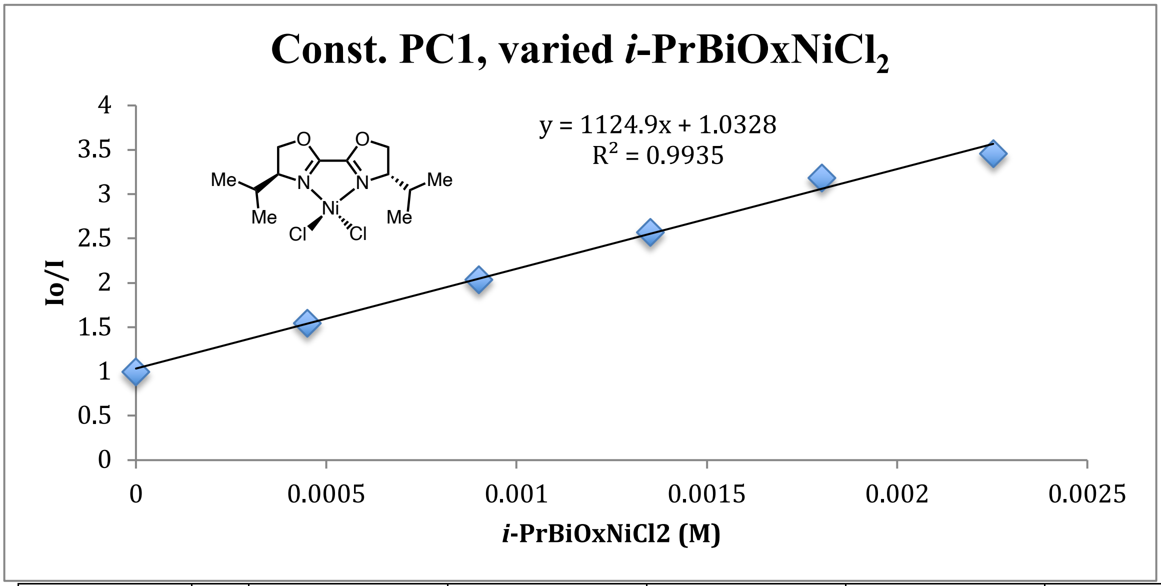

Ni(II) conc.	0	0.4505mM	0.9010mM	1.3516mM	1.8021mM	2.2527mM
Io/I Run 1	1	1.548728128	1.9748611	2.556606257	3.090001208	3.575230128
Io/I Run 2	1	1.549864056	2.114234165	2.580339342	3.126187733	3.375772646
Io/I Run 3	1	1.547807227	2.020428729	2.568158825	3.344921814	3.424462848
Average	1	1.548799803	2.036507998	2.568368141	3.187036918	3.458488541

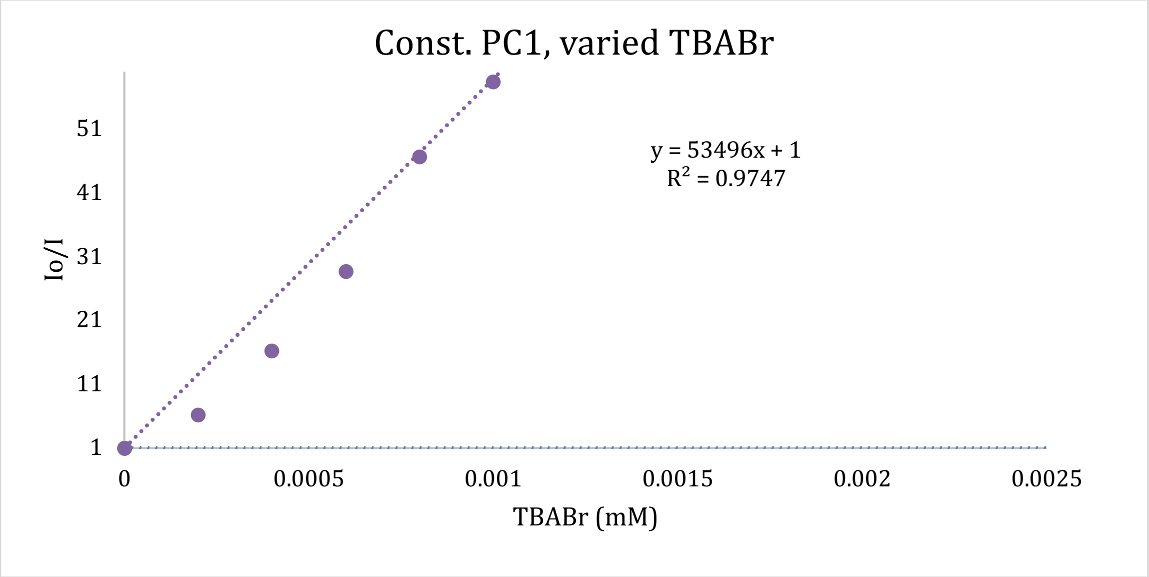

TBABr conc.	0	0.0002mM	0.0004mM	0.0006mM	0.0008mM	0.001mM
Io/I Run 1	1	6.24154696	16.2213235	28.7356699	46.2204003	57.9773198
Io/I Run 2	1	6.20156044	16.2949751	28.6717123	47.0402301	58.8707863
Average	1	6.2215537	16.2581493	28.7036911	46.6303152	58.4240531

Discussion: Stern-Volmer experiments show that **1d** and bromobenzene do not quench the photocatalyst. *i*-PrBiOxNiCl_2 and TBABr shows quenching by our photocatalyst. This is unsurprising since *i*-PrBiOxNiCl_2 and TBABr are within the reduction potential of **PC1**. From this data we are unable to discern to what extent *i*-PrBiOxNiCl_2 and TBABr respectively quench **PC1** in the reaction, though the high quenching constant of TBABr suggests that this is a more likely pathway. This data also suggests that *i*-PrBiOxNiCl_2 quenches **PC1** in the absence of TBABr, which explains why productive catalysis is observed in the absence of TBABr. The poor solubility of K_3PO_4 in EtOAc prohibited us from measuring any Stern-Volmer quenching.

VII) NMR Titration Studies

The concentration of iridium-bromide complex in solution was calculated through measuring the change in ^{1}H NMR in acetone-d_6 of the 3,3'-position of the bipyridyl ligand on iridium (δ) and the chemical shift without any bromide (δ_0). $[Ir^+]_0$ is the concentration of iridium added to solution, and $[Br^-]_0$ is the concentration of TBABr added to the solution. The equilibrium constants at several concentrations were then calculated and averaged to give the K_{eq} for iridium-bromide complexation.[13]

$$\delta = \frac{[Ir^+, Br^-] \times \delta_{final} + ([Ir^+]_0 - [Ir^+, Br^-]) \times \delta_0}{[Ir^+]_0}$$

$$K_{eq} = \frac{[Ir^+, Br^-]}{([Ir^+]_0 - [Ir^+, Br^-]) \times ([Br^-]_0 - [Ir^+, Br^-])}$$

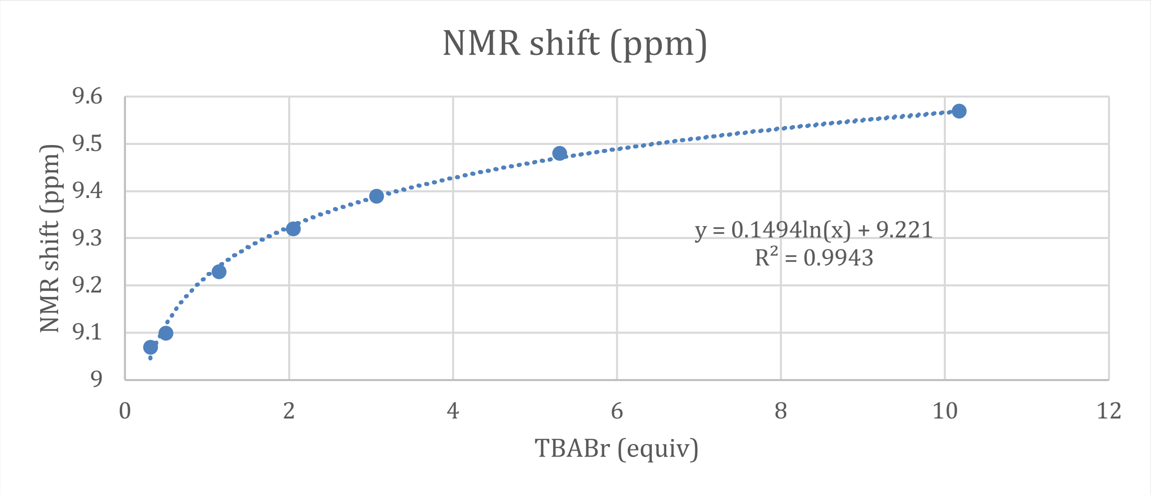

equiv TBABr	NMR shift (ppm)	change in ppm		$[Ir^+,Br^-]$	K_{eq}
0.00	8.93	0.00			
0.31	9.07	0.14		0.00043675	1524.575056
0.50	9.10	0.17		0.00053034	782.3765411
1.15	9.23	0.30		0.00093589	648.9742213
2.05	9.32	0.39		0.00121665	542.0392478
3.07	9.39	0.46		0.00143503	542.928503
5.30	9.48	0.55		0.00171579	687.1718
10.17	9.57	0.64		0.00199656	
				Average	788.0108949

VIII) UV-Vis Titration Studies

UV-Vis studies were carried out with a Varian, Cary 100 Bio UV-Vis Spectrometer.

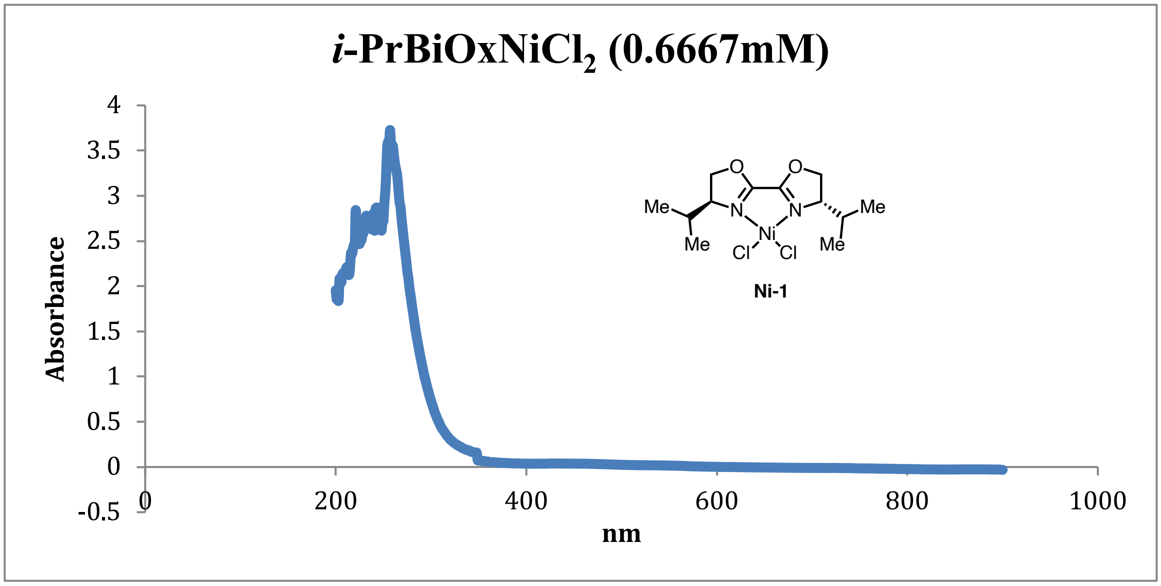

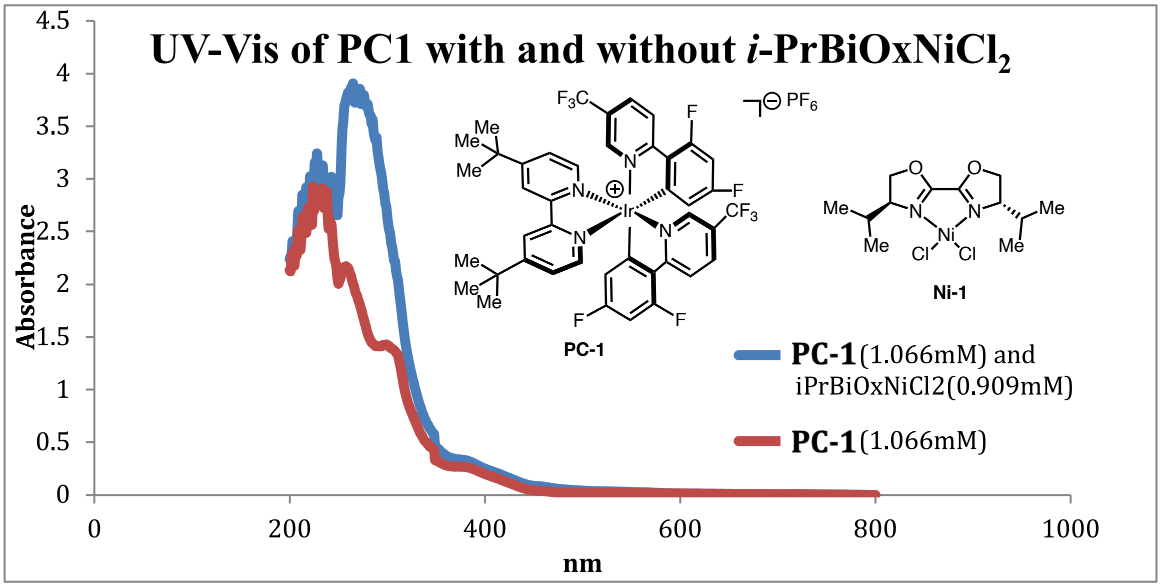

Discussion:

UV-Vis shows that **29** does not strongly absorb light in the blue region. Similarly, there is no effect on PC1 when near equal molar quantities of **29** are present.

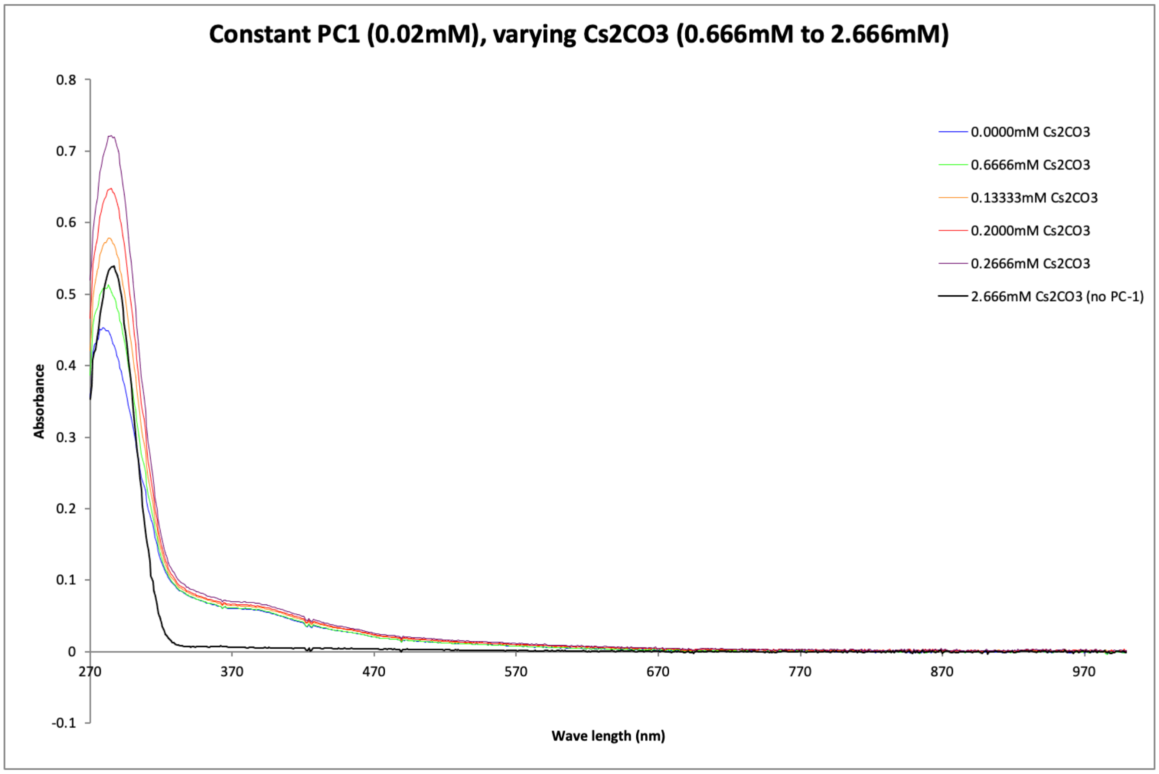

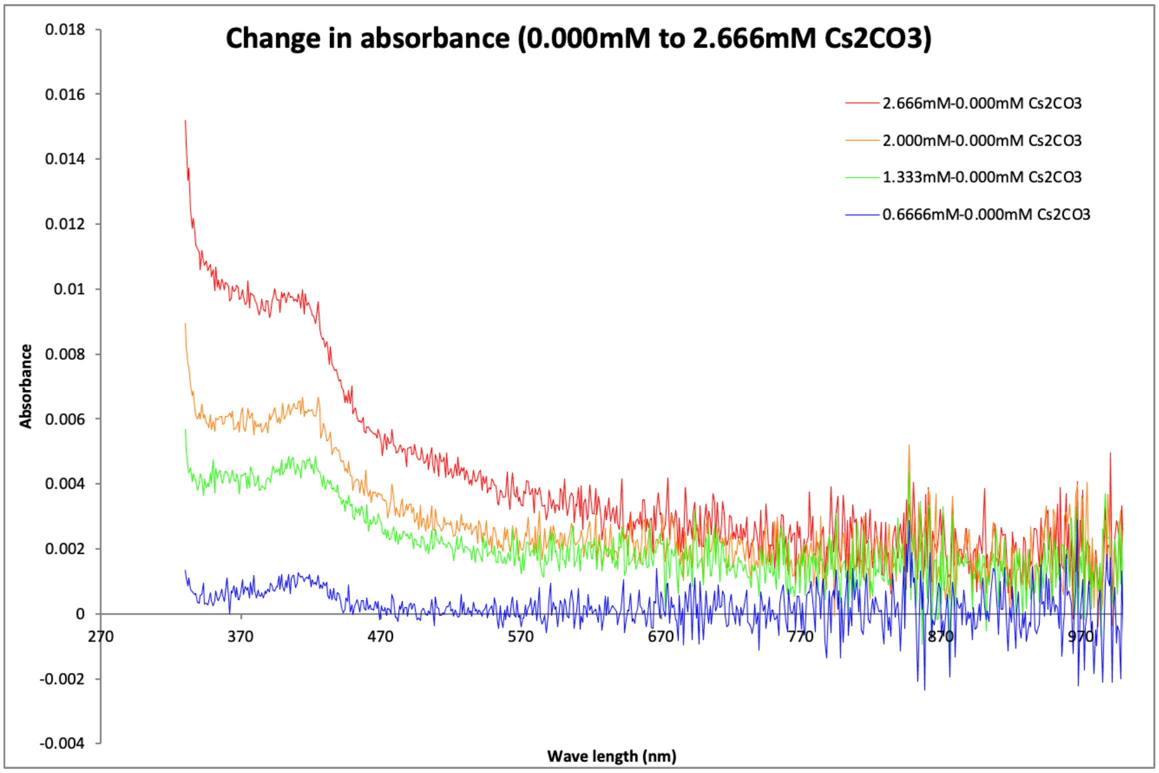

Discussion: To test the possibility of a multi-site C–H PCET in our system, we first undertook UV-Vis titration experiments to see if it was feasible for a base to coordinate to the bipyridyl of PC1. Due to the poor solubility of K_3PO_4 in our reaction media, and the lack of reactivity observed with

more soluble potassium salts such as $(NBu_4)_2OP(O)(OPh)$ and $NBu_4OP(O)(OBu)_2$, we opted to probe the complexation between Cs_2CO_3 and PC1. During our optimization we found that, when substituting K_3PO_4 for Cs_2CO_3, the desire product was formed, albeit in slightly lower yields), which led us to believe this would be a fair comparison. When performing UV-Vis titration experiments with Cs_2CO_3 and PC1, little change was observed in the MLCT region (350-450 nm). This is contrary to the studies done by Knowles and Alexanian, which show large changes in absorption in this region when titrating $NBu_4OP(O)(OBu)_2$ and PC2.[13]

IX) NMR Spectra of Starting Material:

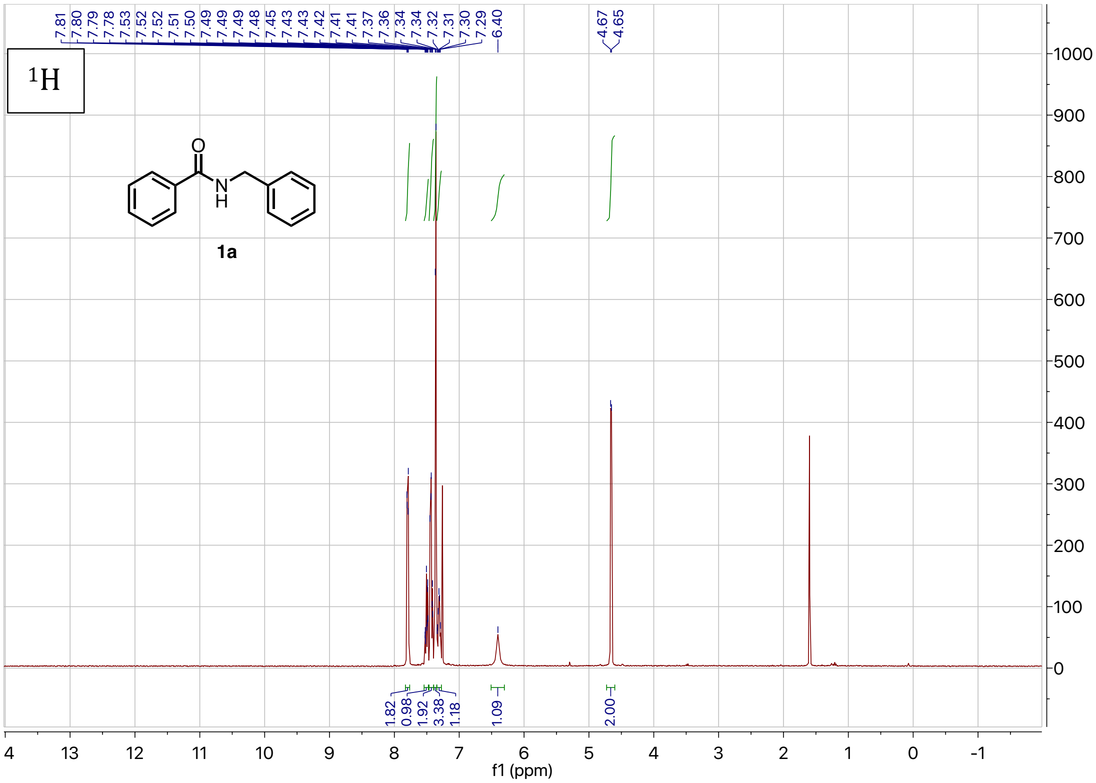

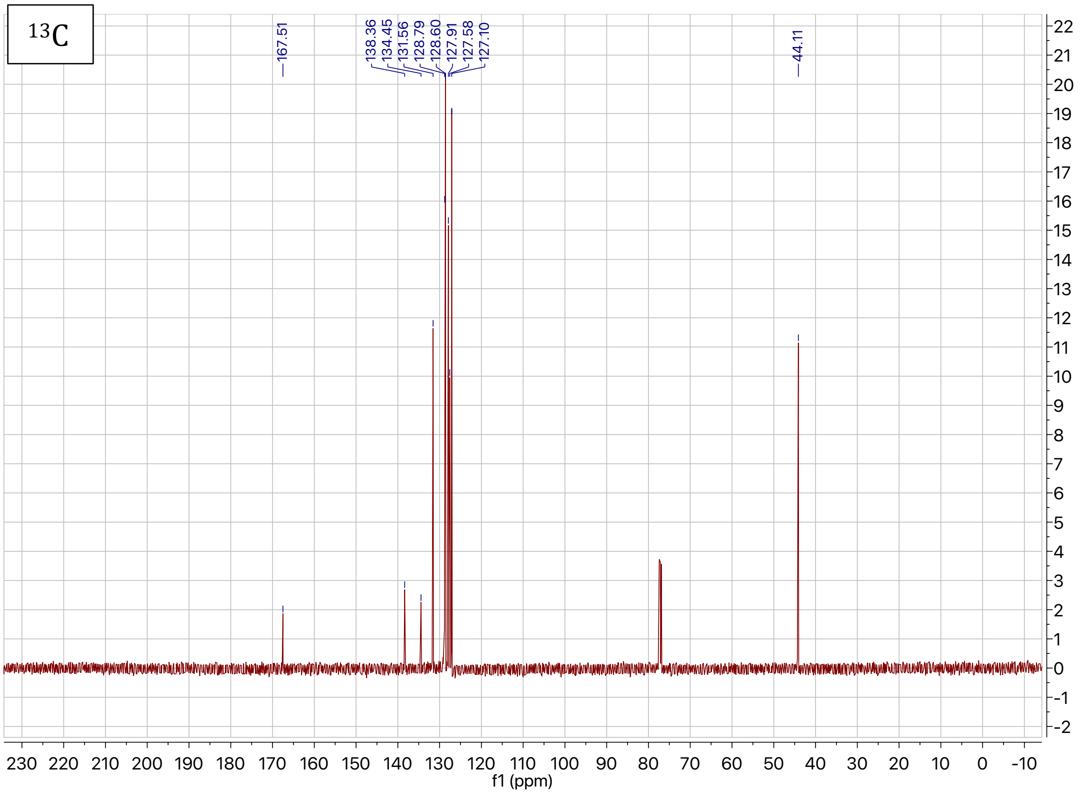

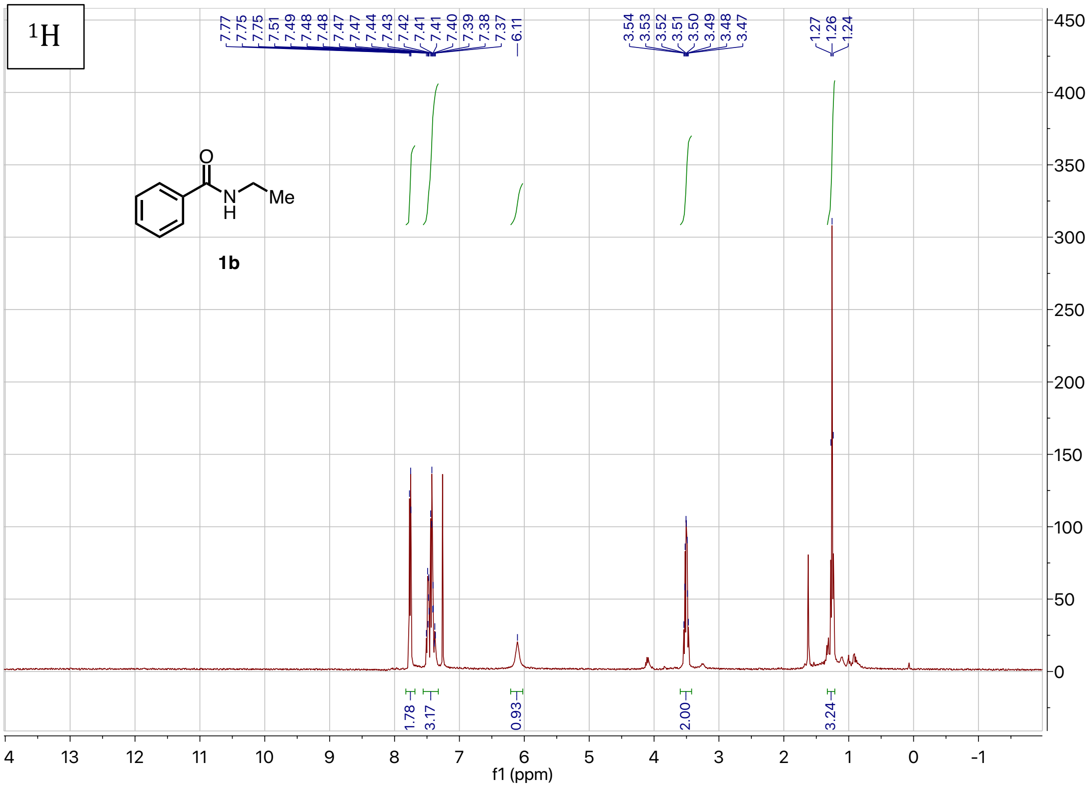

1H
7.77
7.75
7.75
7.51
7.49
7.48
7.48
7.47
7.47
7.44
7.43
7.42
7.41
7.41
7.40
7.39
7.38
7.37
6.11
3.54
3.53
3.52
3.51
3.50
3.49
3.49
3.48
3.47
1.27
1.26
1.24
Me
1b
1.78
3.17
0.93
2.00
3.24
f1 (ppm)

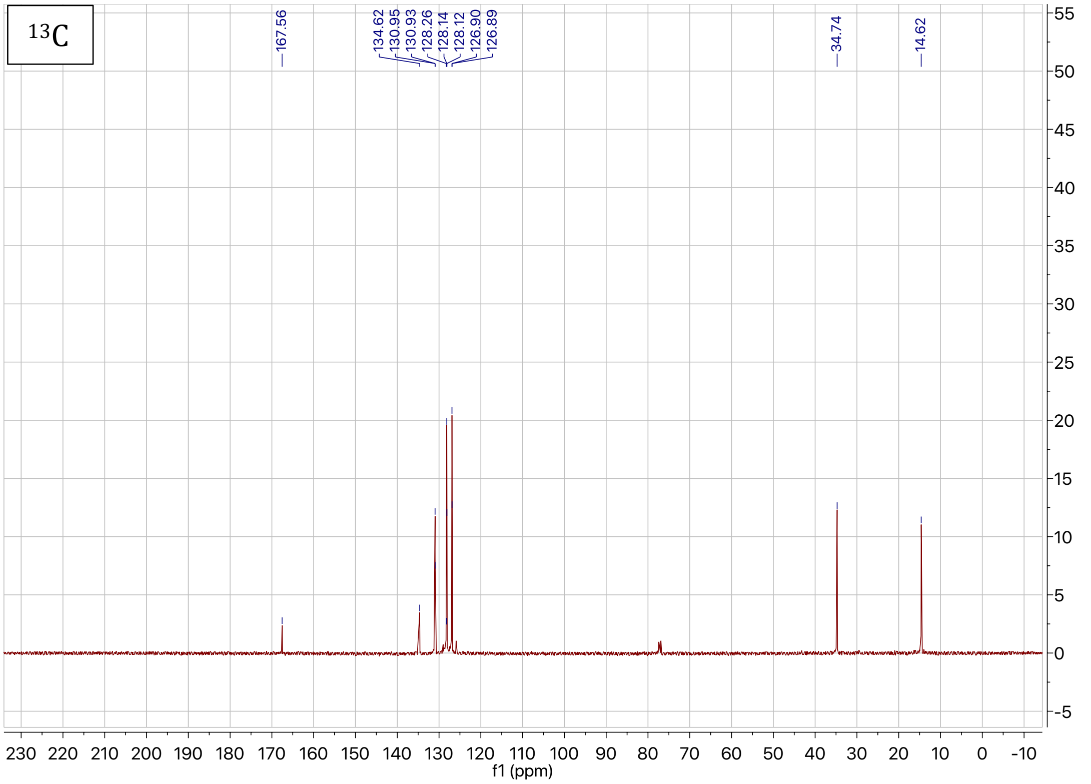

13C
167.56
134.62
130.95
130.93
128.26
128.14
128.12
126.90
126.89
34.74
14.62
f1 (ppm)

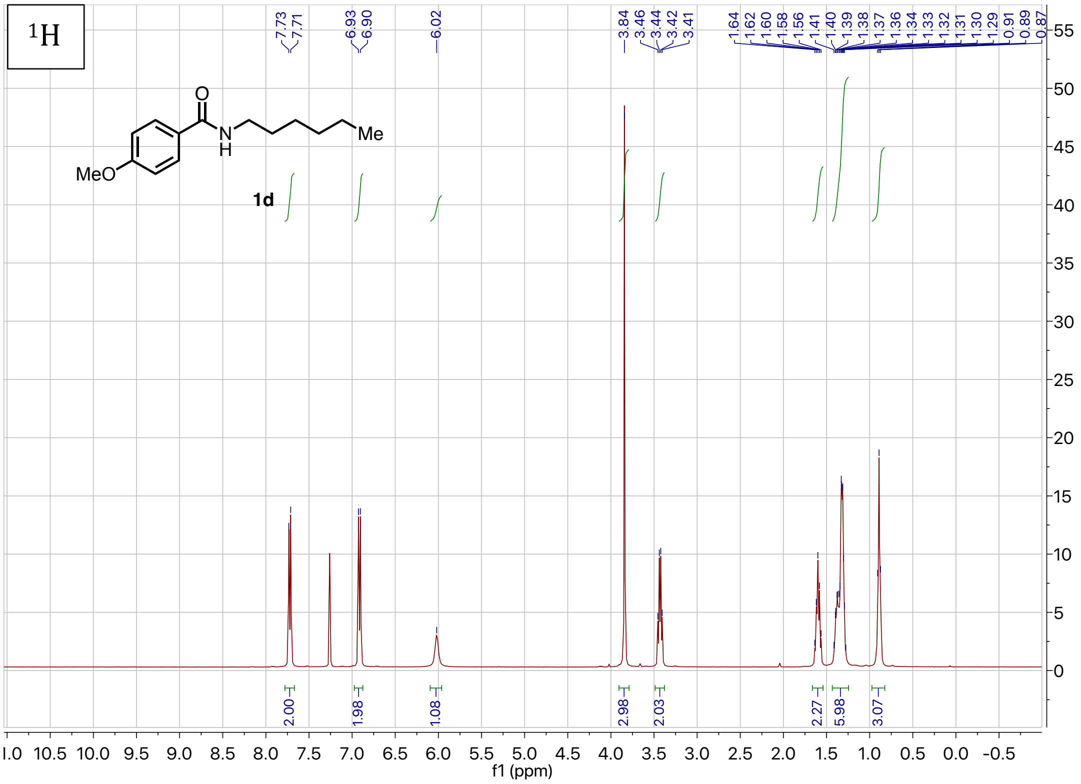

1H
7.73
7.71
6.93
6.90
6.02
3.84
3.46
3.44
3.42
3.41
1.64
1.62
1.60
1.58
1.56
1.41
1.40
1.39
1.38
1.37
1.36
1.34
1.33
1.32
1.31
1.30
1.29
0.91
0.89
0.87
O
N
H
Me
MeO
2.00
1.98
1.08
2.98
2.03
2.27
5.98
3.07
f1 (ppm)

1d

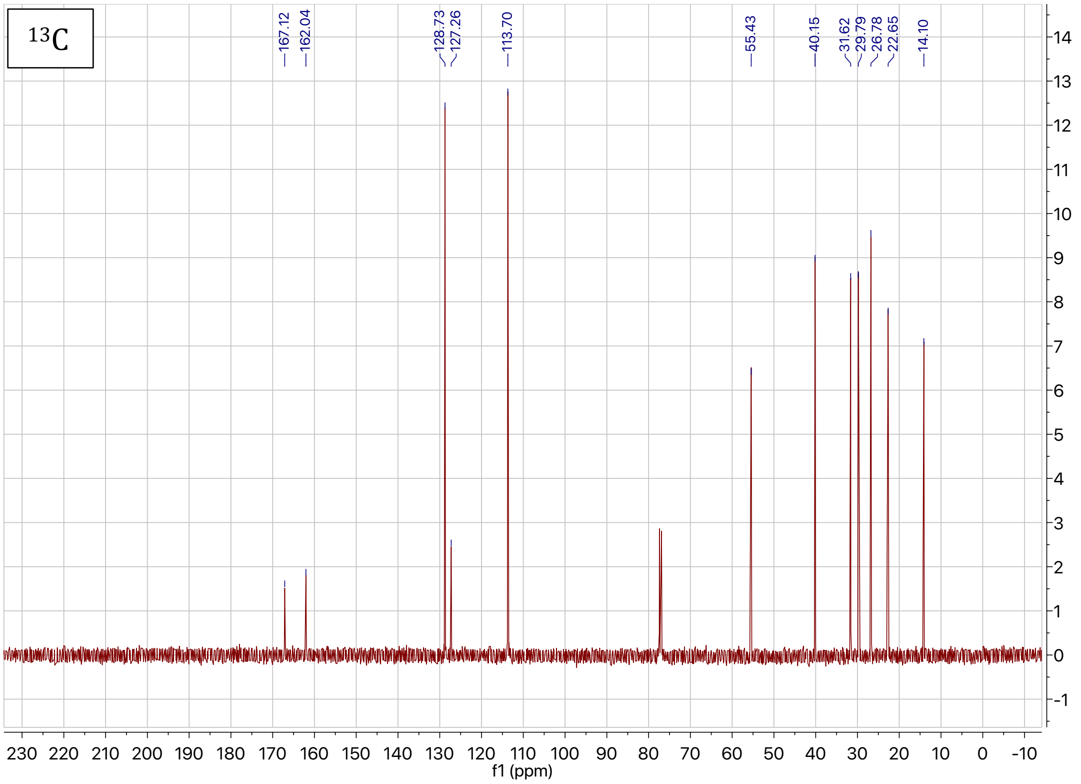

13C
167.12
162.04
128.73
127.26
113.70
55.43
40.15
31.62
29.79
26.78
22.65
14.10
f1 (ppm)

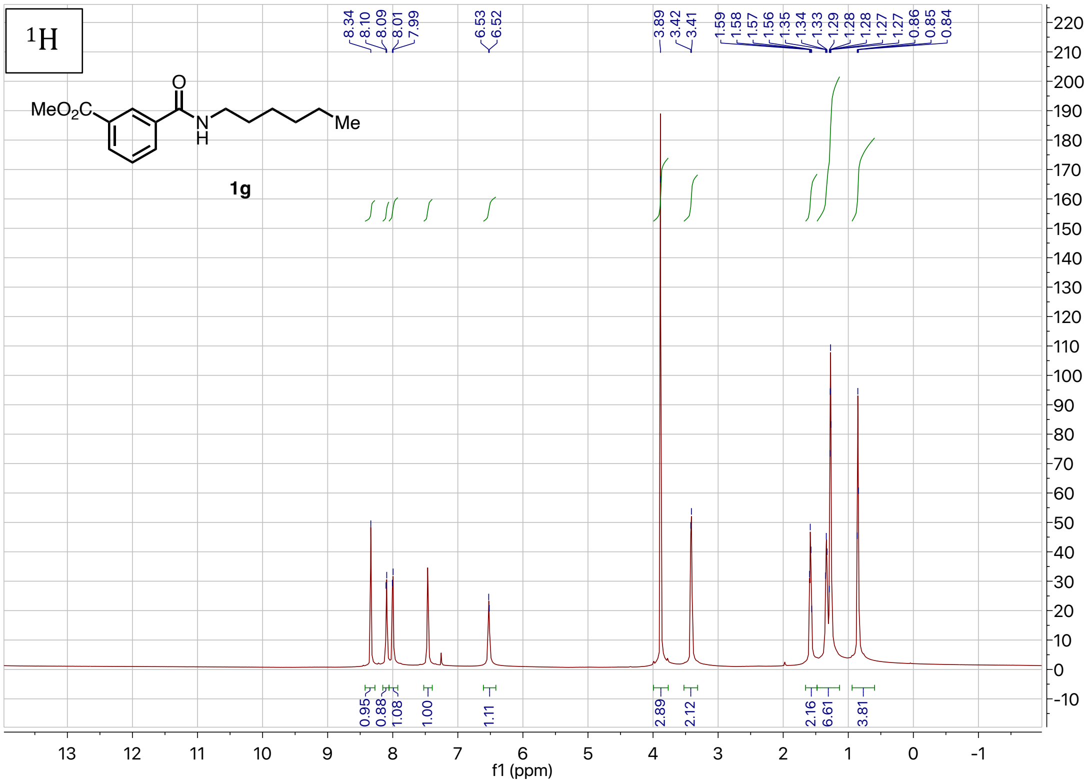
1H
MeO2C
O
N
H
Me
1g
8.34
8.10
8.09
8.01
7.99
6.53
6.52
3.89
3.42
3.41
1.59
1.58
1.57
1.56
1.35
1.34
1.33
1.29
1.28
1.28
1.27
1.27
0.86
0.85
0.84
0.95
0.88
1.08
1.00
1.11
2.89
2.12
2.16
6.61
3.81
f1 (ppm)

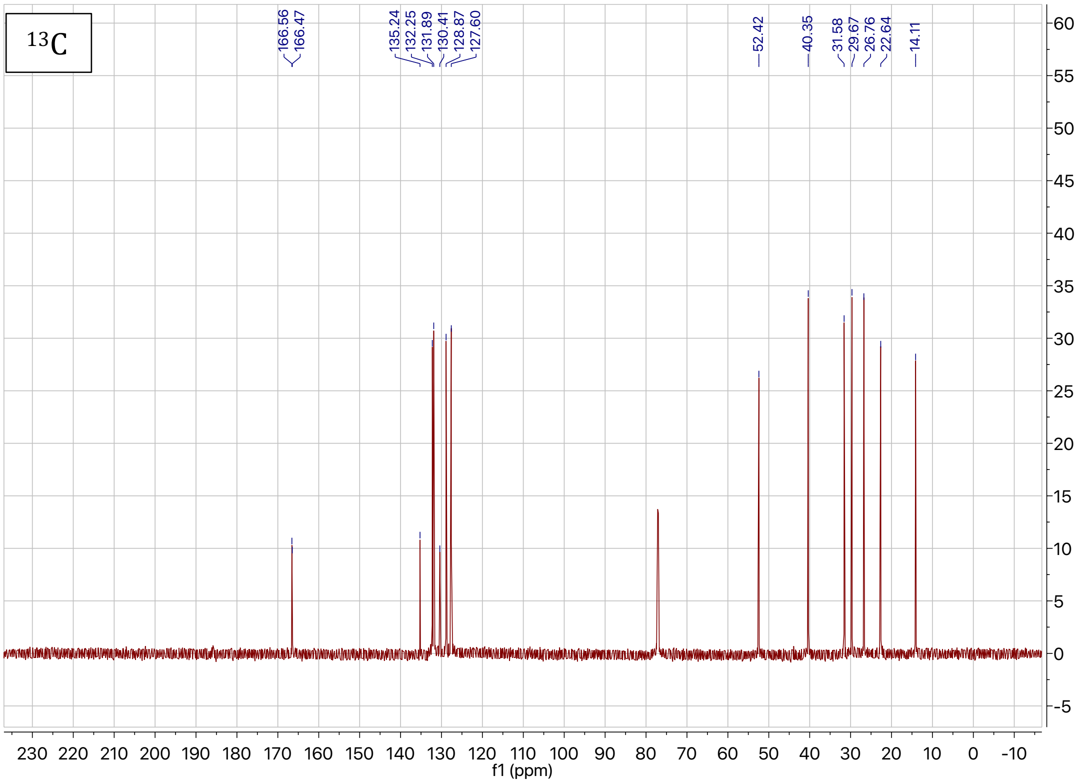
13C
166.56
166.47
135.24
132.25
131.89
130.41
128.87
127.60
52.42
40.35
31.58
29.67
26.76
22.64
14.11
f1 (ppm)

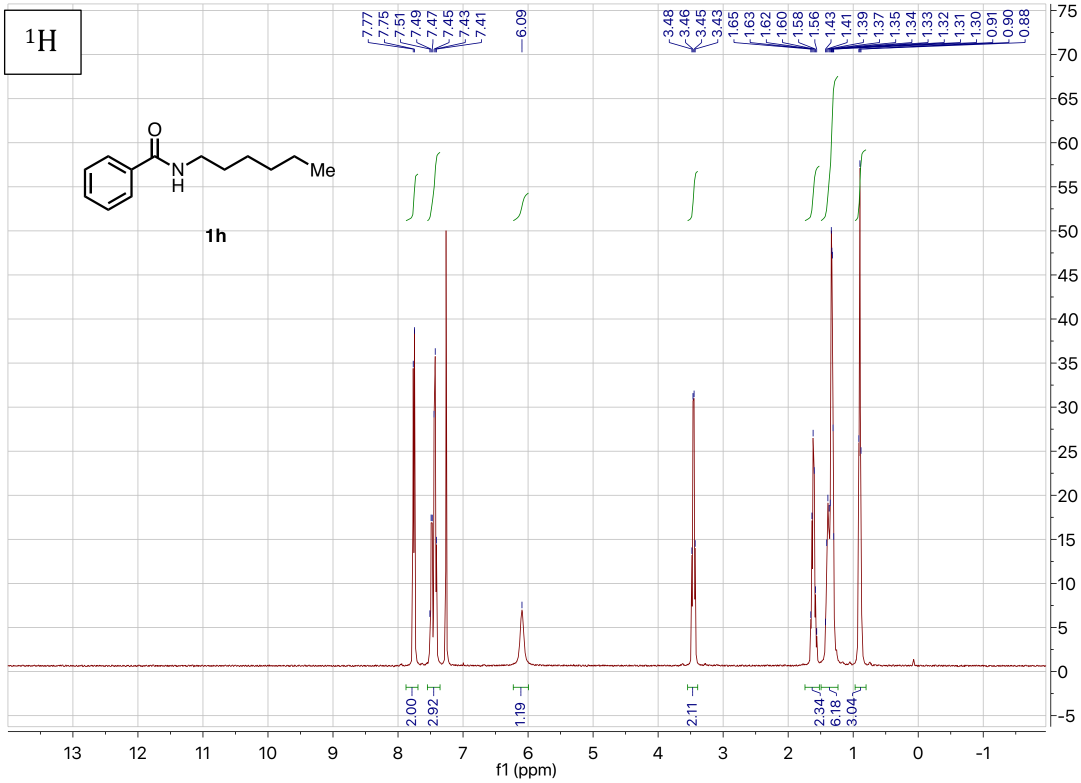
1H
1h
Me
7.77 7.75 7.51 7.49 7.47 7.45 7.43 7.41
6.09
3.48 3.46 3.45 3.43 1.65 1.63 1.62 1.60 1.58 1.56 1.43 1.41 1.39 1.37 1.35 1.34 1.33 1.32 1.31 1.30 0.91 0.90 0.88
2.00 2.92 1.19 2.11 2.34 6.18 3.04
f1 (ppm)

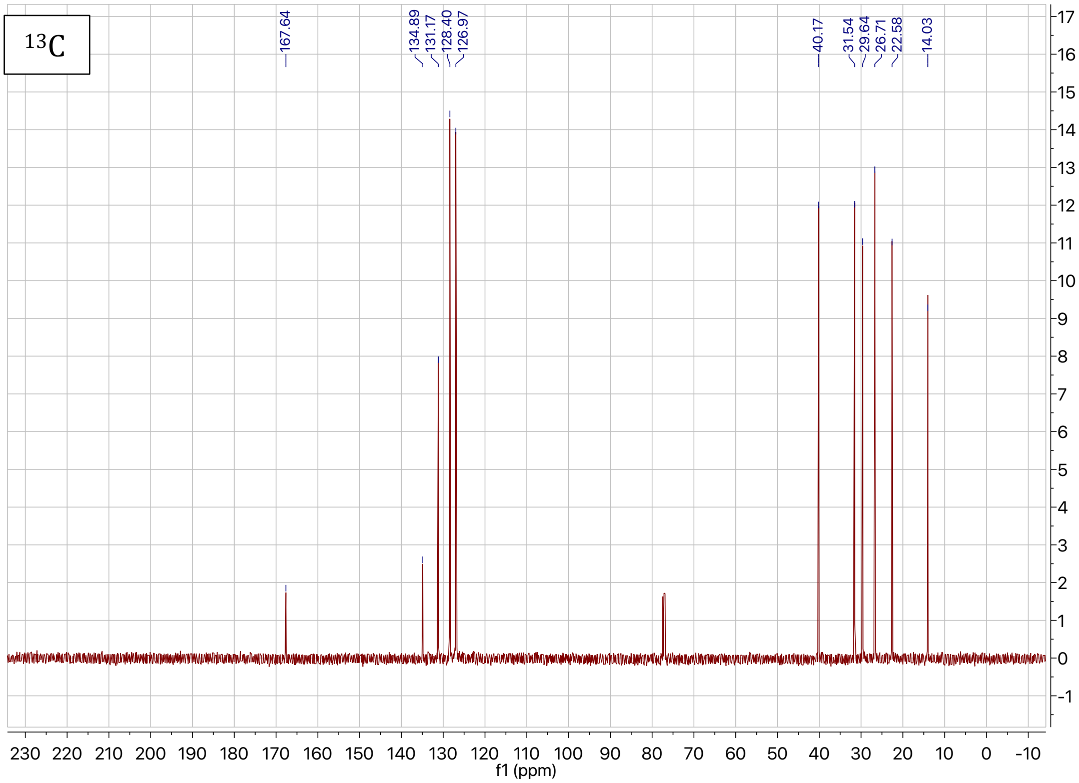
13C
167.64
134.89 131.17 128.40 126.97
40.17
31.54 29.64 26.71 22.58
14.03
f1 (ppm)

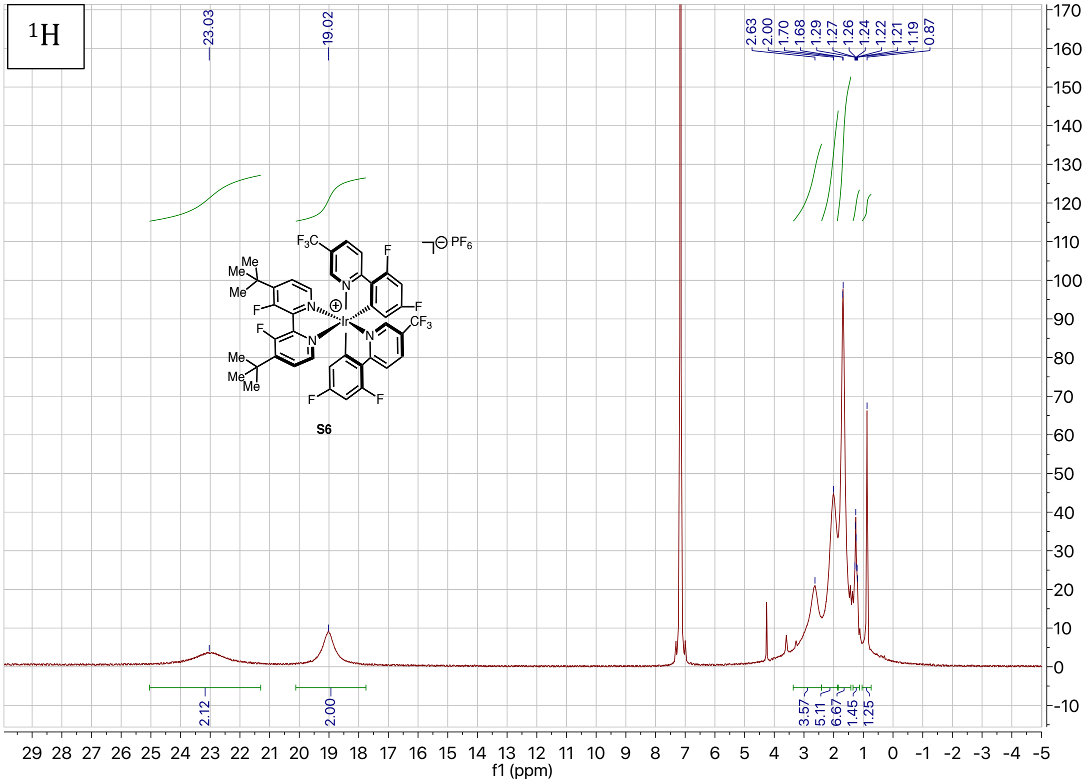
1H
23.03
19.02
2.63
2.00
1.70
1.68
1.29
1.27
1.26
1.24
1.22
1.21
1.19
0.87
2.12
2.00
3.57
5.11
6.67
1.45
1.25
S6
f1 (ppm)

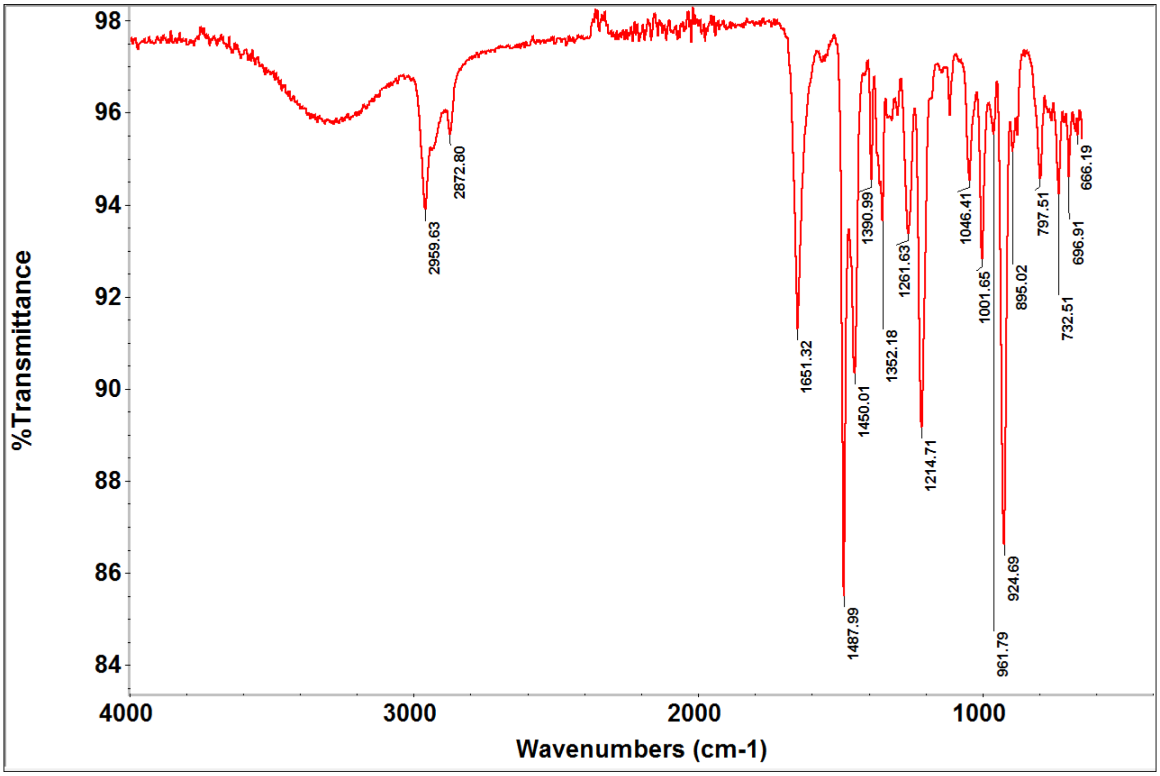
%Transmittance
Wavenumbers (cm-1)
2959.63
2872.80
1651.32
1487.99
1450.01
1390.99
1352.18
1261.63
1214.71
1046.41
1001.65
961.79
924.69
895.02
797.51
732.51
696.91
666.19

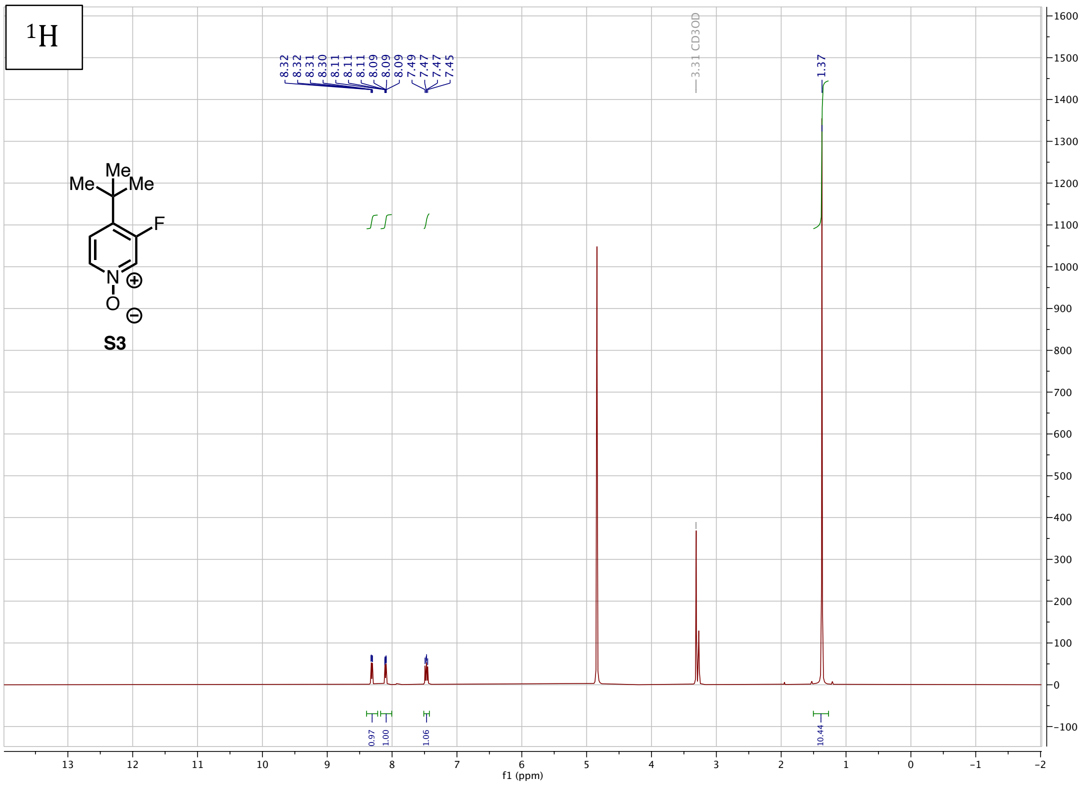

1H
8.32
8.32
8.31
8.30
8.11
8.11
8.09
8.09
7.49
7.47
7.47
7.45
3.31 CD3OD
1.37
Me
Me
Me
F
N
O
S3
0.97
1.00
1.06
10.44
f1 (ppm)

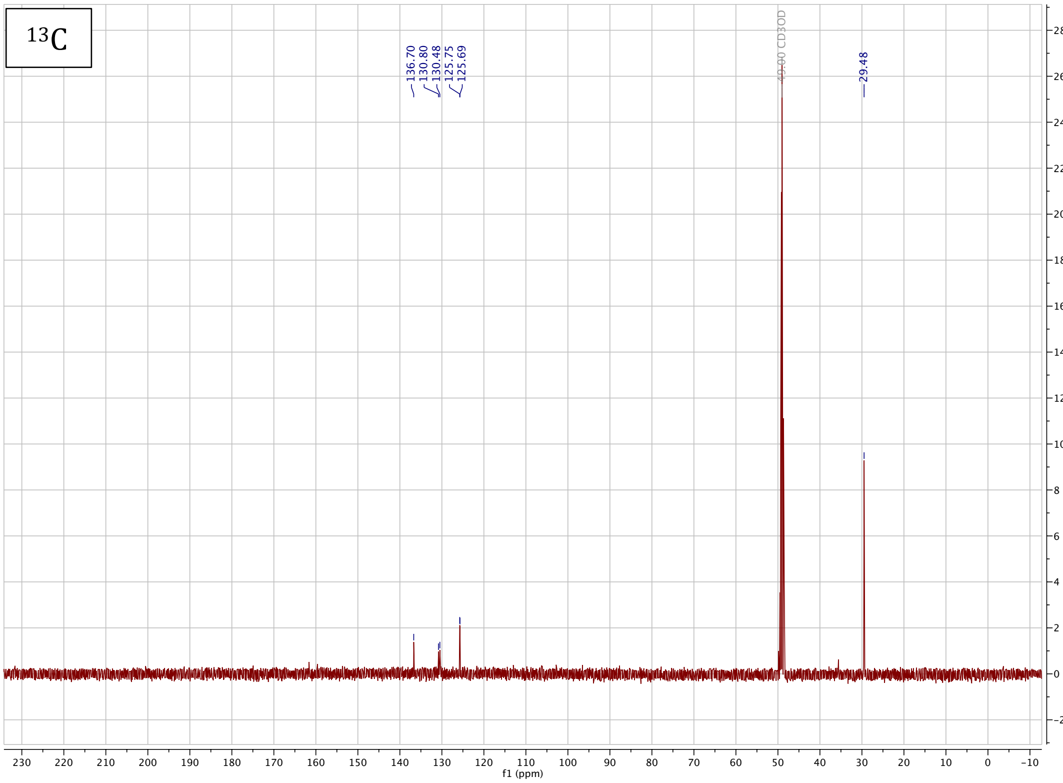

13C
136.70
130.80
130.48
125.75
125.69
49.00 CD3OD
29.48
f1 (ppm)

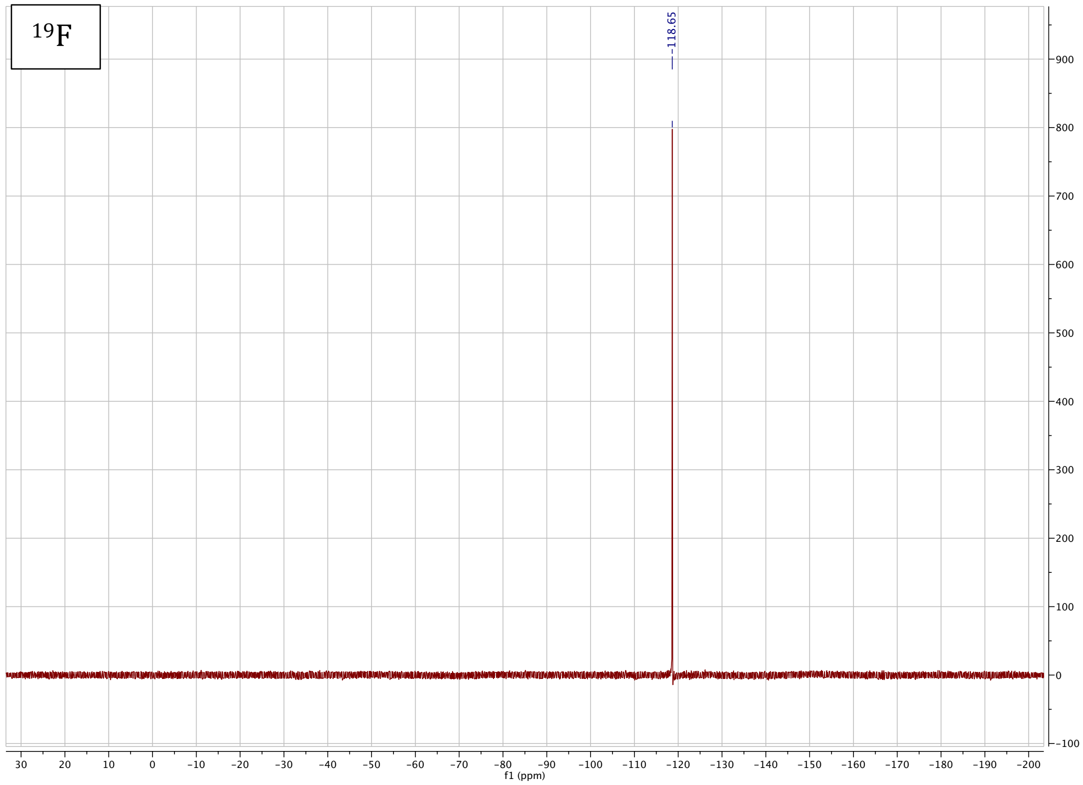
^{19}F
-118.65
f1 (ppm)

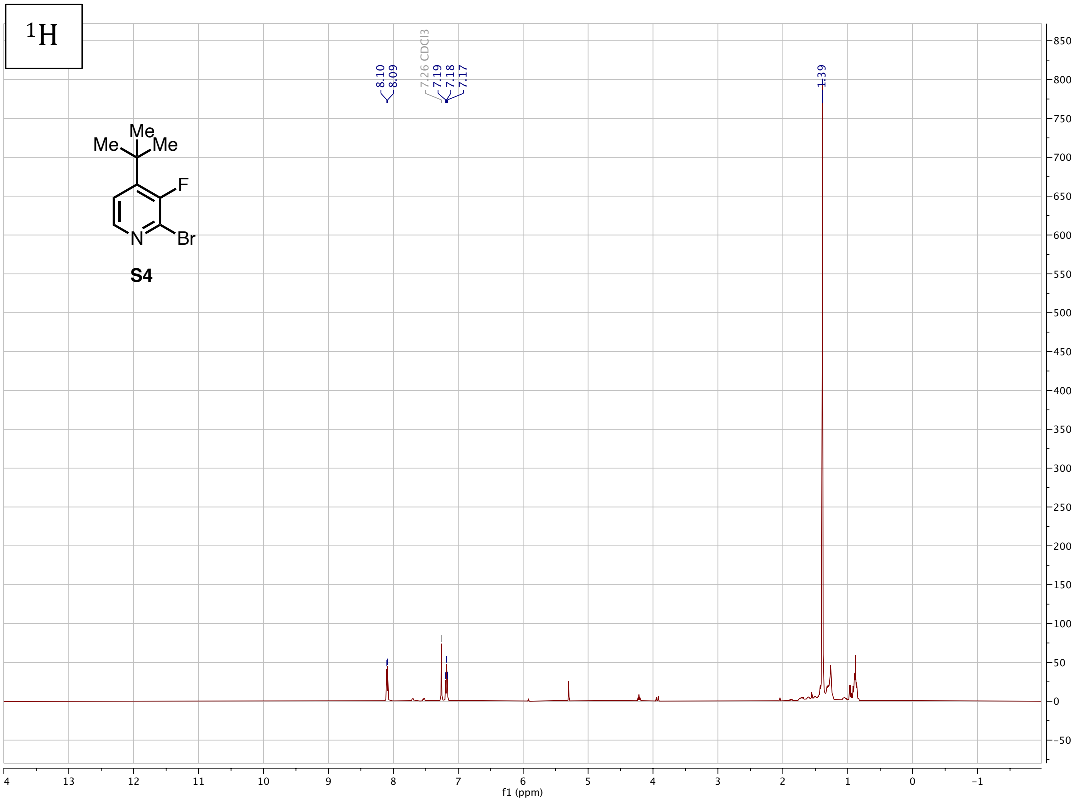

1H
Me
Me
Me
F
N
Br
S4
8.10
8.09
7.26 CDCl3
7.19
7.18
7.17
1.39
f1 (ppm)

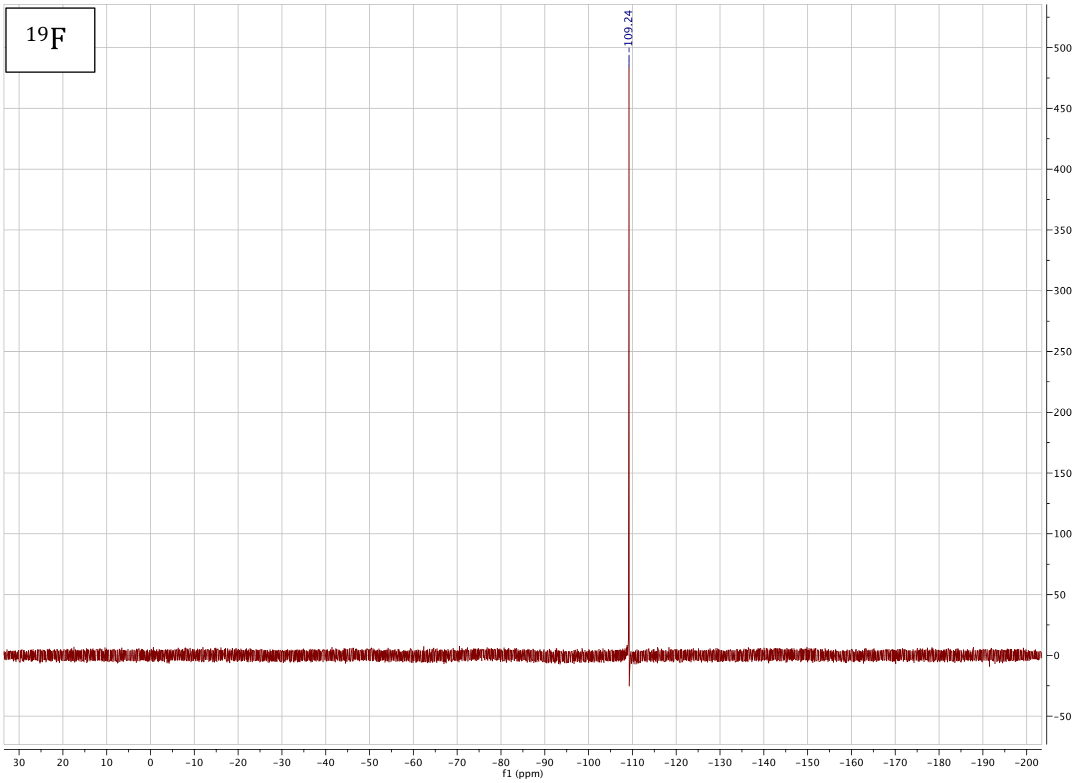

19F
-109.24
f1 (ppm)

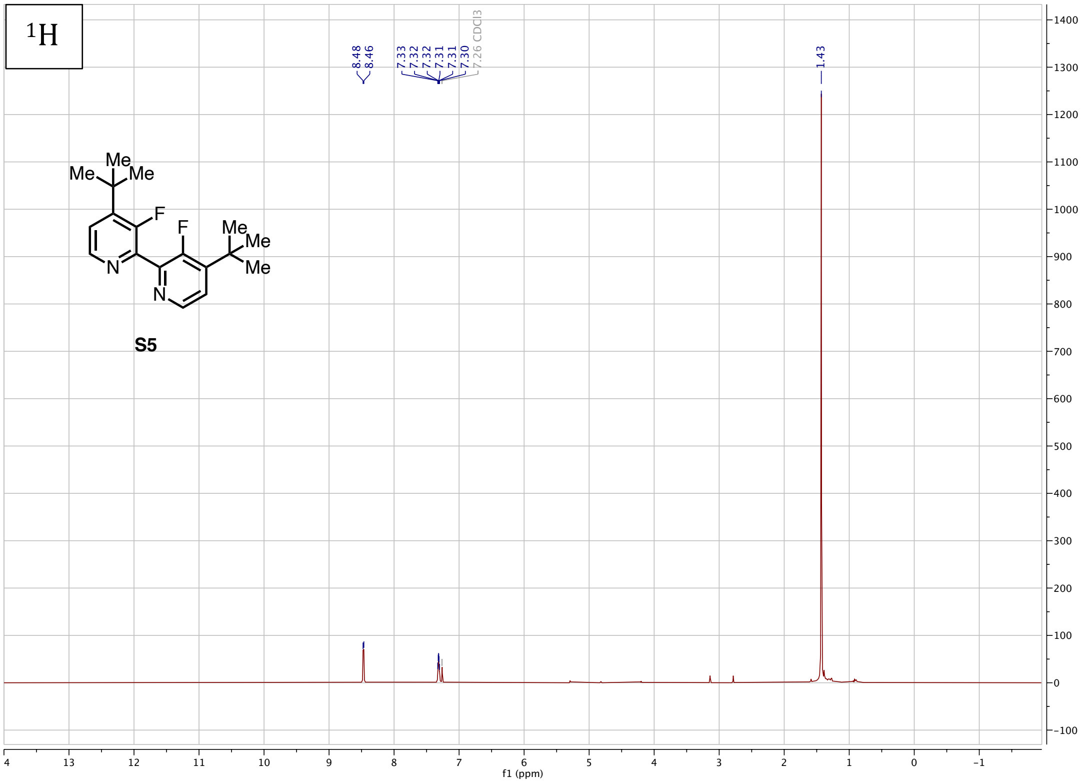

1H
Me
Me
Me
F
F
Me
Me
Me
N
N
S5
8.48
8.46
7.33
7.32
7.32
7.31
7.31
7.30
7.26 CDCl3
1.43
f1 (ppm)

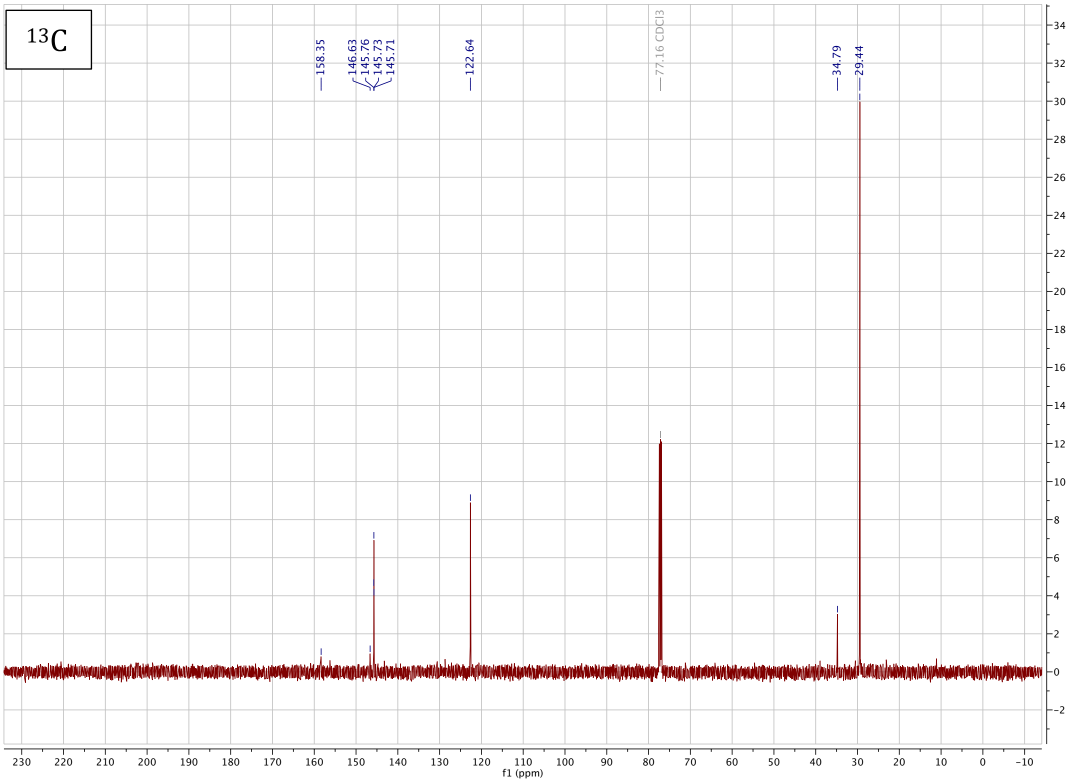

13C
158.35
146.63
145.76
145.73
145.71
122.64
77.16 CDCl3
34.79
29.44
f1 (ppm)

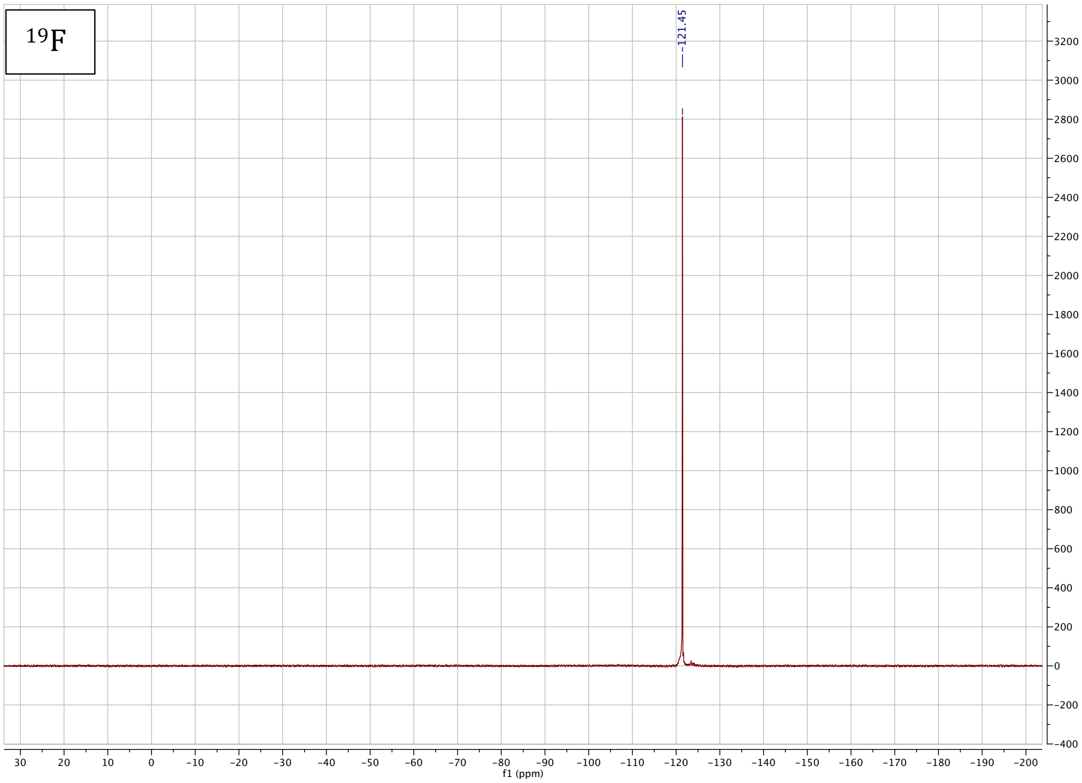

^{19}F
-121.45
f1 (ppm)

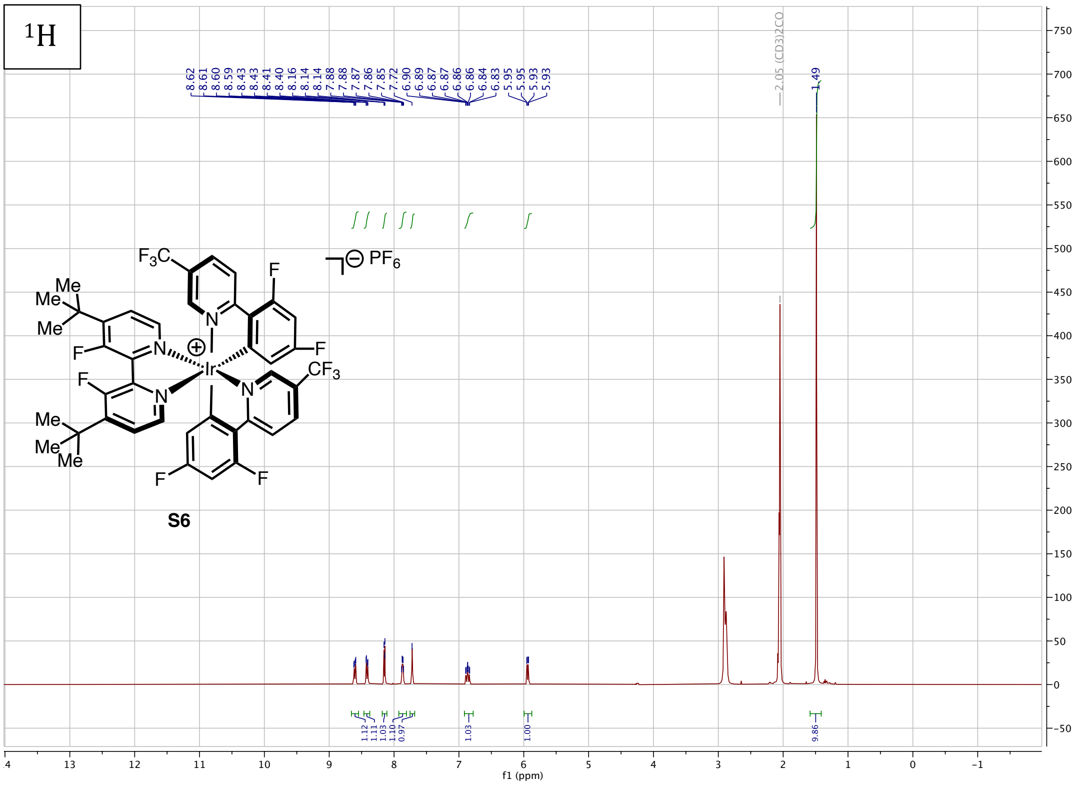

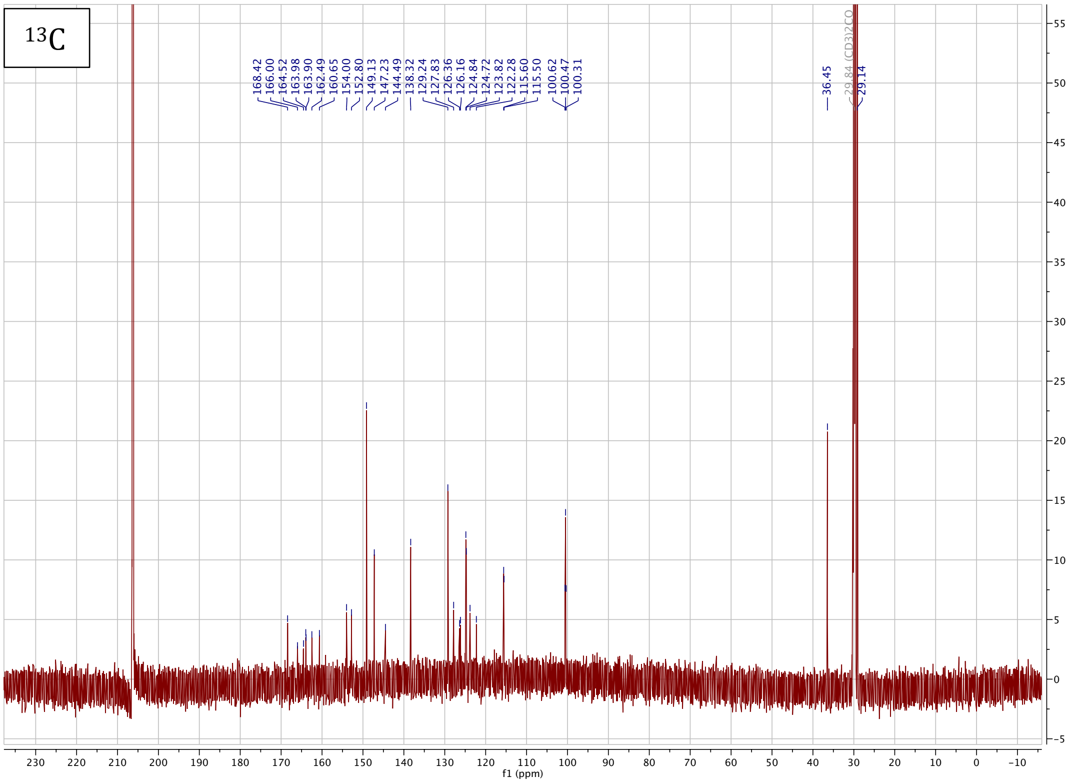

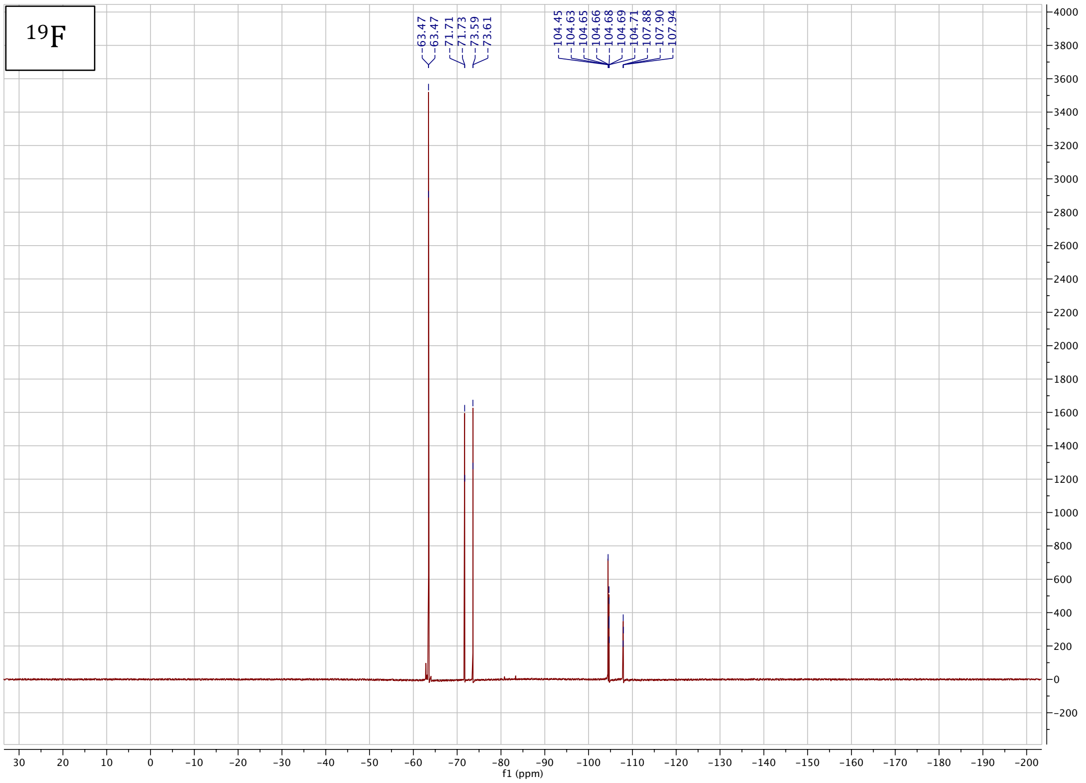
19F
-63.47
-63.47
-71.71
-71.73
-73.59
-73.61
-104.45
-104.63
-104.65
-104.66
-104.68
-104.69
-104.71
-107.88
-107.90
-107.94
f1 (ppm)

X) NMR Spectra of Products:

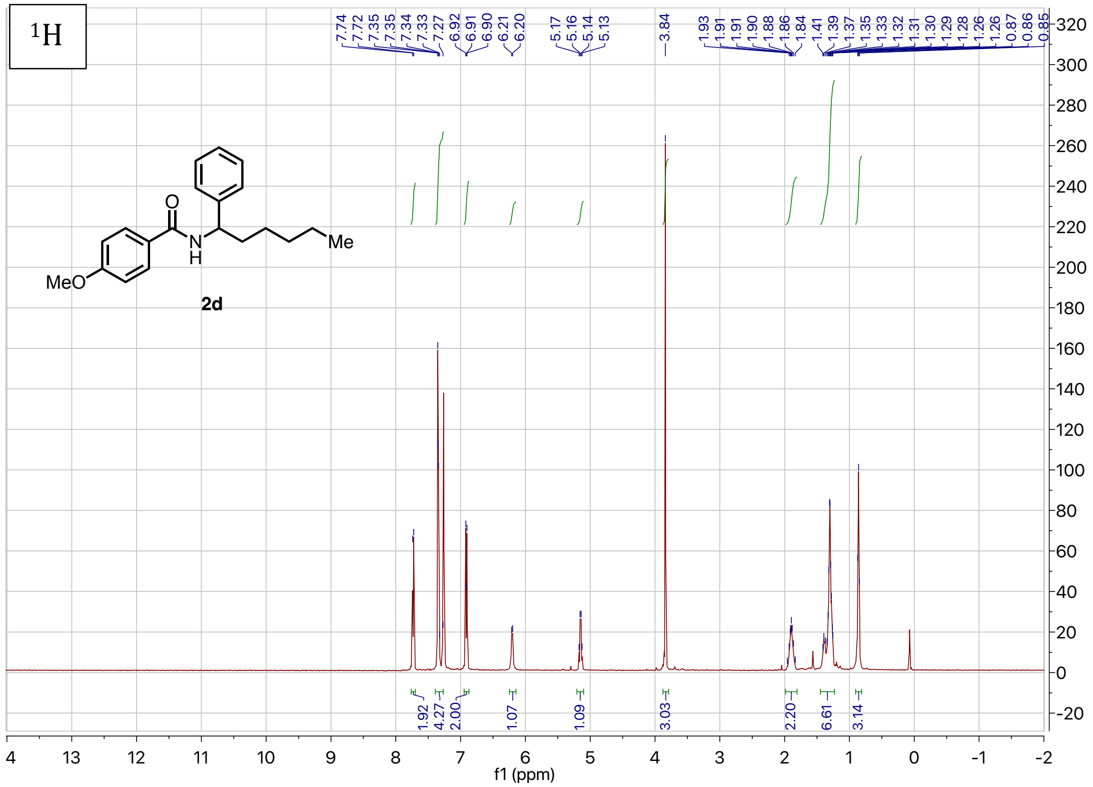

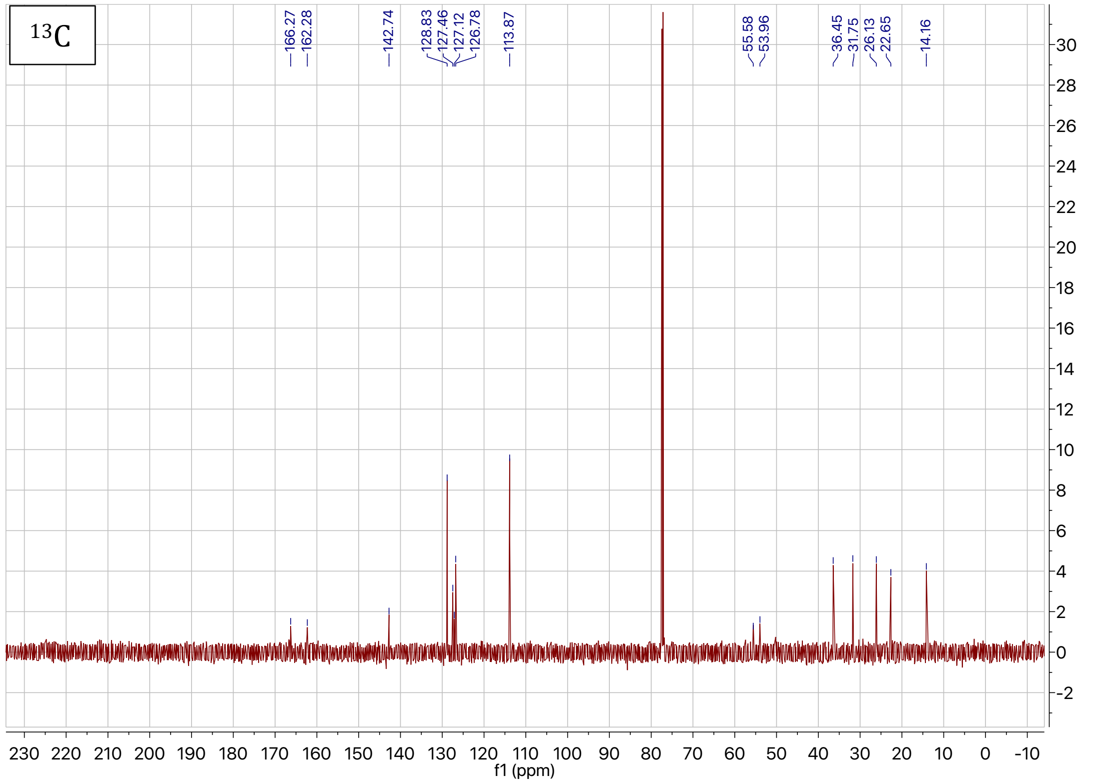

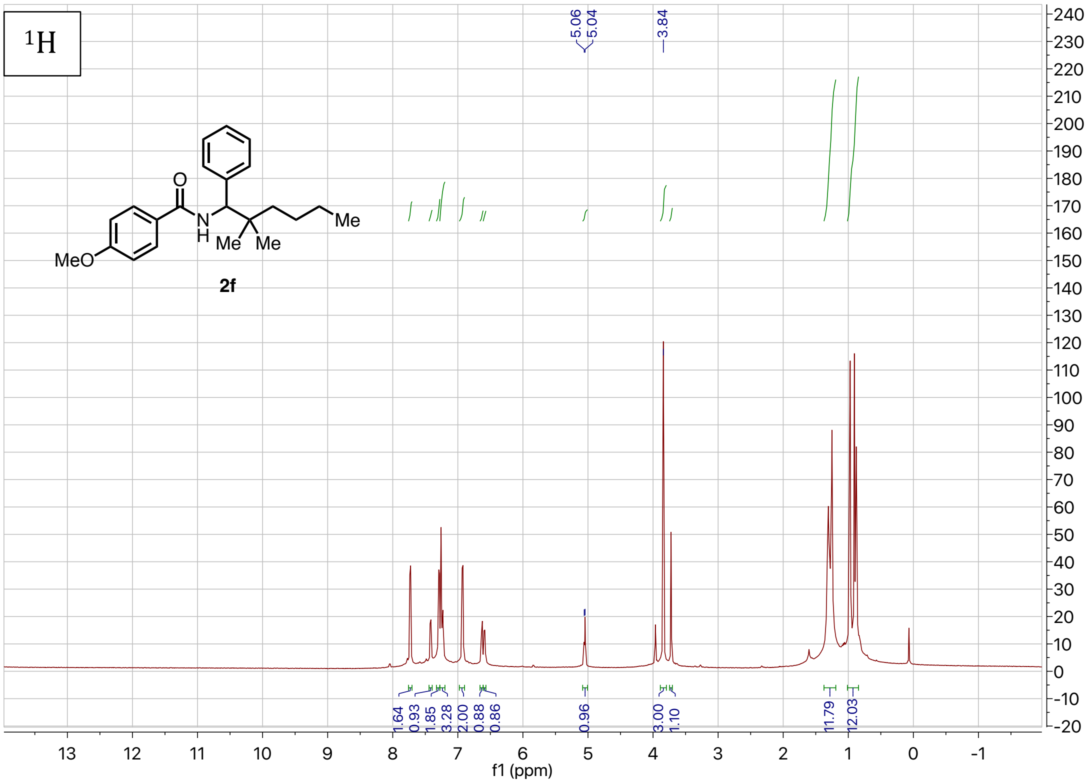

1H
O
N
H
Me
Me
Me
MeO
2f
5.06
5.04
3.84
1.64
0.93
1.85
3.28
2.00
0.88
0.86
0.96
3.00
1.10
11.79
12.03
f1 (ppm)

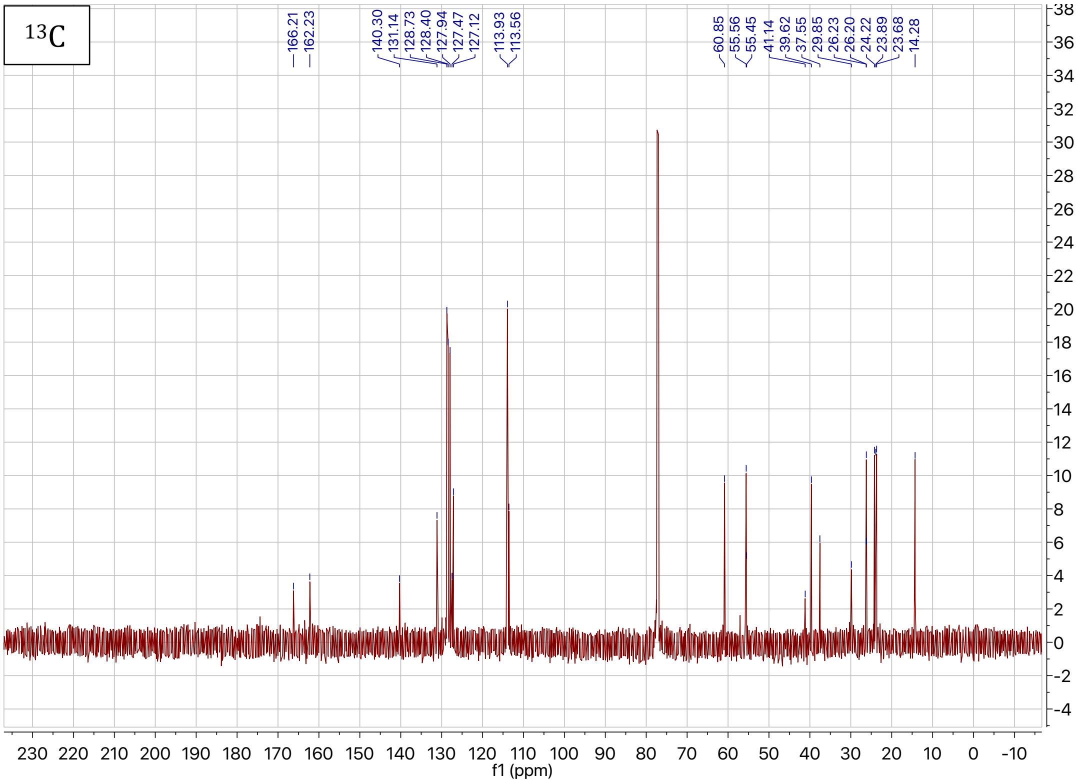

13C
166.21
162.23
140.30
131.14
128.73
128.40
127.94
127.47
127.12
113.93
113.56
60.85
55.56
55.45
41.14
39.62
37.55
29.85
26.23
26.20
24.22
23.89
23.68
14.28
f1 (ppm)

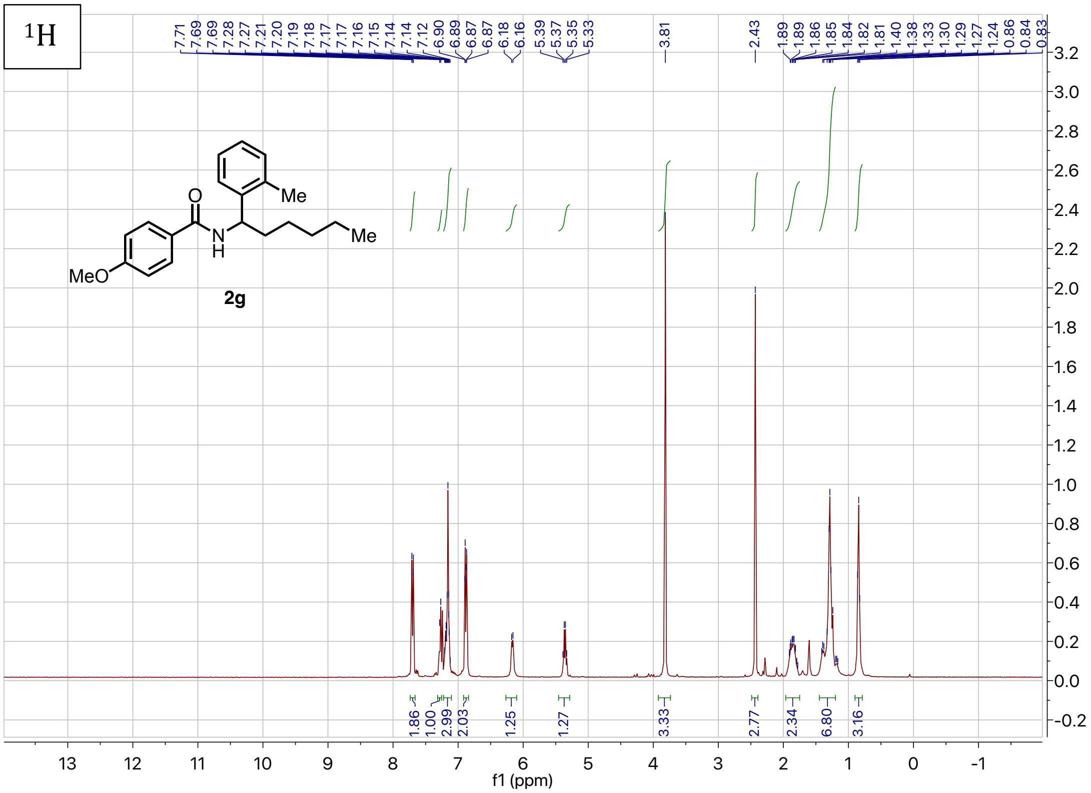

1H
O
N
H
Me
Me
MeO
2g
7.71
7.69
7.69
7.28
7.27
7.21
7.20
7.19
7.18
7.17
7.17
7.16
7.15
7.14
7.14
7.12
6.90
6.89
6.87
6.87
6.18
6.16
5.39
5.37
5.35
5.33
3.81
2.43
1.89
1.89
1.86
1.85
1.84
1.82
1.81
1.40
1.38
1.33
1.30
1.29
1.27
1.24
0.86
0.84
0.83
1.86
1.00
2.99
2.03
1.25
1.27
3.33
2.77
2.34
6.80
3.16
f1 (ppm)

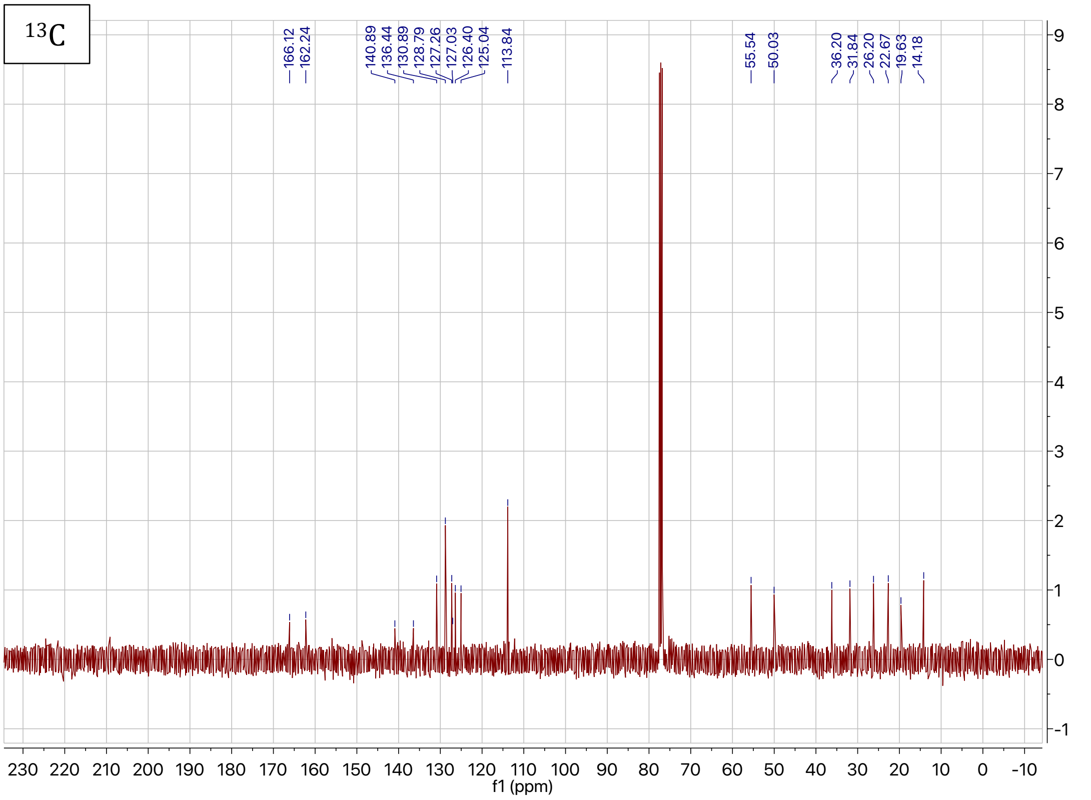

13C
166.12
162.24
140.89
136.44
130.89
128.79
127.26
127.03
126.40
125.04
113.84
55.54
50.03
36.20
31.84
26.20
22.67
19.63
14.18
f1 (ppm)

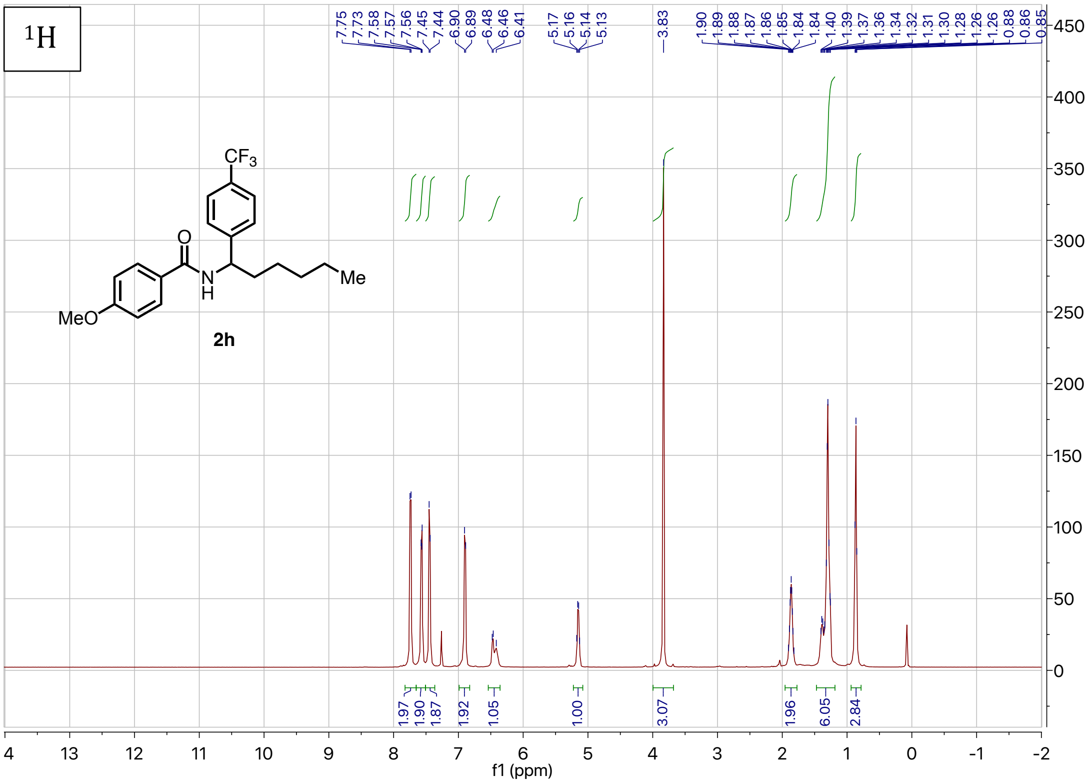

1H
CF3
O
N
H
MeO
Me
2h
7.75 7.73 7.58 7.57 7.56 7.45 7.44 6.90 6.89 6.48 6.46 6.41 5.17 5.16 5.14 5.13 3.83 1.90 1.89 1.88 1.87 1.86 1.85 1.84 1.84 1.40 1.39 1.37 1.36 1.34 1.32 1.31 1.30 1.28 1.26 1.26 0.88 0.86 0.85
1.97 1.90 1.87 1.92 1.05 1.00 3.07 1.96 6.05 2.84
f1 (ppm)

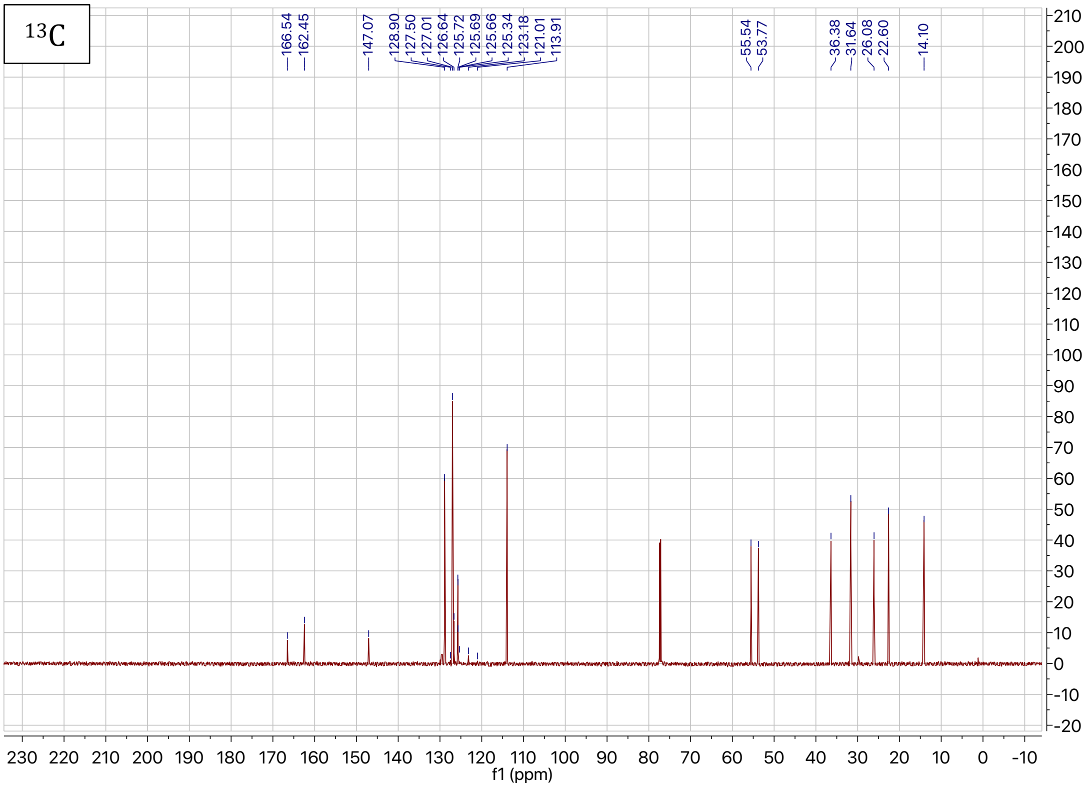

13C
166.54 162.45 147.07 128.90 127.50 127.01 126.64 125.72 125.69 125.66 125.34 123.18 121.01 113.91 55.54 53.77 36.38 31.64 26.08 22.60 14.10
f1 (ppm)

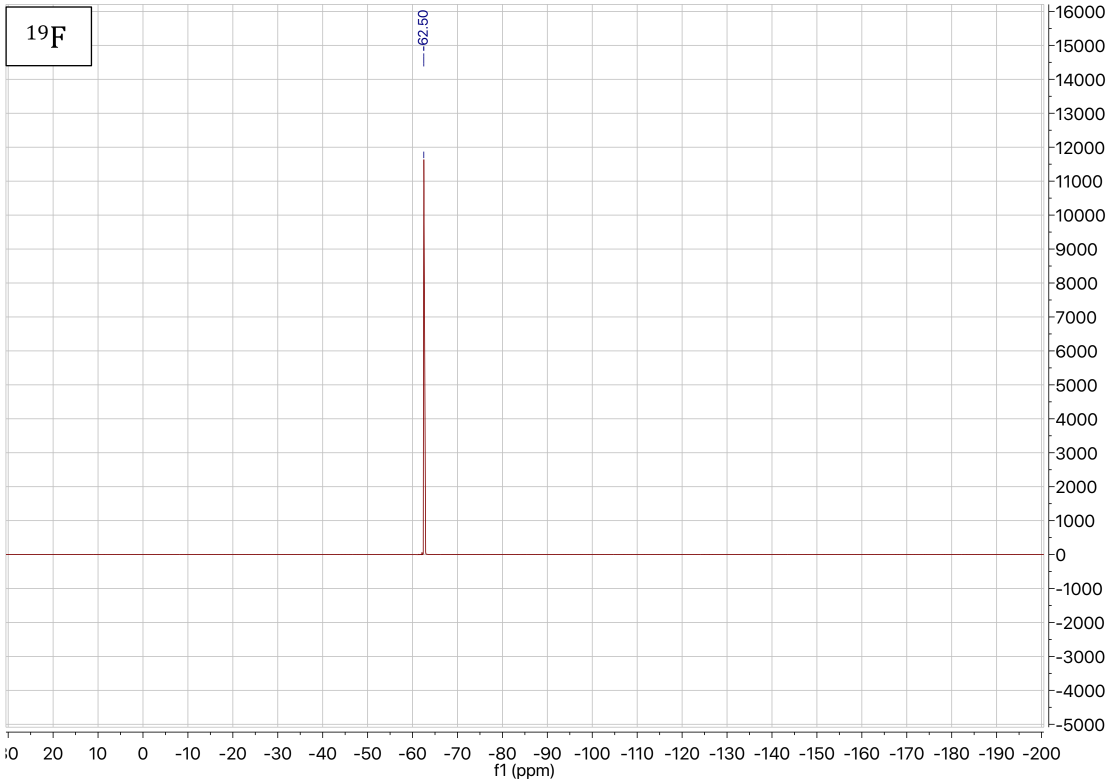
19F
-62.50
f1 (ppm)

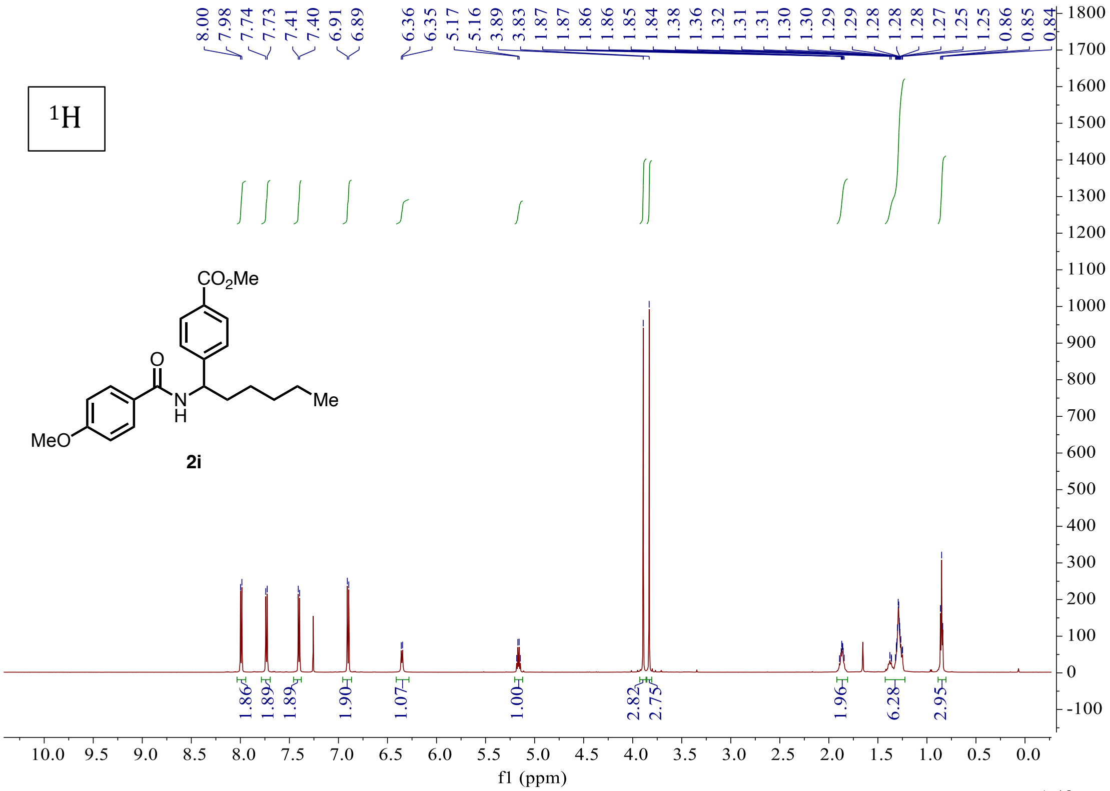

1H
CO2Me
O
N
H
Me
MeO
2i
8.00 7.98 7.74 7.73 7.41 7.40 6.91 6.89 6.36 6.35 5.17 5.16 3.89 3.83 1.87 1.87 1.86 1.86 1.85 1.84 1.38 1.36 1.32 1.31 1.31 1.30 1.29 1.29 1.28 1.28 1.28 1.27 1.25 1.25 0.86 0.85 0.84
1.86 1.89 1.89 1.90 1.07 1.00 2.82 2.75 1.96 6.28 2.95
f1 (ppm)

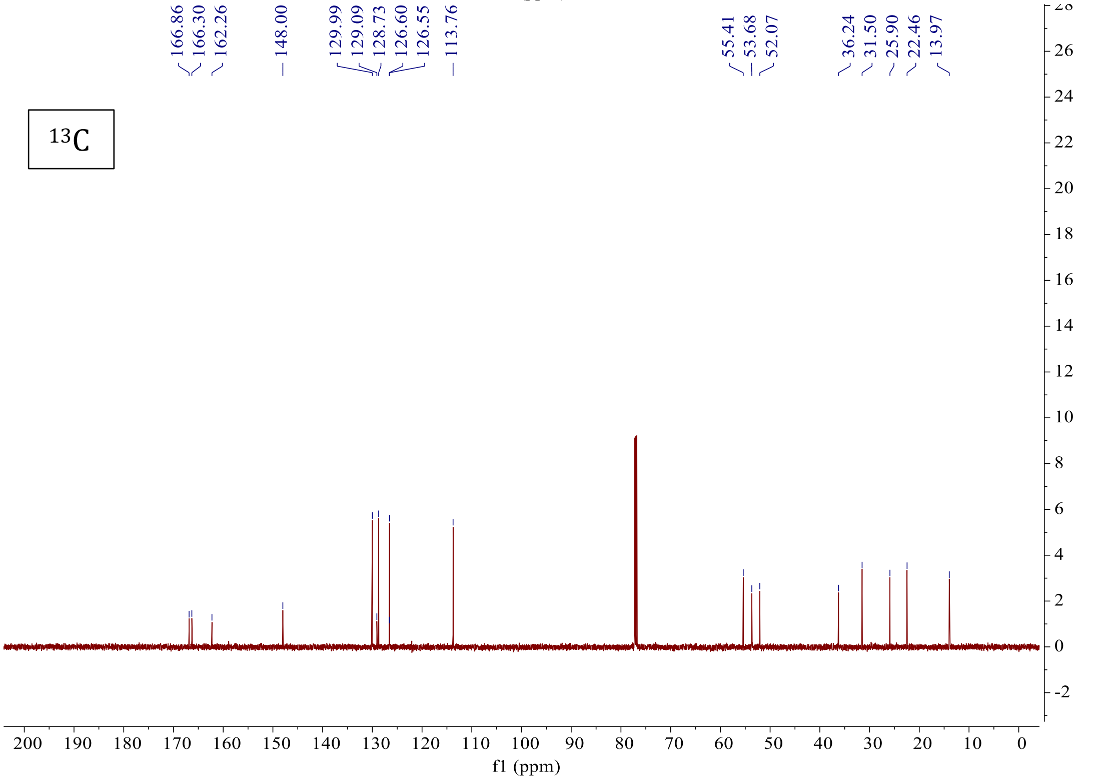

13C
166.86 166.30 162.26 148.00 129.99 129.09 128.73 126.60 126.55 113.76 55.41 53.68 52.07 36.24 31.50 25.90 22.46 13.97
f1 (ppm)

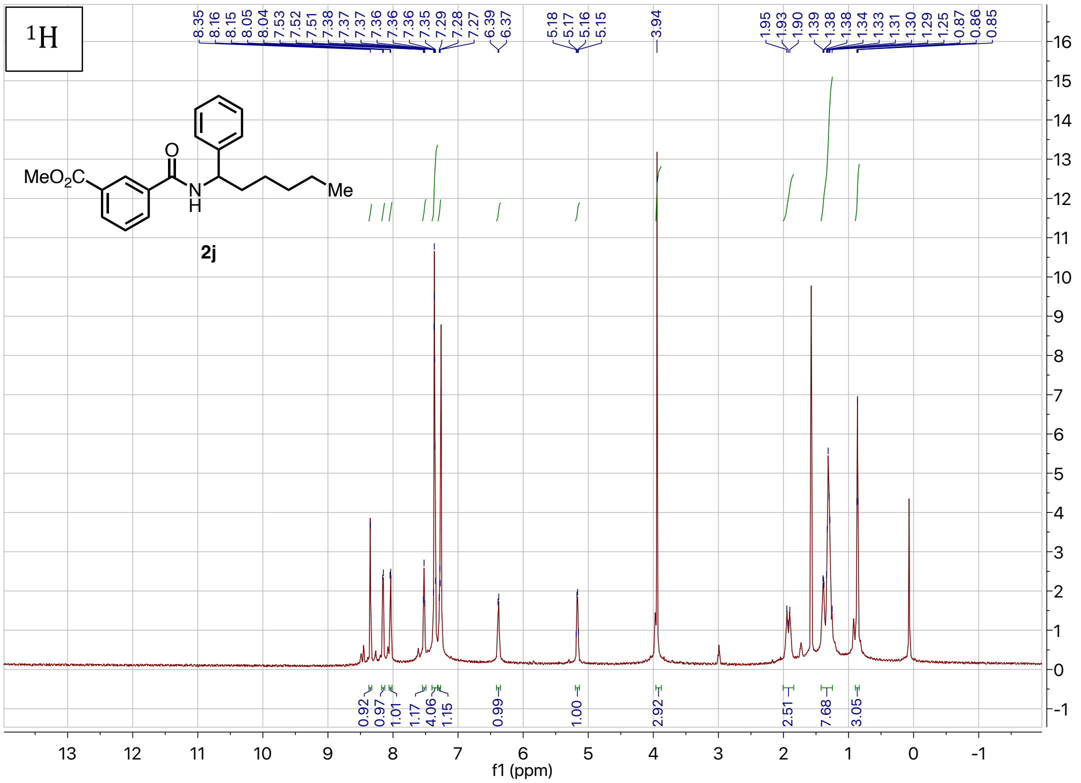

1H
MeO2C
O
N
H
Me
2j
8.35 8.16 8.15 8.05 8.04 7.53 7.52 7.51 7.38 7.37 7.37 7.36 7.36 7.36 7.35 7.29 7.28 7.27 6.39 6.37 5.18 5.17 5.16 5.15 3.94 1.95 1.93 1.90 1.39 1.38 1.38 1.34 1.33 1.31 1.30 1.29 1.25 0.87 0.86 0.85
0.92 0.97 1.01 1.17 4.06 1.15 0.99 1.00 2.92 2.51 7.68 3.05
f1 (ppm)

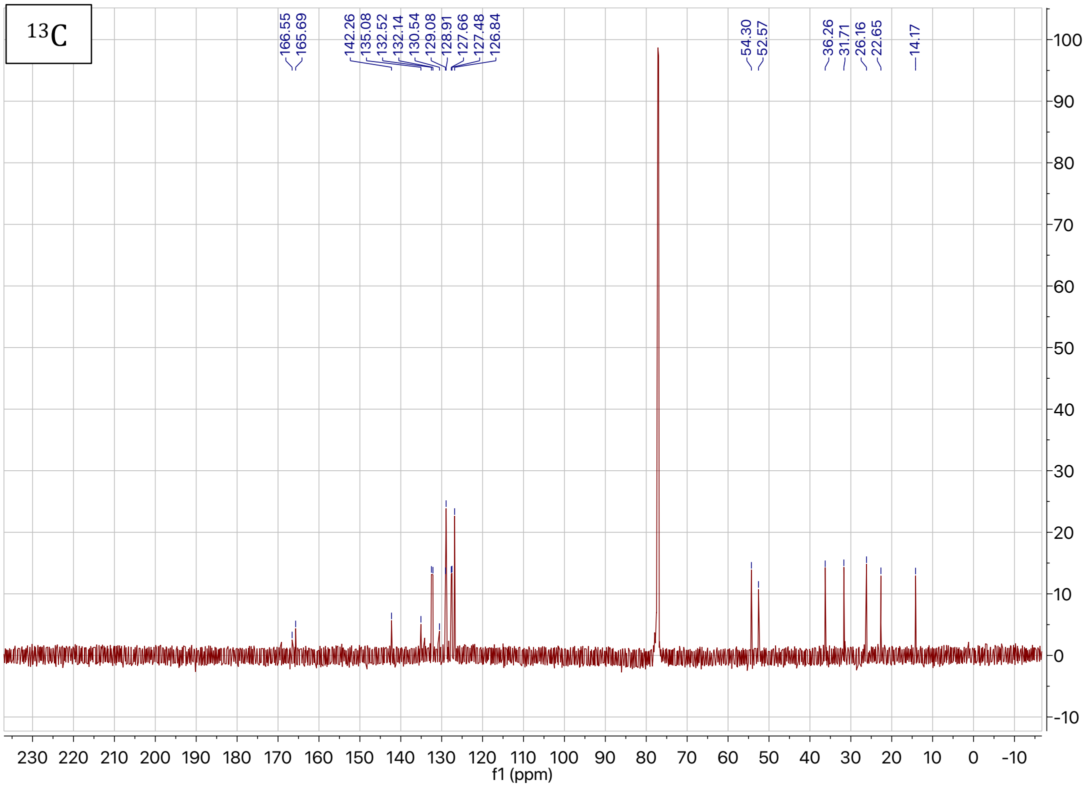

13C
166.55 165.69 142.26 135.08 132.52 132.14 130.54 129.08 128.91 127.66 127.48 126.84 54.30 52.57 36.26 31.71 26.16 22.65 14.17
f1 (ppm)

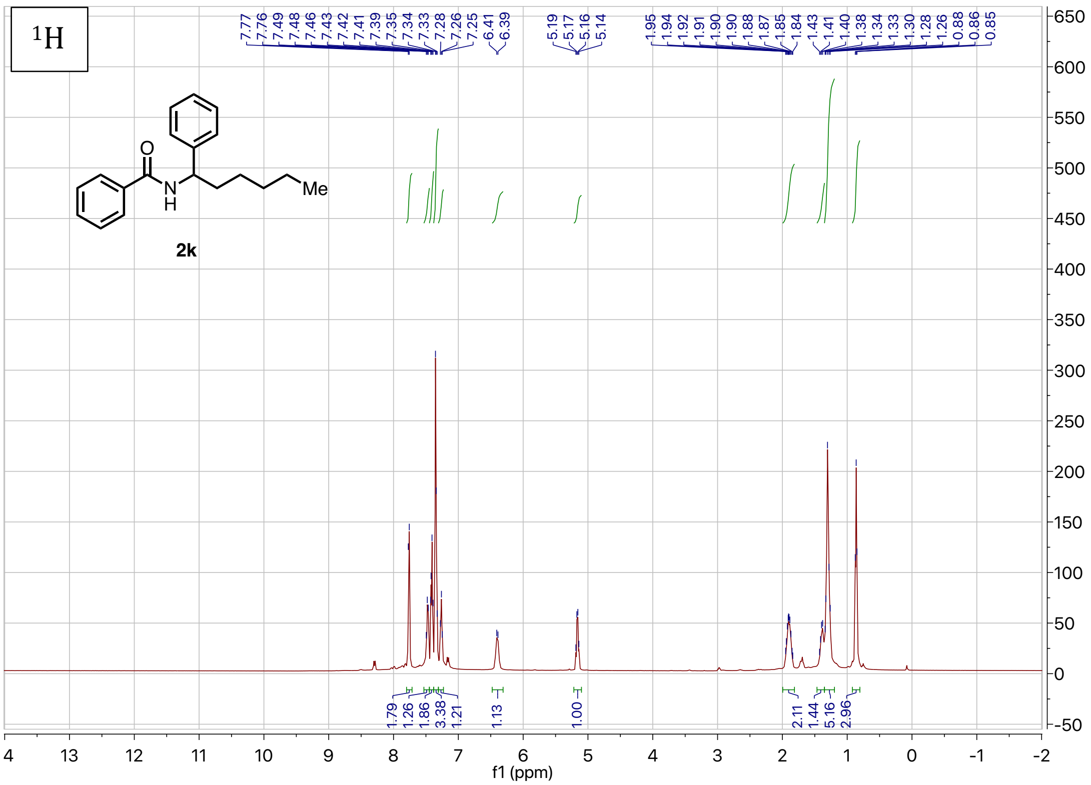
1H
Me
2k
f1 (ppm)

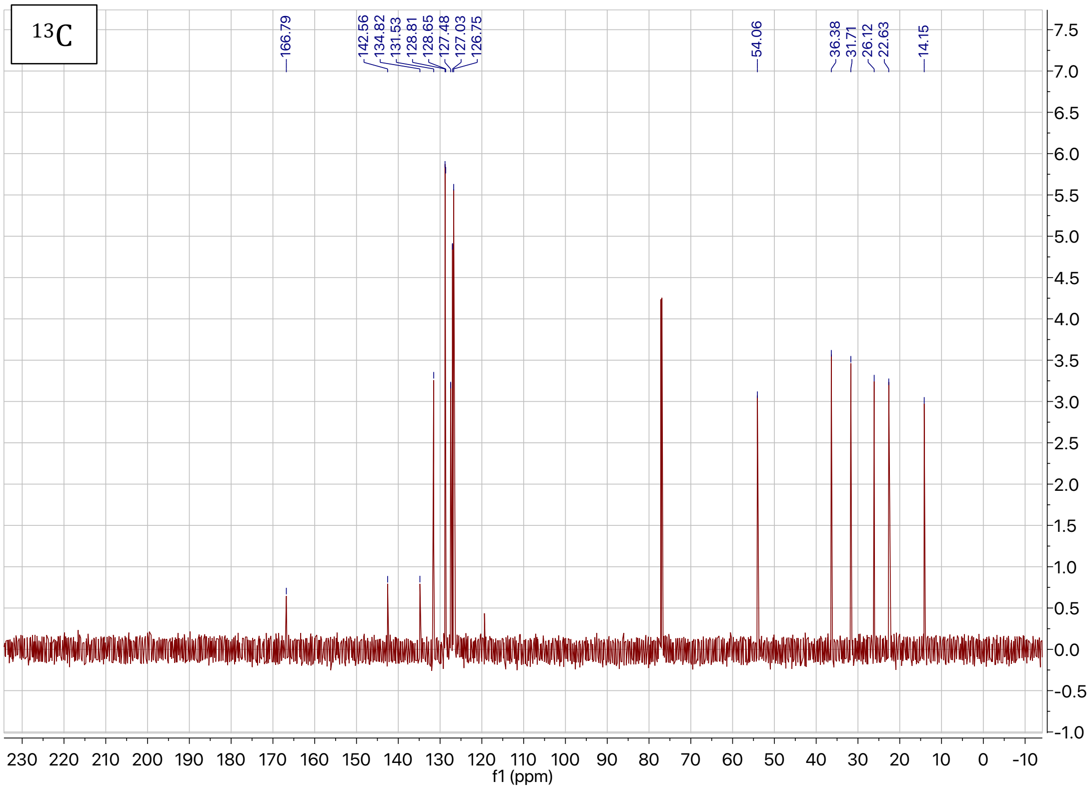
13C
f1 (ppm)

XI) Reference:

1. (a) G. J. Choi, Q. Zhu, D. C. Miller, C. J. Gu and R. R. Knowles, *Nature*, **2016**, *539*, 268-271; (b) L. Li, M. Zeng and S. B. Herzon, *Angew. Chem. Int. Ed. Engl.*, **2014**, *53*, 7892-7895.
2. P. Querard, I. Perepichka, E. Zysman-Colman and C. J. Li, *Beilstein J. Org. Chem.*, **2016**, *12*, 2636-2643.
3. B. P. Woods, M. Orlandi, C.-Y. Huang, M. S. Sigman and A. G. Doyle, *J. Am. Chem. Soc.*, **2017**, *139*, 5688-5691.
4. C. Martinez and K. Muniz, *Angew. Chem. Int. Ed. Engl.*, **2015**, *54*, 8287-8291.
5. A. S. K. Hashmi, M. C. Blanco Jaimes, A. M. Schuster and F. Rominger, *J. Org. Chem.*, **2012**, *77*, 6394-6408.
6. Y. Sawama, H. Sajiki, S. Asai, K. Ban and Y. Monguchi, *Synlett*, **2017**, *29*, 322-325.
7. T. Higuchi, R. Tagawa, A. Iimuro, S. Akiyama, H. Nagae and K. Mashima, *Chem. – Eur, J.*, **2017**, *23*, 12795-12804.
8. L. Ju, Q. Lin, N. J. LiBretto, C. L. Wagner, C. T. Hu, J. T. Miller and T. Diao, *J. Am. Chem. Soc.*, **2021**, *143*, 14458-14463.
9. D. Joseph, M. S. Park and S. Lee, *Org. Biomol. Chem.*, **2021**, *19*, 6227-6232.
10. S. Sharma, N. W. Buchbinder, W. M. Braje and S. Handa, *Org. Lett.*, **2020**, *22*, 5737-5740.
11. A. W. Rand, H. Yin, L. Xu, J. Giacoboni, R. Martin-Montero, C. Romano, J. Montgomery and R. Martin, *ACS Catalysis*, **2020**, 1*0*, 4671- 4676.
12. M. A. Ashley, C. Yamauchi, J. C. K. Chu, S. Otsuka, H. Yorimitsu and T. Rovis, *Angew. Chem. Int. Ed. Engl.*, **2019**, *58*, 4002-4006.
13. C. M. Morton, Q. Zhu, H. Ripberger, L. Troian-Gautier, Z. S. D. Toa, R. R. Knowles and E. J. Alexanian, *J. Am. Chem. Soc.*, **2019**, *141*, 13253-13260.
